# Levels, Trends, and Inequalities in Using Institutional Delivery Services in Low- and Middle-Income Countries: A Stratified Analysis by Facility Type

**DOI:** 10.9745/GHSP-D-20-00533

**Published:** 2021-03-31

**Authors:** Md. Mehedi Hasan, Ricardo J. Soares Magalhaes, Yaqoot Fatima, Saifuddin Ahmed, Abdullah A. Mamun

**Affiliations:** a Institute for Social Science Research, The University of Queensland, Indooroopilly, Queensland, Australia.; b ARC Centre of Excellence for Children and Families over the Life Course (The Life Course Centre), The University of Queensland, Indooroopilly, Queensland, Australia.; c UQ Spatial Epidemiology Laboratory, School of Veterinary Science, The University of Queensland, Gatton, Australia.; d UQ Children's Health and Environment Program, Child Health Research Centre, The University of Queensland, South Brisbane, Australia.; e Centre for Rural and Remote Health, James Cook University, Mount Isa, Australia.; f Department of Population, Family and Reproductive Health, Johns Hopkins Bloomberg School of Public Health, Baltimore, MD, USA.; g Bill and Melinda Gates Institute for Population and Reproductive Health, Johns Hopkins Bloomberg School of Public Health, Baltimore, MD, USA.

## Abstract

**Introduction:**

To ensure equitable and accessible services and improved utilization of institutional delivery it is important to identify what progress has been achieved, whether there are vulnerable and disadvantaged groups that need specific attention and what are the key factors affecting the utilization of institutional delivery services. In this study, we examined levels, trends, and inequalities in the utilization of institutional delivery services in low- and middle-income countries.

**Methods:**

We used nationally representative cross-sectional data from Demographic and Health Surveys (DHS) conducted during 1990–2018. Bayesian linear regression analysis was performed.

**Results:**

Among 74 countries, the utilization of institutional delivery services ranged from 23.7% in Chad to 100% in Ukraine and Armenia (with >90% in 19 countries and <50% in 13 countries) during the latest DHS rounds. Trend analysis in 63 countries with at least 2 surveys showed that the utilization of institutional delivery services increased in 60 countries during 1990–2018, with the highest increase being in Cambodia (18.3%). During this period, the utilization of institutional delivery services increased in 90.3% of countries among the richest, 95.2% of countries in urban, and 84.1% of countries among secondary+ educated women. The utilization of institutional delivery services was higher among wealthiest, urban, and secondary+ educated women compared to their counterparts. Greater utilization of private facilities for delivery was observed in women from the highest income group and urban communities, whereas highest utilization of public facilities was observed for women from the lowest income group and rural communities.

**Conclusions:**

The utilization of institutional delivery services varied substantially between and within countries over time. Significant disparities in service utilization identified in this study highlight the need for tailored support for women from disadvantaged and vulnerable groups.

## Abstract (precis)

Despite improvements in the use of institutional delivery services around the world, progress has not been uniform across low- and middle-income countries. Persistent and growing inequalities in the utilization of institutional delivery services warrant the attention of policy makers for further investments and policy reviews.

## Teaser

**Key Findings:**

- Progress toward improving the utilization of institutional delivery services was not uniform across low- and middle-income countries (LMICs) and across subpopulations within LMICs irrespective of public and private health facilities.
- Wealth, place of residence, and education-based inequalities in the utilization of institutional delivery services are widening in many LMICs, which warrants the attention of policy makers for further investments and policy reviews.

**Key Implications:**

- Program managers and policy makers should give special priority to people who are poorest, live in rural areas, and have low education when designing appropriate interventions for increasing institutional delivery service coverage, irrespective of public and private facilities.
- Appropriate and tailored interventions covering the disadvantaged countries and marginalized populations within countries may help countries to achieve the global target of “leaving no one behind” for the utilization of institutional delivery services by 2030.

## INTRODUCTION

Institutional delivery is a necessary intervention to reduce delivery-related avoidable maternal and infant mortality.^1^ Between 1990 and 2015, more than 10 million women died globally due to pregnancy and childbirth-related complications.^2^ Globally, 2.6 million newborns died in 2016, approximately 7,000 per day,^3^ and almost all (99%) of these potentially preventable deaths occurred in low- and middle-income countries (LMICs).^4^ Pregnancy-related complications that lead to maternal mortality may occur during or shortly after childbirth.^5^ In LMICs, direct obstetric complications during childbirth were responsible for 70% of maternal deaths.^6^ Timely access to facility-based births save the lives of many mothers and newborns.^7^ In high-income countries, maternal mortality can be further reduced with increased rates of institutional delivery.^8^ In many LMICs, due to the geographical barriers in accessing services and the presence of cultural issues, women are accustomed to delivering babies at home, which leads to low utilization of institutional delivery services. To ensure equitable and accessible institutional delivery services, identifying vulnerable groups and populations within countries is crucial so that customized interventions can be developed and delivered.

The United Nations' Millennium Development Goals (MDGs) had a priority to improve maternal health and had set a target of reducing maternal mortality by three-quarters between 1990 and 2015 (MDG 5, target 5.A).^9^ Several initiatives have been introduced to achieve this target, including increased utilization of institutional delivery services.^4^ Earlier evidence showed improvements in the coverage of institutional delivery services in LMICs during the MDG era.^10^ During the same period, the world made remarkable progress in reducing maternal mortality by 43.9% from 385 deaths per 100,000 live births in 1990 to 216 in 2015.^2^ However, this progress was uneven across countries and different populations within countries, and significant progress gaps consequently exist between populations.

To reduce such gaps, the global agenda shifted from MDGs to Sustainable Development Goals (SDGs). The highest priority of the SDG targets (target 3.8) is achieving universal health coverage (UHC), which means “all individuals and communities receive the health services they need without suffering financial hardship.”^11^ Given the role of financial hardship in service utilization, it is also important to know which facility services (public or private) are increasing in LMICs and whether all people, irrespective of sociodemographic conditions, have equal access to these facilities. At the global level, evidence suggests an increasing trend in the utilization of institutional delivery services in sub-Saharan Africa,^12^^,^^13^ notably higher utilization by women from high-income groups residing in urban areas,^14^ as well as increasing use of private facilities for institutional delivery.^15^

However, comprehensive information is lacking on how socioeconomic and demographic disparities are associated with access to institutional delivery services, which limits the design of effective interventions/strategies required for equitable services. In addition, the extent to which these disparities are prevalent in public and private facilities remains unclear. Trend analysis at national and subpopulation levels helps policy makers and program managers assess overall progress, quantify gaps, and identify priority groups to guide strategies/interventions, further accelerating progress toward saving millions of lives of mothers and newborns. Therefore, this study aimed to examine the levels and trends in the utilization of institutional delivery services between LMICs and across subpopulations within LMICs.

A lack of information on how socioeconomic and demographic disparities affect access to institutional delivery services limits the design of effective interventions and strategies.

## METHODS

### Data

This study used secondary data from large-scale, population-based, nationally representative repeated cross-sectional surveys conducted between 1990 and 2018 under the Demographic and Health Surveys (DHS) program.^16^ We extracted data from 74 LMICs across 5 DHS regions: sub-Saharan Africa (37 countries), South and Southeast Asia (12 countries), Central Asia (4 countries), North Africa-West Asia-Europe (10 countries), and Latin America and Caribbean (LAC; 11 countries). A detailed description of the surveyed country, survey year, and sample size is presented in the Supplement (Table S1).

### Outcome Variable

The outcome variable in our study was institutional delivery. We used DHS standard recode files (KR files) to construct the variable for institutional delivery based on the responses of participants. The DHS provided information for institutional delivery for children born in the past 5 years in most of the countries. However, for some countries such as Bangladesh, the information on institutional delivery services was only available for children born in the past 3 years. Therefore, to allow cross-country comparison, we defined institutional delivery services as the proportion of live births delivered in health facilities in the 3 years preceding the survey. We compared deliveries conducted in different types of health facilities (public versus private), and we particularly evaluated the proportion of deliveries that occurred in a public facility and those in a private facility. All calculations were conducted for live births.

### Statistical Analyses

We estimated the weighted prevalence of institutional delivery services as proportions from the original survey data for all survey years of each study country. The rates of delivery in public and private facilities were estimated using the same method. However, we examined the geographical variation in the utilization of institutional delivery services during the latest DHS round. We calculated the variation in the utilization of institutional delivery services across subgroups in terms of place of residence, education of women, age of women, and wealth quintiles that the DHS constructed based on household assets by principal component analysis.^17^

For this study, we dichotomized education as below secondary (no or primary education) and secondary+ (secondary or higher) education. Similarly, we categorized age as 15–19 years (adolescents) and 20–49 years (adults). Also, we used place of residence (categorized as rural and urban) and wealth quintiles (categorized as poorest [first quintile], poorer, middle, richer, and richest [fifth quintile]) that the DHS provided with the survey data. Notably, we restricted our analysis to the country level but not at a regional level for 2 reasons. First, some regions (e.g., Central Asia) had data for a limited number of countries and heterogeneity between survey years (arbitrary). Second, we were interested in assessing progress across individual countries so that country-level programs and policies could be implemented.

To examine trends, a Bayesian linear regression model that used a Markov Chain Monte Carlo algorithm of multiple imputations for missing data was applied to estimate the institutional delivery rates and trends from 1990 to 2018 (Supplement). We reported 95% credible intervals (CrI) drawn from Bayesian analysis along with these estimates. We used the same technique to examine trends in the utilization of institutional delivery services across various sociodemographic groups to explore the changes in the utilization of institutional delivery services across sociodemographic subpopulations. We also validated our estimates drawn from regression models with those drawn from the original microdata (Supplement, Table S2).

To measure inequalities in the utilization of institutional delivery services, we applied both absolute and relative measure of inequalities. We estimated absolute inequality by subtracting the rate of the institutional delivery services in the poorest quintile from the rate of the institutional delivery services in the richest quintile, of rural from urban, of below secondary education from secondary+ education, and of adolescent mothers 15–19 years of age from adult mothers 20–49 years of age. We calculated rate ratio by dividing the rate of the institutional delivery services in the richest quintile by the rate of the institutional delivery services in the poorest quintile, and similarly the rate in urban by rural, in secondary+ education by below secondary education, and in adult mothers by adolescent mothers. To quantify the changes in inequalities over time, we measured changes in absolute and relative inequalities in the utilization of institutional delivery services from the earliest and latest rounds of DHS for countries that had at least 2 survey data points.

We used Stata (version 15.1) and R (version 3.5) statistical software to analyze the data.

## RESULTS

### Sample Characteristics

We included a total of 1,538,486 live births, from 256 surveys conducted in 74 countries, to assess whether these births took place at a health facility or at home. For trend analysis, we considered a total of 245 surveys conducted in 63 countries that had data on institutional delivery for at least 2 DHS rounds (Supplement, Table S1). Overall, 23.1% of all live births were reported for women from the lowest quintile of wealth (poorest). The majority of birth data came from women living in rural areas (67.5%) and women with below secondary education (65.2%).

### Coverage in the Utilization of Institutional Delivery Services

**FIGURE 1:**
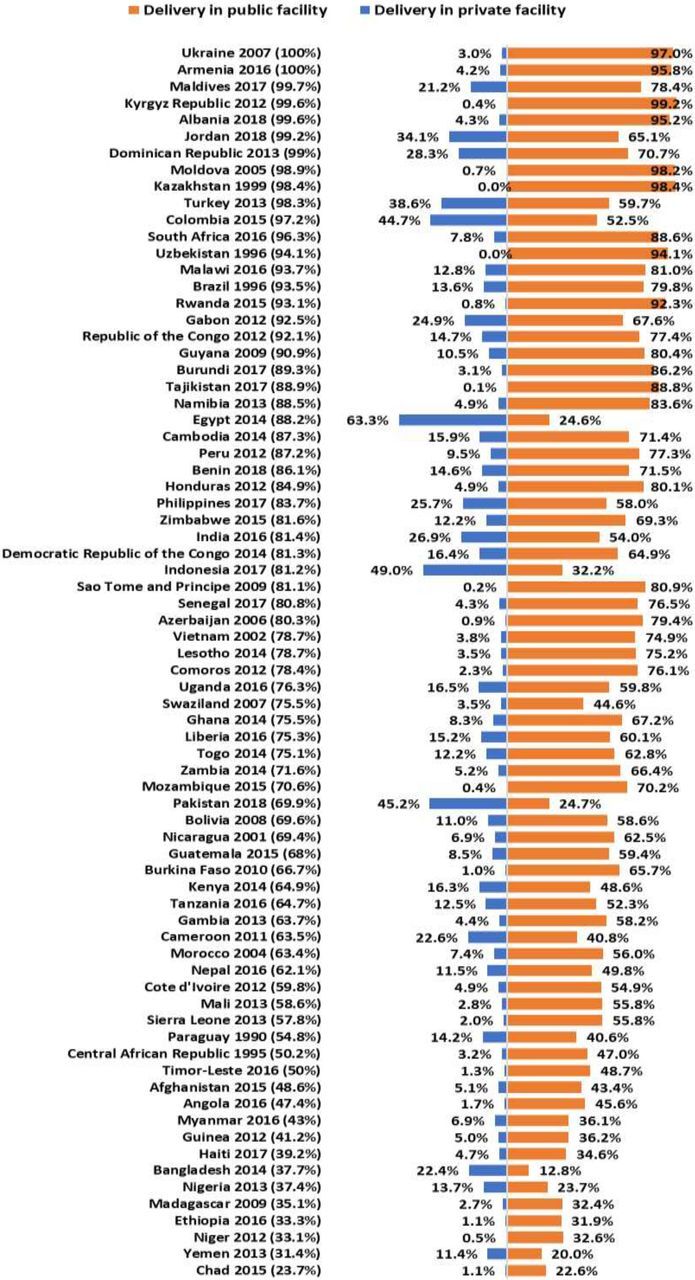
Geographical Variations in the Utilization of Institutional Delivery Services in Low- and Middle-Income Countries During Latest Demographic and Health Survey Rounds^a^ ^a^ Country and year listed indicate the latest survey year of the respective country. Percentage listed is the country's overall institutional delivery service rate during the latest survey.

Our results show that the coverage of institutional delivery services varied between study countries (Figure 1). During 1990–2018, 19 of 74 countries reported that more than 90% of all deliveries were conducted at health facilities, with Armenia and Ukraine having universal coverage of institutional delivery services. In contrast, 13 countries had <50% coverage of institutional delivery services, with the lowest in Chad (23.7%) followed by Yemen (31.4%) and Niger (33.1%). Among all live births, the place of delivery (i.e., public or private health facilities) also varied across countries. In 52 of 74 countries, more than 50% of all live births took place in public health facilities. In comparison, 71 of 72 countries reported less than 50% of deliveries in private health facilities. The rate of public facility-based delivery was highest in the Kyrgyz Republic (99.2%) and lowest in Bangladesh (12.8%). On the other hand, Egypt had the highest rate of private facility–based deliveries (63.3%), whereas Tajikistan had the lowest (0.1%). The rate of delivery in public health facilities was greater than delivery in private health facilities in all countries, except Bangladesh, Egypt, Indonesia, and Pakistan.

### Trends in the Utilization of Institutional Delivery Services

**FIGURE 2:**
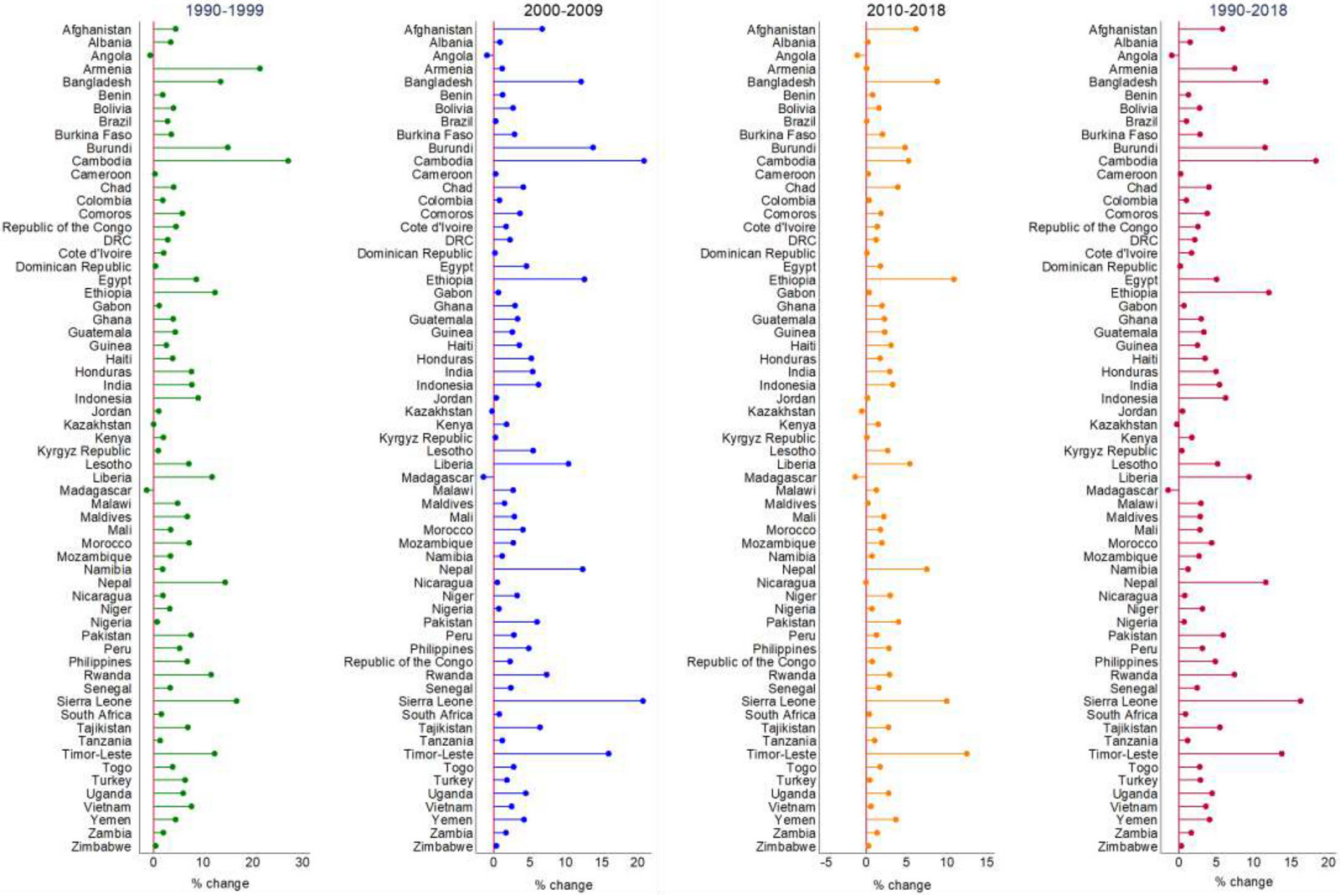
Change Rates of Institutional Delivery Services in Low- and Middle-Income Countries

**FIGURE 3:**
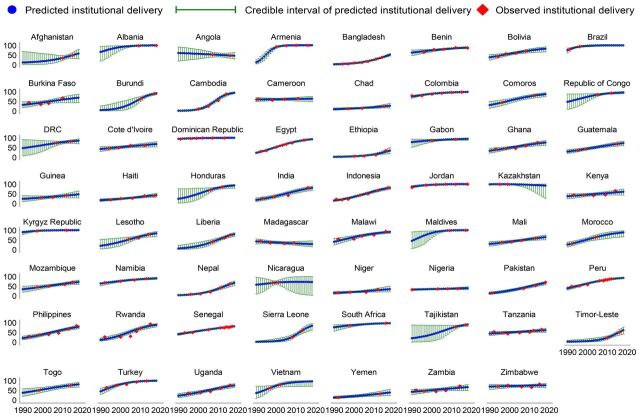
Trends in the Utilization of Institutional Delivery Services in Low- and Middle-Income Countries

During 1990–2018, the utilization of institutional delivery services increased in 60 of 63 study countries (Figure 2). The progress in the utilization of institutional delivery services varied across countries. The highest increase in the utilization of institutional delivery services was observed in Cambodia (an 18.3% annual increase from 0.6% in 1990 to 94.0% in 2018) followed by Sierra Leone (16.2%) and Timor-Leste (13.7%). At the same time, utilization decreased in Angola (−0.9%), Kazakhstan (−0.3%), and Madagascar (−1.4%). The increase in the utilization of institutional delivery services steadily decreased after 1990–1999 in most LMICs (Figure 2). Based on this trend, 31 of 63 countries were estimated to have <80% utilization of institutional delivery services in 2018, with the highest in Armenia (100%, 95% CrI 100%–100%) and lowest in Chad (26.1%, 95% CrI 15.3%–38.7%) (Figure 3).

During 1990–2018, the utilization of institutional delivery services increased in 60 of 63 countries, but the progress varied across countries.

Trends in the utilization of institutional delivery services varied across wealth, residence, education, and age of mother over time. From 1990 to 2018, the utilization of institutional delivery services in the lowest income group increased in 90.3% of countries (56 of 62 countries), with the highest in Cambodia (27.7% increase). In comparison, utilization declined in 9.7% of countries (6 of 62 countries), with the largest decline being −6.1% in Nigeria. Over 90.3% of countries (56 of 62 countries) reported increasing utilization of institutional delivery services by the highest income group, with the highest increase seen in Sierra Leone (14.4%), and 9.7% of countries (6 of 62 countries) showed a decline in the utilization of institutional delivery services with the largest decline in Angola (−0.6%) (Supplement, Table S3). If this trend continues, Nigeria (4.7%, 95% CrI 1.9%–9.6%) and Yemen (47.0%, 95% CrI 0.0%–95.8%) are estimated to have the lowest utilization of institutional delivery services in the lowest and highest income groups, respectively (Supplement, Table S4).

During the same time in rural areas, the utilization of institutional delivery services increased in 93.7% of countries (59 of 63 countries), with the highest increase by 20.5% in Cambodia, and 6.3% of countries (4 of 63 countries) reported a decline in utilization, with the largest decline observed in Angola (−2.1%). In contrast, in urban areas, 95.2% of countries (60 of 63 countries) experienced an increasing rate of institutional delivery services, with the highest increase in Cambodia (14.5%), and 4.8% of countries (3 of 63 countries) showed a decline in the utilization of institutional delivery services, with the largest decline in Angola by −1.4% (Supplement, Table S5). Similar to trends related to wealth and place of residence, the utilization of institutional delivery services also varied over time across women's education (Supplement, Tables S7 and S8) and age (Supplement, Tables S9 and S10).

### Changes in Inequalities in the Utilization of Institutional Delivery Services

**FIGURE 4:**
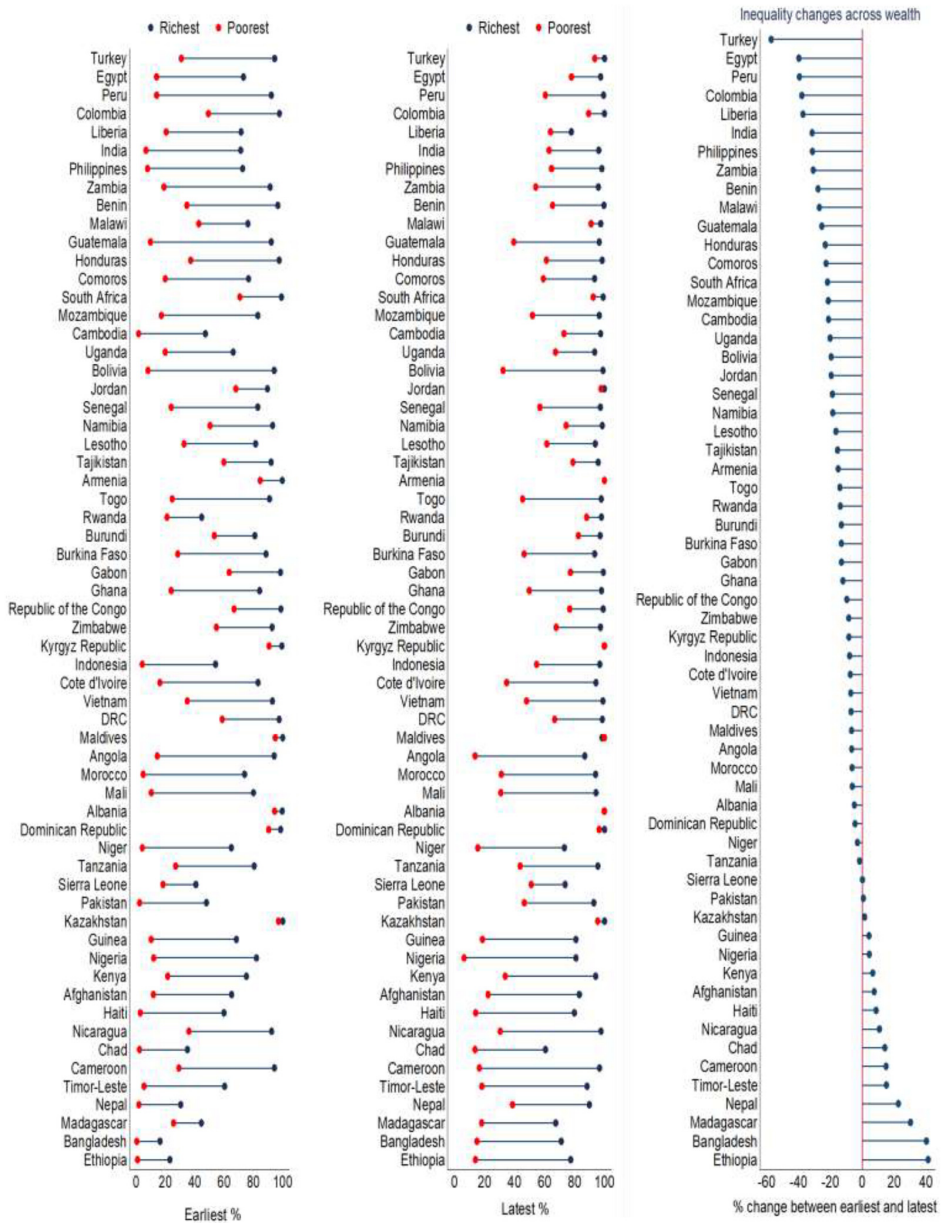
Changes of Inequalities in the Utilization of Institutional Delivery Services Between Earliest and Latest Time Points in Low- and Middle-Income Countries by Wealth Quintiles

Among 60 countries, inequalities in the utilization of institutional delivery services increased in relation to wealth, place of residence, age, and education. Wealth-related inequalities widened in 16 countries during the latest DHS round compared with the earliest, with the highest increase of 41.4% occurring in Ethiopia (earliest round: poorest 0.8%, richest 22.9%; latest round: poorest 13.9%, richest 77.5%) (Figure 4). In terms of place of residence, 10 of 63 countries experienced a growing gap in this inequality, with the highest increase of 29.4% occurring in Ethiopia (earliest round: rural 1.8%, urban 32.5%; latest round: rural 26.3%, urban 86.3%) (Supplement, Figure S1). Among these countries, a widening in the inequality of institutional delivery service utilization was seen in 10 countries in terms of education, with the highest increase of 20.2% in Madagascar (Supplement, Figure S2), and in 36 countries in terms of age, with the highest increase of 13.9% in Burundi (Supplement, Figure S3). In some countries, utilization of institutional delivery servicesincreased among the advantaged groups and decreased among the disadvantaged groups during the latest round of surveys (Supplement, Figures S1–S3). Relative inequalities in the utilization of institutional delivery services also changed during the earliest and latest DHS rounds across wealth, place of residence, education, and age of women (Supplement, Tables S11–S14).

In many study countries, inequalities in the utilization of institutional delivery services increased in relation to wealth, place of residence, age, and education.

### Changes in Institutional Delivery Between Public and Private Facilities

**FIGURE 5:**
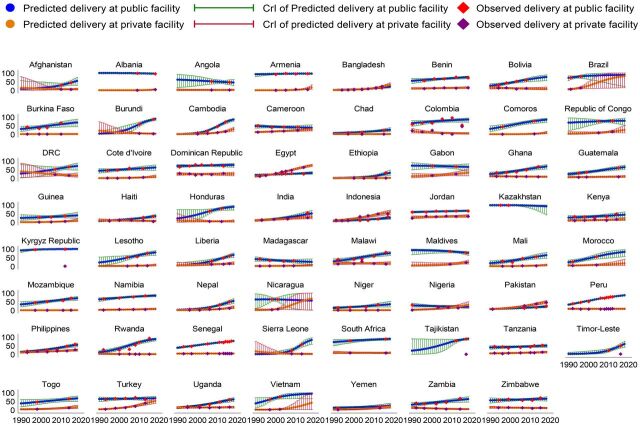
Trends in the Utilization of Delivery Services Facilitated by Public and Private Sectors in Low- and Middle-Income Countries

We explored the variations in the utilization of institutional delivery services by the type of facilities (i.e., public and private health facilities) to understand the differences in service provision (Supplement, Table S15 and Figure 5). Although an increase in the utilization of institutional delivery services was observed, this increase was common across public facilities in 54 countries and private facilities in 43 countries. During 1990–2018, the highest increase in the utilization of institutional delivery services was observed in Sierra Leone (16.8%) in public facilities and Albania (30.2%) in private facilities. During the same period, the utilization of institutional delivery services decreased in some countries in both public and private facilities, with the largest declines in Madagascar (−1.7%) in public facilities and Sierra Leone (−8.1%) in private facilities.

Significant disparities exist in the utilization of delivery services in public and private facilities between countries and across wealth quintiles, residence, education, and age of women within countries. In most of the countries, the delivery in both public and private facilities was mostly dominated by the richest rather than the poorest women (Supplement, Tables S16 and S17). However, these gaps across residence, education, and age are minimal in most countries. (Supplement, Tables S18–S23).

Significant disparities exist in the utilization of delivery services in public and private facilities between countries and across specific populations within countries.

Change rates in the utilization of delivery services in public and private facilities varied between countries, between periods within countries, and between countries and periods across wealth quintiles, residence, education, and age. In public facilities, the increase in the utilization of delivery services was highest in Cambodia (27.9%) among the poorest, and in Sierra Leone (17.2%) among the richest (Supplement, Table S24). Whereas, Cambodia (24.0%) and Albania (33.6%) had the highest increase in the utilization of delivery services in private facilities among the poorest and the richest groups, respectively (Supplement, Table S25). Variations in the utilization of delivery services were also apparent across the place of residence, education, and age in both public and private facilities (Supplement, Tables S26–S31).

## DISCUSSION

During the latest DHS round, the utilization of institutional delivery services varied substantially across countries and over time. The utilization across public and private health facilities was not uniform across countries. Among study LMICs, 16 countries had ≥80% utilization of delivery services in public facilities, whereas no countries had ≥80% utilization of this service in private facilities during the latest DHS rounds. Trend analysis showed a sustained increase in the utilization of institutional delivery services in most countries. Our findings showed a significant influence of wealth quintile, place of residence, and education of women in the utilization of institutional delivery services. In many countries, the service utilization gaps observed in the earliest DHS rounds were found to be persistent and widened further in the latest DHS rounds.

Geographical variations in the utilization of institutional delivery services were expectedly common and were in agreement with previous studies.^18^ During the latest DHS rounds, in nearly 20% of countries included in our analysis, less than half of deliveries took place at a health facility. This finding highlights that still more than half of the babies are delivered at home in many countries such as Chad, Yemen, and Niger. Traditional and familial influences, distance to the facility, cost of delivery, perceptions of low quality of care, and fear of discrimination play a key role in inadequate utilization of facility-based delivery.^19^

Our findings on increasing trends in the utilization of institutional delivery services are consistent with previous studies.^12^ However, our results also highlight uneven progress in the utilization of institutional delivery services between countries and across subpopulations within countries. The utilization of institutional delivery services decreased by nearly 1.5 percentage points in Madagascar.

Our results highlight uneven progress in the utilization of institutional delivery services between countries and across subpopulations within countries.

The presence of disparities in the utilization of institutional delivery services across income and education levels is supported by previous research.^14^^,^^20^ We found lower utilization of institutional delivery services among women of the poorest quintile (lowest income group). Similar to previous research, we also found that compared with their counterparts, women from the lowest income group have lower access to private facilities for delivery.^13^^,^^15^^,^^21^ We also identified countries such as Bangladesh where inequality in the utilization of institutional delivery services is further increased. In particular, wealth-based inequalities in the utilization of institutional delivery services widened in 19 countries, while residence- and education-based inequalities grew further in 10 countries each. This finding highlights the need of revisiting strategies and implementing appropriate interventions to reduce the inequalities in the utilization of institutional delivery services across various sociodemographic groups.

Our study demonstrates an increasing trend in the majority of countries toward greater utilization of public health facilities for delivery. The predominant role of public facilities in increased utilization of institutional delivery services was proven in previous studies.^13^ The reasons for using public facilities for delivering births could be multifaceted. The lower delivery cost is reported to greatly influence the use of this service.^22^^,^^23^ Also, increasing the number of health care providers through recruitment and improving the quality of care by training frontline health service providers are the key factors driving the growing rates of delivery in public facilities.^24^^–^^27^ However, compared with public facility-based deliveries, the rate of deliveries in private facilities was greater in Egypt, Indonesia, Pakistan, and Bangladesh by 38.6, 29.4, 20.5, and 9.6 percentage points respectively. The higher rate of deliveries in private facilities could be due to better quality of services, shorter wait time, higher availability of health care providers, greater privacy, and the visualization of social status.^28^ Affordability and availability of private services are also increased due to the growth of gross domestic product per capita in these countries.^29^ In general, an increase in awareness about the benefit of facility-based delivery might be the key to the increased utilization of institutional delivery services.^12^ This may further result in the reduction of maternal and newborn mortality. However, we acknowledge that merely moving births to health facilities does not eliminate maternal and child mortality.

Our analysis has shown that most LMICs have reported remarkable improvement in the utilization of institutional delivery services. For example, the LAC countries showed the highest utilization of institutional delivery services. Innovative strategies have helped reduce financial barriers to access maternal health care in LAC countries; these include national health insurance (Brazil, Chile, Colombia, Jamaica, Mexico, and Peru), free health insurance scheme for lower-income families (Bolivia, Mexico, and Peru), incorporating UHC as a constitutional right (Brazil and Chile), and public-private partnerships (Colombia).^30^ Reducing the gap in provisioning institutional delivery services for a particular demographic group, such as the one for indigenous and African origin women, was also considered there.^30^ Some vertical approaches can also be attributed to the growing rates of institutional delivery services utilization in Asian countries. For example, conditional cash incentives in India^31^ and demand-side financing in Bangladesh^32^ are linked with higher utilization of institutional delivery services.

Higher rates of home delivery assisted by a traditional birth attendant are common in many settings. Restricting the services from a traditional birth attendant backed up by hospital readiness increased facility-based births from <30% during the start of MDG era to the current rate of >90% rate in Malawi and Rwanda.^33^^–^^36^ In contrast, countries with slower progress or decreasing trends of utilizing institutional delivery services such as Angola, Kazakhstan, and Madagascar have shown higher inequality in the utilization of institutional delivery services. Distance to health facilities, lower educational level, and rural residence were the major determinants of poor utilization of this service in sub-Saharan African countries.^7^ Moreover, the current coronavirus disease (COVID-19) pandemic situation can aggravate the poor utilization of institutional delivery services because people may not be accessing health facilities as they would have before COVID.

The major strength of this study is the use of population-based nationally representative samples covering both rural and urban areas of 74 LMICs and the identification of population subgroups within LMICs. Analysis at the subpopulation level is particularly helpful to design interventions for target populations.

### Limitations

The use of the same standard methodology across countries allows cross-country comparison of the estimates. However, older and fewer data points created wider credible intervals of the estimates in some countries (e.g., Kazakhstan). Credible intervals could be smaller for countries with many data points (e.g., Bangladesh). Estimates drawn from authentic representative data collected from multiple sources may better predict the indicators with lower uncertainty. Moreover, the DHS data are mostly self-reported and hence are prone to recall bias. However, the DHS has followed a standard methodology and questionnaire for more than 3 decades to provide population-based data that are comparable and representative not only at the national level but also subnational and subpopulation levels.

## CONCLUSIONS

Although the utilization of institutional delivery services varied substantially across LMICs, the utilization of health facilities for delivery overall increased in most of the countries between 1990 and 2018. However, this increase was not uniform across countries and sociodemographic subpopulations (e.g., poorest and richest, and rural and urban) within countries. Unfortunately, inequalities in the utilization of institutional delivery services are widening in some countries. These findings warrant the development of appropriate and tailored interventions covering the disadvantaged and marginalized populations identified in this study to achieve the global target of “leaving no one behind” for the utilization of institutional delivery services by 2030.

## Supplementary Material

20-00533-Hasan-Supplement.pdf

## Notes

**Peer Reviewed**

**First published online:** February 10, 2021.
