# Supplementary material for: Levels, Trends, and Inequalities in Using Institutional Delivery Services in Low- and Middle-Income Countries: A Stratified Analysis by Facility Type
Source: Glob Health Sci Pract. 2021 Mar 31;9(1):78–88. doi: 10.9745/GHSP-D-20-00533 (PMC8087431; doi:10.9745/GHSP-D-20-00533)
Supplement: 20-00533-Hasan-Supplement.pdf [file 20-00533-Hasan-Supplement.pdf]

Supplement to: Hasan MM, Magalhaes RJS, Fatima Y, Ahmed S, Mamun AA. Levels, trends and inequalities in using institutional delivery services in low- and middle-income countries: a stratified analysis by facility type. *Glob Health Sci Pract*. 2021;9(1). https://doi.org/10.9745/GHSP-D-20-00533

Supplement

Modeling

We applied Bayesian linear regression models using normally distributed non-informative priors to calculate the estimates of institutional delivery services during 1990-2018. This technique was previously used to examine trends for health indicators[1]. For analysis, we first draw the prevalence estimates in proportions for institutional delivery services for all survey years of each study country. This provided us the prevalence rate of institutional delivery services for the respective survey years of the country. This rate estimates were missing for the years when the DHS was not conducted in the study countries. We then prepared a dataset by using the continuous rate of institutional delivery services of each country. For example, the DHS in Bangladesh provided prevalence rate of institutional delivery services for 1994, 1997, 2000, 2004, 2007, 2011 and 2014. We prepared a dataset for Bangladesh by using the rate of these survey years. We performed a logit transformation of all proportions. All the calculations were done after this transformation and then transformed back to probabilities to ensure that the predicted probabilities lie between 0 and 1. We considered "survey year" as time as a covariate in each model.

We obtained 30000 samples from the posterior distribution by applying a Markov Chain Monte Carlo (MCMC) algorithm with two chains. MCMC is a general sampling method to draw values/samples from parameter distributions which are then used to estimate the posterior distribution of parameters given the data. In our data, no countries had data on the utilisation of institutional delivery services for all the survey years (time points). Therefore, all the countries had time points between 1990-2018 with missing rate of utilising institutional delivery services. Therefore, we used a priori knowledge to generate/assign values for the missing rate of institutional delivery services for the period 1990-2018. For trend analysis, we dropped the countries that do not have data for at least two time points during this period.

We used both trace plots and Gelman-Rubin diagnostic statistics to check parameter convergence by using the value of potential scale reduction factor (PSRF)[2]. We considered that the convergence was achieved if the value of PSRF was close to 1, and a value greater than 1.10 was considered as convergence failure. We used *rjags* package[3] in the R programming language with JAGS, a BUGS language, MCMC library for generating the estimates. For each model, the first 5000 iterations were discarded as burn-in and the number of iterations were increased until the output was diagnosed as convergent. During checking the convergence, trace plots were generated each time to verify the mixing of iterations, and the iteration chains were always past in burn-in phase. These posterior predictive distributions were used to obtain estimates along with 95% credible intervals (CrI). We conducted subgroup analysis to examine the trends across subgroups such as wealth quintiles, place of residence, education, and age of women/mother to understand the sociodemographic groups who are lagging.

Model equation

We defined our indicators y_i as the logit transformed value of the proportion of the indicator and assumed the distribution as follows:

$$y_i \sim N(\mu_i, \sigma_i^2) \quad \ldots\ldots.. \text{(i)}$$

Here, μ_i is the conditional mean of the indicator and has the following linear form:

$$\mu_i = \beta_0 + \beta_1 x_i \ldots\ldots\ldots \quad \text{(ii)}$$

where, x_i is the time, β_0 is the intercept and β_1 is the regression coefficient. The prior distribution for β_0 and β_1 was assumed as follows:

$$\beta_0 \sim \text{Normal}(0, 0.0001)$$

$$\beta_1 \sim \text{Normal}(0, 0.0001)$$

and

$$\sigma^2 \sim \text{Normal}(0.0001, 100)$$

We fitted the model separately for each country at overall level. We repeated the procedure to examine the trends for each subgroups of each country.

Handling missing values

For trend analysis, we omitted the countries with missing values for the indicator for more than one time point.

Cross-validation of estimates

We compared the estimates drawn from the Bayesian linear regression model (predicted estimates) with those drawn from original micro-data (original estimates). We calculated the absolute differences in the estimates drawn from regression models and from original data for the indicator for several countries for the most recent time points. We then checked the differences between them to validate the results. This procedure depicts that the difference between original and estimated coverage of institutional delivery services utilization were very little, mostly differed by -2 to 4.5 percentage points in majority of the countries. See **Table S2** for details.

Supplement to: Hasan MM, Magalhaes RJS, Fatima Y, Ahmed S, Mamun AA. Levels, trends and inequalities in using institutional delivery services in low- and middle-income countries: a stratified analysis by facility type. *Glob Health Sci Pract*. 2021;9(1). https://doi.org/10.9745/GHSP-D-20-00533

Supplementary Tables

Table S1: Survey year and sample size for institutional delivery services across country

Country	Survey year	Number of live births	Delivery at institute
**Overall**	**1990-2018**	**1,538,486**	**882,953**
South and Southeast Asia			
Afghanistan	2010	12,979	5,113
Afghanistan	2015	32,204	14,645
Bangladesh	1994	3,847	146
Bangladesh	1997	3,582	185
Bangladesh	2000	4,118	456
Bangladesh	2004	4,084	557
Bangladesh	2007	3,632	721
Bangladesh	2011	4,952	1,556
Bangladesh	2014	4,719	1,850
Cambodia	2000	4,999	414
Cambodia	2005	5,005	1,031
Cambodia	2010	8,166	4,351
Cambodia	2014	4,379	3,779
India	1993	36,650	10,355
India	1999	32,850	11,465
India	2006	30,334	14,266
India	2016	153,003	119,378
Indonesia	1991	9,391	2,257
Indonesia	1994	10,749	2,011
Indonesia	1997	10,541	2,339
Indonesia	2003	9,888	3,880
Indonesia	2007	11,105	4,700
Indonesia	2012	10,838	6,294
Indonesia	2017	10,564	8,008
Maldives	2009	2,415	2,357
Maldives	2017	1,687	1,684
Myanmar	2016	2,854	1,113
Nepal	1996	4,378	345
Nepal	2001	3,975	413
Nepal	2006	3,340	633
Nepal	2011	3,047	1,298
Nepal	2016	2,829	1,773
Pakistan	1991	3,940	698
Pakistan	2007	5,418	2,034
Pakistan	2013	6,829	3,673
Pakistan	2018	7,460	5,091
Philippines	1993	5,574	1,485
Philippines	1998	4,847	1,464
Philippines	2003	4,232	1,551
Philippines	2008	3,938	1,688
Philippines	2013	4,220	2,676
Philippines	2017	5,892	4,671
Timor-Leste	2010	9,792	1,879
Timor-Leste	2016	4,355	2,182
Vietnam	1997	1,775	1,169
Vietnam	2002	1,312	1,048
Central Asia			
Kazakhstan	1995	1,690	1,656
Kazakhstan	1999	1,524	1,492
Kyrgyz Republic	1997	2,248	2,174
Kyrgyz Republic	2012	5,490	5,472
Tajikistan	2012	6,352	5,038
Tajikistan	2017	7,414	6,552
Uzbekistan	1996	2,646	2,532
Sub-Saharan Africa			
Angola	2007	664	310
Angola	2016	8,705	3,833
Benin	1996	2,974	1,834
Benin	2001	3,210	2,496
Benin	2006	9,739	7,761
Benin	2012	7,846	6,849

Country	Survey year	Number of live births	Delivery at institute
Benin	2018	8,075	6,918
Burkina Faso	1993	3,414	1,833
Burkina Faso	1999	3,456	1,306
Burkina Faso	2003	6,197	2,564
Burkina Faso	2010	14,970	10,270
Burundi	2010	7,436	4,833
Burundi	2017	7,566	6,767
Cameroon	1991	2,016	1,376
Cameroon	1998	2,286	1,377
Cameroon	2004	4,883	2,959
Cameroon	2011	7,151	4,602
Central African Republic	1995	2,796	1,376
Chad	1997	4,377	730
Chad	2004	3,296	784
Chad	2015	10,504	2,203
Comoros	1996	1,141	488
Comoros	2012	1,926	1,505
Republic of the Congo	2005	3,032	2,619
Republic of the Congo	2012	5,748	4,950
Democratic Republic of the Congo	2007	5,434	3,976
Democratic Republic of the Congo	2014	11,262	8,515
Cote d'Ivoire	1994	3,982	1,878
Cote d'Ivoire	1999	1,254	757
Cote d'Ivoire	2005	1,960	1,116
Cote d'Ivoire	2012	4,750	2,696
Ethiopia	2000	6,315	624
Ethiopia	2005	5,669	626
Ethiopia	2011	6,624	997
Ethiopia	2016	6,126	2,359
Gabon	2000	2,651	2,186
Gabon	2012	3,818	3,200
Gambia	2013	5,110	3,002
Ghana	1993	2,184	928
Ghana	1998	1,948	789
Ghana	2003	2,288	945
Ghana	2008	1,813	1,008
Ghana	2014	3,606	2,598
Guinea	1999	3,399	1,042
Guinea	2005	3,927	1,202
Guinea	2012	4,214	1,691
Kenya	1993	3,636	1,537
Kenya	1998	3,478	1,386
Kenya	2003	3,601	1,482
Kenya	2009	3,722	1,636
Kenya	2014	12,281	7,036
Lesotho	2004	2,263	1,216
Lesotho	2009	2,461	1,409
Lesotho	2014	1,926	1,471
Liberia	2007	3,424	1,363
Liberia	2013	4,562	2,573
Liberia	2016	1,608	1,177
Madagascar	1992	3,220	1,470
Madagascar	1997	3,639	1,240
Madagascar	2004	3,185	1,192
Madagascar	2009	7,355	2,655
Malawi	1992	2,814	1,752
Malawi	2000	7,510	4,191
Malawi	2004	6,717	4,694
Malawi	2010	19,380	14,446
Malawi	2016	10,051	9,493
Mali	1996	6,010	2,192
Mali	2001	7,831	2,851
Mali	2006	8,536	3,976
Mali	2013	5,925	3,531
Mozambique	1997	4,067	2,075
Mozambique	2003	6,067	3,376
Mozambique	2011	6,796	4,383
Mozambique	2015	2,805	2,109

Country	Survey year	Number of live births	Delivery at institute
Namibia	1992	2,524	1,674
Namibia	2000	2,454	1,833
Namibia	2007	3,230	2,599
Namibia	2013	3,145	2,716
Niger	1992	4,106	1,278
Niger	1998	4,764	1,140
Niger	2006	5,570	1,528
Niger	2012	7,479	2,923
Nigeria	1990	4,685	1,752
Nigeria	2003	3,632	1,345
Nigeria	2008	16,865	5,478
Nigeria	2013	18,758	7,179
Rwanda	1992	3,276	997
Rwanda	2000	4,652	1,395
Rwanda	2005	5,425	1,685
Rwanda	2008	3,170	1,763
Rwanda	2010	8,816	6,207
Rwanda	2015	4,785	4,476
Sao Tome and Principe	2009	1,187	943
Senegal	1993	3,355	1,596
Senegal	1997	4,389	1,993
Senegal	2005	6,826	4,173
Senegal	2011	7,578	5,022
Senegal	2013	4,142	2,834
Senegal	2014	4,135	2,903
Senegal	2015	4,187	2,917
Senegal	2016	8,191	5,787
Senegal	2017	7,139	5,471
Sierra Leone	2008	3,460	1,002
Sierra Leone	2013	7,051	4,188
South Africa	1998	3,075	2,575
South Africa	2016	2,064	1,991
Swaziland	2007	1,735	1,306
Tanzania	1992	5,090	2,486
Tanzania	1996	4,078	1,960
Tanzania	1999	1,960	851
Tanzania	2005	5,282	2,429
Tanzania	2010	7,904	3,925
Tanzania	2012	4,609	2,575
Tanzania	2016	6,185	3,978
Togo	1998	4,131	1,938
Togo	2014	4,234	3,003
Uganda	1995	4,400	1,884
Uganda	2001	4,305	1,859
Uganda	2006	5,047	2,182
Uganda	2011	4,713	2,884
Uganda	2016	9,139	6,912
Zambia	1992	4,049	2,011
Zambia	1996	4,512	1,882
Zambia	2002	4,270	1,728
Zambia	2007	3,962	1,961
Zambia	2014	7,854	5,659
Zimbabwe	1994	2,423	1,655
Zimbabwe	1999	2,138	1,609
Zimbabwe	2006	3,184	2,093
Zimbabwe	2011	3,547	2,332
Zimbabwe	2015	3,522	2,973
North Africa-West Asia-Europe			
Albania	2009	877	853
Albania	2018	1,618	1,609
Armenia	2000	950	876
Armenia	2005	895	883
Armenia	2010	1,468	1,465
Armenia	2016	1,028	1,028
Azerbaijan	2006	1,394	1,143
Egypt	1992	5,036	1,498
Egypt	1995	7,127	2,303
Egypt	2000	7,056	3,706

Country	Survey year	Number of live births	Delivery at institute
Egypt	2003	4,053	2,286
Egypt	2005	8,369	5,451
Egypt	2008	6,872	4,991
Egypt	2014	9,956	8,845
Jordan	1990	4,953	4,013
Jordan	1997	3,882	3,636
Jordan	2002	3,691	3,566
Jordan	2007	6,246	6,165
Jordan	2012	6,117	6,071
Jordan	2018	6,305	6,260
Moldova	2005	1,000	991
Morocco	1992	3,052	915
Morocco	2004	3,593	2,192
Turkey	1993	2,217	1,440
Turkey	1998	2,158	1,602
Turkey	2003	2,612	1,991
Turkey	2008	2,332	2,098
Turkey	2013	2,147	2,094
Ukraine	2007	714	714
Yemen	1992	4,300	719
Yemen	2013	9,655	3,290
Latin America & Caribbean			
Bolivia	1994	3,628	1,507
Bolivia	1998	4,289	2,272
Bolivia	2003	5,905	3,757
Bolivia	2008	5,197	3,705
Brazil	1991	1,866	1,522
Brazil	1996	2,999	2,761
Colombia	1990	2,286	1,846
Colombia	1995	3,093	2,426
Colombia	2000	2,521	2,184
Colombia	2005	7,856	7,039
Colombia	2010	14,490	13,218
Colombia	2015	6,422	6,101
Dominican Republic	1991	2,601	2,406
Dominican Republic	1996	2,757	2,616
Dominican Republic	1999	324	311
Dominican Republic	2002	6,929	6,765
Dominican Republic	2007	6,404	6,244
Dominican Republic	2013	2,211	2,167
Guatemala	1995	6,110	1,487
Guatemala	1999	2,976	855
Guatemala	2015	7,437	5,079
Guyana	2009	1,377	1,132
Haiti	1995	2,088	409
Haiti	2000	4,056	803
Haiti	2006	3,676	902
Haiti	2012	4,473	1,599
Haiti	2017	3,854	1,439
Honduras	2006	6,406	4,037
Honduras	2012	6,700	5,392
Nicaragua	1998	4,853	2,980
Nicaragua	2001	4,173	2,658
Paraguay	1990	2,570	1,262
Peru	1992	5,360	2,406
Peru	1996	10,019	4,453
Peru	2000	7,036	3,673
Peru	2006	9,319	6,966
Peru	2008	9,319	6,966
Peru	2009	5,938	4,767
Peru	2010	9,203	7,309
Peru	2011	5,375	4,418
Peru	2012	5,665	4,794

Table S2: Cross-validation of estimates drawn from original micro data and regression models for some countries during the most recent survey years in low- and middle-income countries

Country	Survey year	Utilization of institutional delivery services		
		**Original estimates**	**Predicted estimates**	**Difference between original and predicted estimates**
Afghanistan	2015	48.6	48.7	-0.1
Albania	2018	99.6	99.5	0.0
Angola	2016	47.4	47.2	0.1
Armenia	2016	100.0	100.0	0.0
Bangladesh	2014	37.7	37.3	0.3
Benin	2012	88.2	87.8	0.4
Bolivia	2008	69.6	69.5	0.0
Brazil	1996	93.5	93.3	0.1
Burkina Faso	2010	66.7	58.0	8.6
Burundi	2017	89.3	89.0	0.3
Cambodia	2014	87.3	83.6	3.7
Cameroon	2011	63.5	61.5	1.9
Chad	2015	23.7	23.3	0.4
Colombia	2015	97.2	97.0	0.2
Comoros	2012	78.4	78.0	0.4
Republic of the Congo	2012	92.1	92.0	0.1
Democratic Republic of the Congo	2014	81.3	81.0	0.3
Cote d'Ivoire	2012	59.8	62.1	-2.3
Dominican Republic	2013	99.0	99.2	-0.2
Egypt	2014	88.2	87.2	1.0
Ethiopia	2016	33.3	27.1	6.2
Gabon	2012	92.5	92.4	0.1
Ghana	2014	75.5	70.0	5.5
Guatemala	2015	68.0	67.8	0.1
Guinea	2012	41.2	40.1	1.1
Haiti	2017	39.2	40.9	-1.7
Honduras	2012	84.9	84.4	0.5
India	2016	81.4	76.0	5.5
Indonesia	2012	67.2	64.2	3.0
Jordan	2012	98.8	99.1	-0.4
Kazakhstan	1999	98.4	98.3	0.1
Kenya	2014	64.9	55.9	9.0
Kyrgyz Republic	2012	99.6	99.6	0.0
Lesotho	2014	78.7	76.5	2.2
Liberia	2016	75.3	73.1	2.2
Madagascar	2009	35.1	32.0	3.1
Malawi	2016	93.7	89.4	4.4
Maldives	2017	99.7	99.6	0.0
Mali	2013	58.6	57.5	1.1
Morocco	2004	63.4	63.0	0.4
Mozambique	2015	70.6	67.3	3.2
Namibia	2013	88.5	87.6	0.9
Nepal	2016	62.1	59.0	3.1
Nicaragua	2001	69.4	69.0	0.4
Niger	2012	33.1	28.7	4.4
Nigeria	2013	37.4	37.6	-0.2
Pakistan	2018	69.9	66.3	3.6
Peru	2012	87.2	86.0	1.2
Philippines	2017	83.7	76.1	7.7
Rwanda	2015	93.1	83.1	10.0
Senegal	2017	80.8	79.3	1.5
Sierra Leone	2013	57.8	57.6	0.2
South Africa	2016	96.3	96.2	0.2
Tajikistan	2017	88.9	88.5	0.4
Tanzania	2016	64.7	59.2	5.6
Timor-Leste	2016	50.0	49.9	0.0
Togo	2014	75.1	75.2	-0.1
Turkey	2003	80.3	81.0	-0.7
Uganda	2016	76.3	71.1	5.2
Vietnam	2002	78.7	78.4	0.2
Yemen	2013	31.4	31.7	-0.3
Zambia	2014	71.6	62.3	9.3
Zimbabwe	2015	81.6	74.6	6.9

Supplement to: Hasan MM, Magalhaes RJS, Fatima Y, Ahmed S, Mamun AA. Levels, trends and inequalities in using institutional delivery services in low- and middle-income countries: a stratified analysis by facility type. *Glob Health Sci Pract*. 2021;9(1). https://doi.org/10.9745/GHSP-D-20-00533

Table S3: Change rates in institutional delivery services in low- and middle-income countries by wealth quintiles

	Poorest				Richest			
**Country**	**1990-1999**	**2000-2009**	**2010-2018**	**1990-2018**	**1990-1999**	**2000-2009**	**2010-2018**	**1990-2018**
Afghanistan	4.9	10.1	12.4	9.1	9.6	10.0	3.9	8.1
Albania	22.9	8.0	0.5	10.7	20.2	1.8	0.0	7.3
Angola	-1.7	-1.1	-0.2	-1.0	-0.2	-0.4	-1.2	-0.6
Armenia	36.6	2.6	0.0	13.1	0.1	0.0	0.0	0.0
Bangladesh	18.7	18.4	15.7	17.7	10.2	7.2	3.8	7.2
Benin	3.3	2.5	1.8	2.6	0.4	0.1	0.1	0.2
Bolivia	9.8	8.4	5.3	8.0	0.6	0.2	0.0	0.3
Burkina Faso	3.0	3.0	2.6	2.9	0.7	0.4	0.3	0.5
Burundi	12.5	12.4	5.8	10.5	21.4	14.9	2.5	13.3
Cambodia	36.0	33.1	11.5	27.7	20.3	8.1	1.0	10.1
Cameroon	-2.2	-2.3	-2.2	-2.2	0.3	0.2	0.1	0.2
Chad	10.6	11.3	11.1	11.0	3.9	3.2	2.4	3.2
Colombia	4.6	2.5	1.2	2.8	0.4	0.1	0.0	0.2
Comoros	8.7	6.7	3.9	6.5	2.5	1.2	0.5	1.4
Republic of the Congo	3.2	2.6	1.4	2.4	0.4	0.1	0.0	0.2
Democratic Republic of the Congo	2.4	2.3	1.6	2.1	0.9	0.3	0.1	0.5
Cote d'Ivoire	5.1	4.7	3.8	4.6	1.0	0.6	0.3	0.6
Dominican Republic	0.5	0.3	0.2	0.3	4.3	0.1	0.0	1.4
Egypt	13.5	9.3	3.9	9.1	3.8	1.2	0.3	1.8
Ethiopia	17.8	18.7	18.2	18.3	12.7	10.1	5.2	9.5
Gabon	2.5	1.8	1.1	1.8	0.2	0.1	0.0	0.1
Ghana	3.7	3.6	3.1	3.5	1.9	0.7	0.3	1.0
Guatemala	8.8	8.1	6.3	7.8	0.4	0.3	0.2	0.3
Guinea	4.4	4.7	4.6	4.5	2.1	1.5	0.9	1.5
Haiti	8.1	7.9	7.5	7.8	1.9	1.4	1.0	1.4
Honduras	10.1	10.3	5.0	8.7	0.8	0.3	0.1	0.4
India	12.2	10.6	6.9	10.0	3.2	1.4	0.6	1.8
Indonesia	15.5	13.9	9.4	13.1	7.2	2.9	0.8	3.7
Jordan	1.7	0.7	0.2	0.9	0.6	0.1	0.0	0.2
Kazakhstan	-0.3	-1.4	-2.5	-1.3	0.0	0.0	0.0	0.0
Kenya	1.6	1.7	1.7	1.7	1.9	1.1	0.6	1.2
Kyrgyz Republic	4.3	0.5	0.0	1.6	0.5	0.0	0.0	0.2
Lesotho	8.0	7.8	5.0	7.0	4.7	2.1	0.7	2.6
Liberia	17.4	17.1	10.1	15.1	1.7	1.4	0.9	1.3
Madagascar	-3.0	-3.0	-2.6	-2.9	4.5	3.2	1.9	3.3
Malawi	6.3	3.8	1.8	4.1	2.2	1.0	0.4	1.2
Maldives	44.5	19.4	0.4	22.2	0.0	0.0	-0.4	-0.1
Mali	6.7	6.2	4.9	6.0	1.9	1.0	0.5	1.1
Morocco	16.8	12.2	4.7	11.5	3.0	0.8	0.2	1.4
Mozambique	6.9	6.0	4.5	5.8	1.7	0.8	0.3	1.0
Namibia	2.2	1.7	1.3	1.8	0.5	0.2	0.1	0.3
Nepal	17.2	16.8	13.4	15.9	12.2	6.7	2.2	7.2
Nicaragua	-4.4	-3.3	-1.0	-3.0	11.5	0.4	0.0	3.9
Niger	8.3	9.1	8.8	8.8	1.0	0.9	0.6	0.9
Nigeria	-6.0	-6.5	-5.8	-6.1	0.1	-0.1	-0.2	-0.1
Pakistan	13.0	11.9	9.1	11.4	4.2	2.4	1.1	2.6
Peru	11.5	8.8	4.8	8.5	2.1	0.2	0.0	0.8
Philippines	11.3	10.1	7.2	9.6	3.7	1.5	0.5	1.9
Rwanda	12.0	8.8	4.1	8.4	9.4	4.0	1.1	4.9
Senegal	5.0	4.4	3.5	4.3	1.3	0.6	0.3	0.7
Sierra Leone	17.7	24.3	12.7	18.7	16.8	18.7	6.3	14.4
South Africa	3.3	1.8	0.8	2.0	0.0	0.0	0.0	0.0
Tajikistan	8.5	10.2	5.9	8.4	4.7	3.1	0.9	3.0
Tanzania	2.7	2.5	2.2	2.5	1.7	0.9	0.5	1.0
Timor-Leste	10.0	18.1	20.0	16.0	16.3	16.7	5.4	13.2
Togo	4.2	3.9	3.2	3.8	1.3	0.5	0.2	0.7
Turkey	10.5	4.8	1.3	5.7	2.6	0.1	0.0	0.9
Uganda	8.7	7.2	4.7	7.0	3.4	1.7	0.8	2.0
Vietnam	6.9	4.8	1.9	4.7	8.4	0.3	0.0	2.8
Yemen	-0.4	-3.7	27.5	5.9	0.0	0.3	-2.1	-0.5
Zambia	7.6	6.6	4.7	6.3	0.5	0.3	0.2	0.3
Zimbabwe	0.2	0.2	0.1	0.2	0.2	0.2	0.1	0.2

Supplement to: Hasan MM, Magalhaes RJS, Fatima Y, Ahmed S, Mamun AA. Levels, trends and inequalities in using institutional delivery services in low- and middle-income countries: a stratified analysis by facility type. *Glob Health Sci Pract*. 2021;9(1). https://doi.org/10.9745/GHSP-D-20-00533

Table S4: Trends in the utilization (%, 95% credible intervals) of institutional delivery services in low- and middle-income countries by wealth quintiles

	Poorest				Richest			
**Country**	**1990**	**2000**	**2010**	**2018**	**1990**	**2000**	**2010**	**2018**
Afghanistan	2.6 (0-21.1)	4.3 (0.3-20.7)	12 (6.5-20.4)	32.3 (14.2-55.9)	9.3 (0.1-61)	24.7 (3.2-69.3)	65 (51.8-77.4)	89.1 (75.4-96.1)
Albania	4.9 (0.4-22.1)	46 (18.7-75.9)	95.7 (93.2-97.6)	99.7 (99.5-99.8)	12.8 (1-47)	84.7 (60.3-95.9)	99.7 (99.5-99.8)	100 (100-100)
Angola	18.8 (2.3-56.9)	15.9 (5.2-35.4)	14.3 (9.7-19.8)	14.1 (6.7-24.7)	98.2 (92-99.8)	96.6 (91.2-99)	92.2 (88.7-95)	83.9 (73-91.8)
Armenia	2.6 (0.2-10.1)	79.1 (59.3-91.4)	99.9 (99.8-99.9)	100 (100-100)	99 (96.3-99.9)	99.9 (99.8-100)	100 (100-100)	100 (100-100)
Bangladesh	0.2 (0.1-0.4)	1.4 (1.1-1.8)	8.8 (6.6-11.7)	30.8 (20.4-42.8)	10.5 (7.4-14.4)	28.8 (25.2-32.7)	58.4 (53.3-63.2)	79 (72.4-84.5)
Benin	35.5 (20.6-52.3)	49 (38.7-58.7)	62.9 (54.8-70.7)	72.6 (60.4-82.4)	94.1 (90.5-96.5)	97.9 (97.2-98.4)	99.2 (99-99.4)	99.7 (99.5-99.8)
Bolivia	6.7 (3.1-11.4)	17.8 (13.7-22.6)	40.6 (27-55.6)	62.2 (38.9-83.8)	92 (85.4-96.4)	97.8 (96.9-98.4)	99.3 (98.8-99.7)	99.7 (99.3-99.9)
Burkina Faso	20.2 (10-34.9)	27.4 (20.3-35.2)	37 (24.3-52.2)	45.4 (22.9-72)	80.8 (66.9-90.4)	86.9 (81.8-90.7)	90.7 (83.8-95.1)	92.7 (82.1-97.6)
Burundi	4.5 (0.1-28.4)	15.8 (2.7-47.6)	53.5 (39.2-68.7)	84.8 (75-92.1)	2.3 (0.1-14.1)	19.6 (3.4-51)	80.1 (68.9-87.5)	97.7 (95.8-98.9)
Cambodia	0 (0-0.1)	1.4 (1-2.2)	36.5 (30.3-42.1)	91.4 (86.5-94.8)	5.9 (1.7-14.4)	42.9 (30.1-58)	91 (87.8-93.7)	98.7 (97.5-99.5)
Cameroon	31.5 (21.2-43.6)	25.2 (19.9-31.5)	20 (13.4-28.3)	16.8 (8.5-29.6)	89.9 (81.4-95)	92.9 (89.6-95.3)	94.9 (90.7-97.4)	95.8 (90.2-98.6)
Chad	0.7 (0.2-1.8)	2.1 (1.1-3.5)	6.4 (3.5-10.3)	15.6 (6.4-30.7)	26.2 (14.7-40.9)	38.7 (30-47.7)	53.2 (44.2-61.5)	64.3 (50-76.6)
Colombia	41.8 (32.4-53.5)	65.6 (59.9-72)	83.5 (78.4-87.7)	91.6 (86.5-94.8)	95.8 (91-98.2)	99.2 (98.8-99.5)	99.8 (99.7-99.9)	100 (99.9-100)
Comoros	11.8 (4.7-23.2)	27.8 (18.5-38)	53.7 (41-65.7)	73.2 (55.1-86.6)	64.7 (43.6-80.2)	82.1 (74.2-88)	91.9 (87-94.9)	95.7 (91.1-98.2)
Republic of the Congo	41.6 (5.8-87.1)	57.4 (29.8-81.3)	73.8 (64.6-81.8)	82.2 (60.1-94.6)	94.4 (73.9-99.7)	97.9 (94.6-99.4)	99 (98.4-99.4)	99.3 (97.9-99.8)
Democratic Republic of the Congo	38.8 (4.4-85.7)	49.1 (19.6-77.8)	61.9 (52-71.5)	70.6 (48.8-86.3)	87 (46.5-99.1)	95 (85.8-98.7)	98 (96.9-98.7)	98.8 (97.1-99.6)
Cote d'Ivoire	13.1 (7.1-22)	21.8 (16.7-27.5)	34.8 (23.5-45.2)	47.2 (27.2-65.2)	79.7 (66.9-88.9)	87.6 (83.6-91)	92.5 (88.8-95.5)	94.8 (89.8-97.8)
Dominican Republic	89.2 (82.2-94.2)	93.7 (91.8-95.3)	96.3 (94.8-97.5)	97.5 (95.5-98.8)	67.2 (41.1-89)	99.1 (98.4-99.5)	100 (100-100)	100 (100-100)
Egypt	6.6 (4.2-10)	25.3 (21.5-29.6)	62.2 (56.3-68.1)	85.1 (79.1-90.3)	59.4 (48.9-69.8)	85.6 (83.1-87.9)	96 (95.2-96.8)	98.6 (98-99.1)
Ethiopia	0.1 (0-0.2)	0.5 (0.3-0.9)	3.3 (2.2-4.8)	14.3 (7.2-25.8)	5.4 (1.8-12.9)	19.1 (11.8-28.7)	51.3 (42.6-60.3)	77.6 (65.6-87.1)
Gabon	49.6 (22.5-75.1)	63.3 (48.4-75.6)	75.3 (65.6-83.8)	82.2 (67.5-92.4)	96.6 (91.3-99.1)	98.3 (97.2-99.1)	99.1 (98.5-99.5)	99.4 (98.7-99.8)
Ghana	15.9 (8.1-27.1)	23.1 (16.5-30.2)	33.1 (23.3-43.9)	42.4 (24.9-60.8)	74.7 (62.4-84.2)	89.3 (85.8-91.9)	95.8 (94-97.2)	98.1 (96.6-99)
Guatemala	5.1 (2.5-9.2)	12.2 (8.5-17.2)	27.3 (18.3-37.1)	45.3 (27.3-63.9)	89.2 (82.3-93.9)	93 (90.8-95)	95.5 (93.6-97)	96.7 (94.3-98.3)
Guinea	6.7 (2.6-14.9)	10.4 (6.8-15.2)	16.7 (11.6-23)	24.1 (12-40)	54.9 (30.1-78.4)	67.4 (56.4-77.6)	77.6 (67.7-84.8)	83.4 (66.4-93.2)
Haiti	1.8 (1.1-2.6)	3.9 (3.1-4.9)	8.7 (7.2-10.6)	15.8 (11.7-20.7)	55 (41.3-68.1)	66.3 (59-73.2)	76 (70.9-81.2)	82.1 (75.1-88)
Honduras	7 (0.3-36.9)	19.5 (5-44.2)	53.3 (43.1-63.7)	79.5 (54.5-94.2)	88.1 (48.9-99.3)	95.5 (87.4-98.8)	98.1 (97.1-98.8)	98.8 (96.7-99.7)
India	3.7 (1.8-7)	12.3 (8.3-17.5)	34.9 (24.8-45.7)	60.7 (43.8-76.2)	58.4 (43.4-70.9)	79.8 (74.1-84.8)	91.7 (88.3-94.5)	96.1 (93.3-98.1)
Indonesia	1.5 (0.8-2.6)	6.9 (5.1-9.2)	27.1 (22.1-31.5)	57.6 (47.1-67.2)	34.3 (17-42.1)	68.7 (58.1-73.1)	90.5 (87.7-91.7)	96.8 (94.7-97.6)
Jordan	76.8 (65.5-86.2)	90.7 (87.4-93.4)	96.6 (95.1-97.6)	98.5 (97.5-99.1)	94.3 (87.7-97.9)	99.4 (98.9-99.7)	99.9 (99.9-100)	100 (100-100)
Kazakhstan	97.6 (91.9-99.7)	94.8 (89.6-97.5)	81.7 (26.8-99)	67.1 (2.3-99.6)	100 (100-100)	100 (100-100)	100 (99.9-100)	99.8 (99.4-100)
Kenya	17.6 (10.3-27.5)	20.7 (15.7-26.9)	24.6 (17.6-32.7)	28.3 (17-43)	65.5 (49.4-79.4)	78.7 (72.1-84.5)	87.5 (81.8-91.6)	92 (85.2-96)
Kyrgyz Republic	63.8 (41-81.7)	95.3 (92.7-97.2)	99.6 (99.3-99.8)	99.9 (99.9-100)	94.8 (88.9-96.6)	99.7 (99.5-99.8)	100 (100-100)	100 (100-100)
Lesotho	9.4 (1.1-31.1)	21 (8.9-38.1)	45.2 (35.4-54.6)	67.3 (45.9-85.2)	46.9 (14.3-82.9)	74.3 (57.3-88)	91 (87.4-93.6)	96.2 (92.5-98.4)
Liberia	1 (0.1-5.1)	5.9 (1.6-15)	32 (24.3-40.5)	71.6 (57.4-82.4)	55.5 (13.9-91.6)	65.7 (39.4-87.1)	75.3 (67.5-82.1)	80.7 (68-89.4)
Madagascar	30.5 (14.5-52.1)	22.6 (16.6-30.9)	16.9 (10.4-25.4)	13.7 (5.3-30)	31.6 (15.9-53.3)	49.3 (40.8-59.1)	67.6 (56.1-78.1)	78.7 (60.3-91.1)
Malawi	28 (15.3-42.5)	52 (42.3-62.2)	75.5 (66.9-82.8)	87.4 (78.2-93.5)	69.2 (55.7-81)	85.8 (81.2-89.6)	94.2 (92-95.9)	97.2 (95.2-98.4)
Maldives	0.2 (0-1.1)	16.6 (3.9-41.9)	96.8 (94.8-98.2)	99.9 (99.9-100)	100 (100-100)	100 (100-100)	100 (100-100)	96.9 (94.4-98.4)
Mali	8.9 (3.6-17.2)	17.3 (11.8-23.6)	32.1 (22.8-43.7)	47.5 (28.7-70.2)	69.6 (53.2-82.8)	83.6 (78.7-88.2)	91.8 (88.2-94.4)	95.3 (91.2-97.8)
Morocco	3.4 (1.5-4.3)	17.9 (12.1-20.2)	58.1 (34.6-67)	84.3 (58.2-91.9)	67.5 (50.4-81.4)	89.7 (85.4-93.3)	97.1 (93.4-98.9)	98.9 (96.3-99.8)
Mozambique	10.7 (5-19)	21.1 (15-28.2)	38.3 (30.7-45.7)	54.9 (42.1-68.5)	74 (59.6-85.9)	87.6 (83.6-91.3)	94.5 (92.6-96.1)	97.2 (95.4-98.4)
Namibia	45.8 (33.3-59)	57.2 (49.2-64.6)	67.9 (58.2-75.4)	75.1 (61.8-84.9)	91.8 (87.6-94.8)	96 (94.9-96.8)	98 (97.4-98.5)	98.9 (98.2-99.3)
Nepal	0.5 (0.2-0.9)	2.6 (1.7-3.6)	13.6 (9.9-17.9)	39.7 (25.7-54.4)	12.4 (6.2-21.3)	40.9 (32.3-50.1)	77.8 (71.8-82.9)	92.6 (87.9-95.7)

Nicaragua	51.1 (6.6-94.4)	32.6 (23.7-42.8)	23.8 (0.8-83.3)	21.9 (0-97.1)	33.5 (2.4-44.1)	96.4 (94.4-96.8)	99.8 (98.5-100)	99.9 (99.3-100)
Niger	1.9 (0.5-5.1)	4.4 (2.5-7.2)	10.9 (6.8-16.9)	21.9 (9.1-41.6)	56.4 (30-79.8)	62.6 (50-74.4)	68.1 (57.2-77.5)	71.6 (51.1-87.2)
Nigeria	26 (6.9-56.8)	14.2 (7.3-24.2)	7.4 (5.4-10.1)	4.7 (1.9-9.6)	81.9 (54.7-95.7)	82.5 (70.7-90.7)	81.9 (76.4-86.8)	80.5 (65.1-91.1)
Pakistan	1.9 (1.1-2.8)	6.8 (5.1-8.7)	22.2 (18.2-26.6)	45.9 (37.6-54.3)	43.9 (31.2-58.3)	66.1 (58.2-73.7)	83.1 (78.4-87.1)	91 (87-94.1)
Peru	6.9 (4.1-10.5)	21.6 (17.2-26.3)	51.1 (43.8-57.6)	75.1 (64.9-82.9)	80.7 (61.3-92.6)	97.8 (96.5-98.7)	99.8 (99.6-99.9)	100 (99.9-100)
Philippines	3.6 (1.7-6.5)	11.2 (7.7-15.1)	30.2 (22.7-38.6)	53.6 (40-68.5)	56.2 (39.3-72.1)	80.2 (73.8-85.9)	92.7 (89.9-95)	96.9 (94.7-98.4)
Rwanda	7.7 (3-16.3)	25.2 (17.2-35.6)	59 (46.9-69.9)	81.7 (68-91.3)	24.3 (11.7-41.3)	60.4 (49.4-70.2)	88.2 (83.2-92.5)	96.3 (93.1-98.4)
Senegal	15.8 (9.2-24.7)	25.9 (19.9-32.3)	39.8 (36-43.9)	52.6 (46.3-59.2)	79.7 (62.3-91.5)	90.3 (84.8-94.3)	95.6 (94.3-96.6)	97.7 (96.7-98.5)
Sierra Leone	0.4 (0-2.4)	2.6 (0.3-9.6)	29.4 (21.2-38.6)	81.2 (55.5-94.5)	1.6 (0-12.2)	8.8 (1.1-29.5)	54.6 (43.8-64.6)	90.7 (75.3-97.8)
South Africa	53.5 (32-74.7)	73.8 (62.3-82.8)	87.4 (81.5-91.8)	93.3 (87.9-96.7)	99.1 (98-99.7)	99.1 (98.5-99.5)	99.1 (98.6-99.4)	99 (98.2-99.5)
Tajikistan	7.8 (0-12.1)	18.6 (1.2-30.4)	50.9 (29.9-59.6)	81.4 (68.4-85.8)	41.7 (0.7-96)	66.1 (13.4-95.5)	89.1 (77.9-95)	96.1 (92.7-98.2)
Tanzania	21.5 (15.5-28.4)	28.1 (24-32.1)	36 (32.5-39.4)	42.9 (36-49.1)	71.1 (60.9-79.7)	83.9 (80.3-87)	91.6 (90.1-93.2)	95.2 (93.4-96.7)
Timor-Leste	0.3 (0-1.2)	0.9 (0.1-3.3)	5.5 (3.1-8.7)	27.1 (13.8-46.7)	2.3 (0-15.5)	11.9 (1.6-40.4)	60.1 (45.6-73.5)	92.5 (85.4-96.8)
Togo	17.7 (8.1-34)	26.9 (18.7-38)	39.5 (29.3-50.9)	50.9 (33.4-67.7)	81.5 (65.9-92.3)	92.1 (87.7-95.4)	96.8 (94.8-98)	98.4 (96.6-99.2)
Turkey	19.6 (11.1-30.9)	54.5 (45.9-62.9)	85.7 (78.8-90.1)	95.5 (91.2-97.8)	77.4 (57.8-84.9)	98.7 (97.9-99)	99.9 (99.9-100)	100 (100-100)
Uganda	9.2 (4.6-16.3)	22 (15.6-28.7)	44.6 (36-54.1)	65 (50.7-77.6)	53.5 (39-67.2)	74.5 (68.2-80.5)	88.1 (84.6-91.3)	93.9 (90.5-96.3)
Vietnam	21.4 (4.4-56.5)	42.5 (32-53.3)	67.6 (27.6-93)	78.9 (19.8-99.1)	45.5 (14.4-81.6)	97.6 (96.4-98.5)	99.9 (99.7-100)	100 (99.9-100)
Yemen	15.3 (0-0)	14.6 (0-0)	8.9 (0-0.1)	79.8 (0-100)	53.3 (0-100)	53.6 (0-100)	55.8 (0-98.9)	47 (0-95.8)
Zambia	9.9 (4.6-17.4)	20.9 (15.4-27.7)	40 (31.3-50)	58 (41.8-74.3)	87.4 (78.4-94)	91.6 (88.5-94.1)	94.3 (91.9-96.1)	95.7 (92.2-97.8)
Zimbabwe	53.3 (34.3-71.1)	54.3 (44.2-64.5)	55.2 (45.6-65.1)	55.9 (40-70.6)	91.7 (83.5-96.4)	93.8 (90.9-96)	95.2 (92.9-96.9)	95.9 (92.5-98.1)

Supplement to: Hasan MM, Magalhaes RJS, Fatima Y, Ahmed S, Mamun AA. Levels, trends and inequalities in using institutional delivery services in low- and middle-income countries: a stratified analysis by facility type. *Glob Health Sci Pract*. 2021;9(1). https://doi.org/10.9745/GHSP-D-20-00533

Table S5: Change rates of institutional delivery services in low- and middle-income countries by place of residence

	Rural				Urban			
**Country**	**1990-1999**	**2000-2009**	**2010-2018**	**1990-2018**	**1990-1999**	**2000-2009**	**2010-2018**	**1990-2018**
Afghanistan	4.7	7.8	7.8	6.8	3.5	3.7	2.1	3.1
Albania	3.7	1.1	0.2	1.7	0.2	0.1	0.0	0.1
Angola	-2.2	-2.3	-1.7	-2.1	-0.6	-1.3	-2.5	-1.4
Armenia	32.1	2.7	0.0	11.6	2.7	0.1	0.0	0.9
Bangladesh	16.1	14.9	11.1	14.2	6.1	5.0	3.6	5.0
Benin	2.1	1.5	0.9	1.5	1.0	0.6	0.4	0.7
Bolivia	5.8	4.7	3.2	4.7	3.8	1.7	0.7	2.1
Brazil	3.3	0.8	0.2	1.4	1.4	0.1	0.0	0.5
Burkina Faso	4.3	3.6	2.5	3.5	0.2	0.1	0.1	0.1
Burundi	15.6	14.4	5.2	12.0	10.8	6.1	1.3	6.2
Cambodia	29.4	23.9	6.4	20.5	27.6	13.1	1.3	14.5
Cameroon	-0.2	-0.2	-0.2	-0.2	0.3	0.2	0.2	0.2
Chad	6.1	6.3	6.2	6.2	3.3	2.8	2.2	2.8
Colombia	3.2	1.7	0.9	2.0	1.4	0.4	0.1	0.6
Comoros	6.9	4.5	2.3	4.6	2.9	1.6	0.7	1.8
Republic of the Congo	3.6	2.5	1.1	2.4	0.9	0.4	0.1	0.5
Democratic Republic of the Congo	4.2	3.8	2.1	3.4	2.4	1.2	0.5	1.4
Cote d'Ivoire	2.9	2.6	2.1	2.5	0.6	0.5	0.4	0.5
Dominican Republic	0.7	0.3	0.1	0.4	0.1	0.0	0.0	0.0
Egypt	12.3	7.0	2.5	7.4	5.3	2.3	0.8	2.8
Ethiopia	15.9	16.5	15.1	15.9	11.2	8.0	3.5	7.8
Gabon	0.5	0.4	0.2	0.4	0.6	0.3	0.2	0.3
Ghana	4.3	3.7	2.7	3.6	1.3	0.8	0.5	0.9
Guatemala	6.5	5.2	3.6	5.2	2.4	1.5	0.9	1.6
Guinea	3.4	3.5	3.3	3.4	1.5	1.2	0.9	1.2
Haiti	6.7	6.3	5.5	6.2	1.8	1.6	1.3	1.6
Honduras	9.9	8.3	3.1	7.3	4.2	1.8	0.5	2.2
India	9.8	7.3	4.0	7.1	3.3	2.0	1.1	2.1
Indonesia	11.3	9.0	5.4	8.7	5.3	2.9	1.4	3.3
Jordan	2.4	0.5	0.1	1.0	0.8	0.3	0.1	0.4
Kazakhstan	0.0	-0.3	-0.7	-0.3	0.4	0.0	0.0	0.1
Kenya	1.5	1.4	1.3	1.4	0.8	0.6	0.5	0.6
Kyrgyz Republic	1.0	0.3	0.1	0.5	1.4	0.1	0.0	0.5
Lesotho	7.6	6.3	3.3	5.8	0.4	0.3	0.1	0.3
Liberia	16.1	15.5	8.2	13.5	2.0	1.8	1.2	1.7
Madagascar	-1.8	-1.8	-1.8	-1.8	0.9	0.8	0.7	0.8
Malawi	5.4	3.0	1.4	3.3	1.2	0.6	0.4	0.7
Maldives	37.8	11.3	0.2	16.9	0.0	0.0	-0.2	-0.1
Mali	5.5	4.7	3.6	4.7	2.1	1.1	0.6	1.3
Morocco	9.4	6.7	3.4	6.6	3.4	1.5	0.6	1.9
Mozambique	4.5	3.8	2.8	3.7	0.8	0.5	0.4	0.6
Namibia	2.1	1.5	1.0	1.5	0.6	0.3	0.2	0.4
Nepal	14.6	13.1	8.8	12.3	4.6	3.4	2.1	3.4
Nicaragua	1.5	1.6	0.7	1.3	3.9	0.7	0.0	1.5
Niger	7.2	7.1	6.3	6.9	1.0	0.8	0.5	0.8
Nigeria	0.0	0.1	0.1	0.1	0.1	0.1	0.1	0.1
Pakistan	11.0	9.2	6.1	8.9	4.7	3.3	2.0	3.4
Peru	11.2	7.7	3.7	7.7	3.3	1.1	0.4	1.6
Philippines	10.3	8.0	4.7	7.8	4.8	3.1	1.7	3.2
Rwanda	12.3	8.2	3.3	8.1	4.8	2.5	1.0	2.8
Senegal	5.2	3.9	2.6	3.9	1.1	0.7	0.4	0.8
Sierra Leone	18.1	23.9	11.9	18.5	16.2	18.3	6.6	14.2
South Africa	3.1	1.4	0.6	1.7	0.2	0.1	0.1	0.1
Tajikistan	7.6	7.4	3.3	6.2	6.6	4.7	1.4	4.3
Tanzania	1.5	1.4	1.2	1.4	0.3	0.2	0.2	0.2
Timor-Leste	11.8	17.4	16.2	15.2	16.4	18.0	6.7	14.1
Togo	4.1	3.3	2.3	3.2	1.3	0.7	0.3	0.8
Turkey	7.7	3.1	0.9	4.0	5.0	1.1	0.2	2.1
Uganda	6.6	5.1	3.3	5.0	1.4	0.9	0.6	1.0
Vietnam	7.8	2.9	0.7	3.9	14.3	0.2	0.0	4.7
Yemen	5.1	5.0	4.5	4.9	1.8	1.6	1.4	1.6
Zambia	4.7	4.0	3.0	3.9	1.2	0.8	0.5	0.8
Zimbabwe	0.5	0.4	0.3	0.4	0.0	0.0	0.0	0.0

Table S6: Trends in the utilization (%, 95% credible intervals) of institutional delivery services in low- and middle-income countries by place of residence

	Rural				Urban			
**Country**	**1990**	**2000**	**2010**	**2018**	**1990**	**2000**	**2010**	**2018**
Afghanistan	7.5 (0.1-58.9)	12.2 (1.2-48.5)	26.8 (16.9-39.1)	50 (26.4-71.7)	33.2 (0.7-92.5)	47.1 (8.9-86.8)	67.5 (53.9-78.3)	80 (60.4-91.7)
Albania	61.7 (15.3-95)	87.8 (67.5-97.3)	97.5 (95.8-98.7)	99.3 (98.7-99.6)	97.5 (86.5-99.4)	99.1 (97.1-99.6)	99.6 (99.4-99.7)	99.8 (99.6-99.8)
Angola	33.2 (4.7-76.7)	26.7 (9.5-52.2)	21.2 (14.5-29.3)	18.5 (9.4-31.6)	91.6 (65.7-99.3)	86.4 (67.7-96.1)	75.8 (66.7-83.6)	62.2 (43.4-78.1)
Armenia	3.9 (1-10.1)	78.1 (65-87.6)	99.7 (99.6-99.8)	100 (100-100)	77.3 (47.4-94.2)	98.9 (97.9-99.5)	100 (99.9-100)	100 (100-100)
Bangladesh	0.9 (0.7-1.2)	4.5 (3.9-5)	19.5 (17.1-21.9)	47.4 (40.6-54.3)	15 (10.1-21.8)	27.5 (22.9-32.4)	45.1 (38.5-51.1)	60.3 (49.1-69.8)
Benin	56 (40.2-71.7)	68.9 (61.7-76.5)	79.5 (74.4-84.2)	85.6 (78.9-91.1)	76.6 (63.2-86.8)	84.6 (79.3-89)	90.1 (87.1-92.6)	93 (89.3-95.8)
Bolivia	17.5 (10.5-27)	31.2 (26.4-36.7)	49.8 (38.6-61.6)	64.5 (46.1-80.2)	52.7 (35.5-62.9)	76.4 (71.2-79.5)	90.1 (83.6-93.3)	95.2 (88.2-97.8)
Brazil	64.3 (45.3-79.6)	87.4 (69.6-96.1)	93.9 (64-99.8)	95.5 (56.7-100)	87.1 (74-88.2)	99 (96.7-99.2)	99.8 (98.7-100)	99.9 (99.3-100)
Burkina Faso	23.6 (12.1-39.5)	36.3 (28.1-46)	51.9 (35.8-67.2)	63.5 (36.2-83.9)	89.8 (81.2-94.8)	91.4 (88.5-93.7)	92.4 (87.8-95.9)	92.8 (84.9-97.5)
Burundi	3.1 (0.1-11.2)	14.8 (2.7-34.9)	59.6 (45.1-70.1)	90.3 (82.8-94.1)	16.9 (0.6-52.6)	49.3 (13.9-76.9)	87.5 (79.9-91.8)	97.2 (94.7-98.4)
Cambodia	0.3 (0.1-0.8)	5.6 (3.2-9.1)	56.2 (46.6-64.4)	93.6 (88.1-96.9)	1.7 (0.4-4.3)	26.2 (15.5-37.4)	88.9 (84.5-92.3)	98.9 (97.9-99.6)
Cameroon	46.7 (36.1-57.8)	45.7 (40-51.1)	44.7 (35-53.9)	43.9 (29.5-58.8)	81.1 (73.9-87.4)	83.5 (79.9-86.8)	85.4 (80.4-89.5)	86.7 (78.6-92.4)
Chad	3.1 (1.4-6)	5.7 (3.7-8.1)	10.7 (7.3-15.2)	17.5 (9.3-28.4)	28.4 (14.9-45)	39.2 (29.6-48.3)	51.7 (42.1-61)	61.5 (44.6-74.6)
Colombia	53.3 (43.6-62.2)	72.6 (67.9-76.5)	86 (82.4-89.5)	92.3 (89.1-95.1)	83.6 (75.9-89.4)	95.6 (94.3-96.7)	98.9 (98.5-99.2)	99.6 (99.4-99.8)
Comoros	22.9 (11.2-40.5)	45.1 (33.9-56.9)	70 (57.7-80.2)	83.9 (69.1-93.1)	56.7 (36.8-75.3)	75.4 (65.9-83.3)	87.7 (81.4-92.6)	93.1 (86.6-97)
Republic of the Congo	44.3 (8.4-87.4)	63.4 (37.7-83.9)	80.5 (72.2-86.5)	88 (72-96.4)	86.1 (48.7-98.9)	94.1 (85-98.2)	97.3 (96-98.3)	98.2 (94.6-99.5)
Democratic Republic of the Congo	30.7 (2.6-80.5)	47 (17.8-76.2)	67.9 (58.2-76.5)	80.3 (61.3-92.7)	64.8 (13.4-96.1)	81.5 (54.7-94.9)	91.9 (88.3-94.8)	95.4 (89.5-98.4)
Cote d'Ivoire	26.5 (17.3-37.9)	35.4 (29-41.6)	45.7 (35.9-54.7)	54.2 (37-69.7)	74.6 (63.8-83.7)	79.3 (74.5-83)	83.2 (78.1-87.6)	85.6 (77.5-91.8)
Dominican Republic	88.8 (83-93.2)	95.1 (93.6-96.2)	97.8 (96.7-98.7)	98.9 (97.8-99.5)	98.5 (97.4-99.2)	99.1 (98.8-99.4)	99.4 (99.1-99.7)	99.6 (99.2-99.8)
Egypt	11.5 (9.6-13.5)	38.4 (35.9-41)	75 (71.8-78)	91.3 (89-93.2)	43.4 (37.1-49.2)	72.6 (70-75.2)	90.1 (88.4-91.6)	96.1 (94.8-97.1)
Ethiopia	0.3 (0.1-0.8)	1.4 (0.7-2.5)	7.2 (4.6-10.4)	24.1 (12.5-40.3)	9.6 (2.8-23.4)	29.3 (17.9-43.2)	63.8 (55-72.2)	84.6 (73.3-91.9)
Gabon	64.8 (37.8-87)	68 (55.5-79.5)	70.5 (59.5-80.6)	71.8 (49.1-86.1)	88.2 (71.1-96.3)	93.3 (88.9-96.1)	96 (93.8-97.6)	97.2 (93.9-99)
Ghana	22.1 (12.7-33.8)	34 (26.9-41.3)	48.8 (38.3-58.3)	60.7 (43.3-75.3)	71.1 (57.9-81.8)	80.4 (74.9-85)	87 (82.2-90.9)	90.7 (84.2-95.1)
Guatemala	14.9 (9.2-23.4)	28.4 (22.6-34.5)	47.6 (38.3-57.3)	63.6 (48.4-76.2)	55.6 (43.3-67)	70.1 (63.2-76.1)	81.3 (74.9-86.4)	87.6 (79.9-92.9)
Guinea	13.6 (5.6-28.8)	19.1 (13.1-27.2)	27.2 (19.7-36)	35.4 (19.4-55)	53.1 (31-73.6)	61.7 (51-71.5)	69.6 (60.4-76.5)	74.8 (59.8-85.6)
Haiti	5.8 (3.8-8.4)	11.3 (9.1-14)	21.1 (17.6-24.9)	32.7 (25.2-40.2)	39.4 (26.5-53)	46.9 (39.5-54.7)	54.8 (47.8-61.4)	60.8 (49-70.6)
Honduras	11.5 (0.5-52.1)	30.9 (10.4-61.4)	69.2 (58.3-78)	88.3 (68.4-97.2)	52.7 (6.4-94)	79.1 (51.5-93.9)	93.8 (90.7-95.9)	97.3 (91.8-99.4)
India	10.3 (5.3-17.5)	27.4 (20.7-35.1)	55.8 (45.5-66.2)	76.5 (62.8-86.7)	49 (34.1-63.2)	67.4 (60.1-74.9)	81.7 (75.6-87.2)	89 (81.7-94)
Indonesia	6.1 (4.3-8.3)	18.8 (15.9-22)	45.2 (38.5-51.3)	69.4 (60.1-77.3)	36.4 (26.3-48.3)	61 (54.4-66.9)	81.1 (76.1-85.7)	90.4 (85.8-94.4)
Jordan	75.8 (67-82.8)	95 (93.7-96.1)	99.1 (98.9-99.3)	99.8 (99.7-99.9)	88.8 (82-93.5)	95.9 (94.3-97)	98.5 (97.9-98.9)	99.3 (98.9-99.6)
Kazakhstan	97.2 (90.9-99.5)	97.1 (94.3-98.7)	93.6 (61.1-99.7)	88.5 (19.6-99.9)	96.4 (89.2-99.2)	100 (100-100)	100 (100-100)	100 (100-100)
Kenya	32.6 (20.7-45.8)	37.7 (30.9-44.5)	43.3 (34.2-52.9)	48 (32.8-64.1)	68.6 (56.5-79.9)	74 (67.3-80.1)	78.6 (71.7-84.8)	81.5 (71.2-89.5)
Kyrgyz Republic	87.8 (73.2-94.8)	96.8 (94.8-98.1)	99.2 (98.7-99.5)	99.7 (99.4-99.9)	87.3 (76.4-94.9)	99.5 (99.2-99.7)	100 (100-100)	100 (100-100)
Lesotho	15.5 (2-45.2)	33.2 (15.7-53.9)	61.5 (52.3-69.5)	79.9 (63.1-91.5)	82.8 (58-95.8)	86.1 (75.3-93.2)	88.3 (84.8-91.1)	89.2 (80.8-94.7)
Liberia	1.7 (0.1-7)	8.7 (2.8-20.9)	39.8 (31.6-49)	76.9 (64.4-86.7)	47.7 (9.9-87.5)	58.5 (31.2-82.7)	69.8 (60.9-77.7)	76.9 (64.2-86.6)
Madagascar	40.7 (28-52.9)	34.1 (27.4-40.7)	28.4 (18.7-39.9)	24.6 (12.1-42.8)	45.1 (29.4-61.5)	49.3 (40.6-58.8)	53.5 (38.2-67.3)	56.6 (32.9-78)
Malawi	35.7 (21.2-52.5)	60.6 (49.9-69.3)	81.2 (73.9-87.2)	90.6 (83.4-95.3)	77.5 (62.8-88.1)	86.7 (81.6-90.9)	92.4 (88.6-95.2)	95 (90.6-97.7)
Maldives	0.9 (0-4.3)	35.2 (11.4-66.2)	98.2 (97.1-98.9)	100 (99.9-100)	100 (100-100)	100 (100-100)	100 (100-100)	98.5 (97.3-99.2)
Mali	15.4 (8.4-24.5)	26.8 (20.7-33.2)	42.8 (35.4-50.2)	57 (42.9-69.7)	65.1 (43.1-80.7)	79.6 (72.6-85.4)	88.9 (83.6-92.9)	93.2 (86.3-97.1)
Morocco	12.1 (6.3-20.5)	30.8 (22.8-40)	59.2 (38.5-78.9)	77.7 (49.9-94.4)	56.3 (38.1-72.5)	78.3 (69.9-84.9)	90.4 (79.4-96.5)	94.9 (83-99.1)
Mozambique	22.3 (11.5-36.2)	35 (26.6-44)	50.9 (41.4-59.3)	63.4 (48.5-76.8)	76.5 (62.1-88.1)	82.7 (76.5-87.9)	87.2 (83-90.5)	89.9 (83.5-94.4)
Namibia	54.9 (44.4-64.9)	67.4 (61.8-72.1)	77.7 (71.8-82.7)	84 (75.8-89.9)	86.9 (80.2-92)	92.2 (89.8-94)	95.3 (93.6-96.7)	96.9 (94.6-98.3)

Nepal	1.8 (0.9-3.4)	7.9 (5.5-10.5)	28.7 (22.7-35.4)	58 (45.8-69.9)	30.1 (16.5-48.2)	47.6 (37.3-57.2)	66.2 (58.1-73.9)	78.1 (66.7-87.1)
Nicaragua	40.6 (4.2-91)	47.3 (37-57.6)	54.9 (5.3-96.3)	58 (0.8-99.7)	61.9 (11.9-95.6)	89.3 (84.7-92.7)	95.1 (71.9-99.8)	95.4 (53.4-100)
Niger	4.3 (2.2-7.3)	8.8 (6.4-11.8)	17.9 (11.5-25.9)	29.5 (15.8-47.7)	67 (52-79.9)	74.2 (66.7-80.4)	79.9 (71-86.7)	83.5 (70.1-92.1)
Nigeria	24.7 (17.2-33.7)	24.8 (20.3-29.5)	25 (20-30.7)	25.3 (18.1-35.1)	60.4 (48.1-71.9)	61 (53.6-67.8)	61.4 (53.2-68.7)	61.7 (49.4-72.3)
Pakistan	5.1 (3.2-7.7)	15.2 (11.9-19.3)	37.8 (32.7-44.2)	61.7 (53.9-70)	30.9 (21-43.5)	49.3 (40.6-57.5)	68 (61.3-73.6)	79.8 (72.8-85.1)
Peru	9.6 (6.7-13.2)	29.1 (25.4-32.8)	61.6 (56.8-66)	82.6 (76.8-87.1)	62.6 (55.7-68.9)	85.6 (83.7-87.3)	95.4 (94.7-96.1)	98.3 (97.8-98.7)
Philippines	8.2 (4.3-13.8)	22.7 (16.9-28.8)	49.8 (41.4-58)	72.3 (60.5-82.1)	34.2 (22-49)	54.9 (46.3-63.1)	74.2 (67.3-79.9)	84.9 (77.2-90.6)
Rwanda	9.1 (3.6-17.9)	30.5 (21.1-41.4)	66.9 (56.3-77.7)	86.9 (76.3-94.3)	42.2 (21.3-64.2)	67.7 (55.8-77.7)	86 (79.1-90.9)	93.3 (86.3-97.1)
Senegal	24 (21.3-27)	40.2 (38-42.3)	58.9 (57.4-60.5)	72.4 (70.4-74.3)	76.3 (70.1-81.6)	85.4 (82.9-87.6)	91.3 (90.3-92.3)	94.4 (93.3-95.4)
Sierra Leone	0.5 (0-2.8)	3 (0.4-11.7)	31.7 (23.2-41.1)	82.4 (58.3-95.1)	1.7 (0-10)	8.9 (1.2-27.5)	52.5 (42.9-62.5)	89.3 (73.8-97.4)
South Africa	59.9 (37.8-78.6)	80.7 (71.8-87.6)	92.1 (88.6-94.7)	96.3 (93.1-98.1)	92.9 (85-97.2)	94.9 (91.8-96.9)	96.1 (94.6-97.4)	96.9 (94.7-98.4)
Tajikistan	15.5 (0.1-74.7)	33.3 (3.7-77.9)	68.4 (49.5-82.4)	88.7 (80.1-94.8)	28.3 (0.6-94.1)	54.5 (11.9-93.8)	85.6 (73.4-94.1)	95.6 (91.5-97.9)
Tanzania	35.3 (25.9-45.7)	41 (35.1-46.8)	47 (40.2-53.6)	51.9 (41-62.8)	81 (74.4-86.6)	83.4 (80-86.2)	85.4 (82.4-88.2)	86.8 (82.3-90.8)
Timor-Leste	0.7 (0-4.9)	2.2 (0.2-10.6)	12.7 (7-22)	46.6 (28.1-65)	1.7 (0-12)	9.2 (0.9-34.5)	52.8 (37.3-68)	90.4 (82.2-95.8)
Togo	27.9 (11.9-47.6)	41.7 (29.1-54.8)	57.6 (46-68.1)	69.2 (49.2-83.6)	76.6 (57.1-90)	87.3 (80.4-92.5)	93.3 (89.8-95.8)	96 (92.3-98.2)
Turkey	31.5 (19.6-46.9)	66.3 (58.8-73.6)	89.4 (84.5-93.1)	96.3 (92.9-98.3)	55.1 (38.9-72.8)	88.1 (83.8-91.6)	97.8 (96.6-98.6)	99.4 (98.9-99.7)
Uganda	17.2 (9.2-27.4)	33 (25.8-41.2)	54.5 (46.3-62.8)	70.7 (58.3-81.3)	69.5 (54.9-80.6)	79.6 (73.9-84.2)	86.8 (83.1-89.9)	90.8 (85.7-94.4)
Vietnam	31.9 (6.5-67.2)	67.9 (57.4-76.7)	89.2 (65.2-98.6)	94.5 (64.9-99.9)	26.7 (6-62.8)	98.1 (97.3-98.8)	100 (99.9-100)	100 (100-100)
Yemen	7.9 (4.2-14.1)	13.1 (8.8-18.8)	21.4 (13.6-30.7)	30.6 (16.6-47.9)	34.8 (21.1-50.7)	41.6 (31.6-52.4)	49 (35.6-61.8)	54.9 (36.3-72.4)
Zambia	19.4 (11.8-29.1)	30.8 (24.8-37.8)	45.7 (34.6-56.8)	58.1 (39.4-74.7)	71.8 (61.1-81.3)	80.7 (75.9-84.6)	87.1 (82.2-91.1)	90.7 (84.4-94.9)
Zimbabwe	59.7 (42.7-74.4)	62.6 (52.3-70.7)	65.3 (55.4-72.8)	67.1 (51.3-78.7)	91.3 (84.3-96.1)	91.5 (87.7-94.2)	91.4 (87.6-94.1)	91.1 (84.2-95.4)

Supplement to: Hasan MM, Magalhaes RJS, Fatima Y, Ahmed S, Mamun AA. Levels, trends and inequalities in using institutional delivery services in low- and middle-income countries: a stratified analysis by facility type. *Glob Health Sci Pract*. 2021;9(1). https://doi.org/10.9745/GHSP-D-20-00533

Table S7: Change rates of institutional delivery services in low- and middle-income countries by education

Country	Below secondary education				Secondary+ education			
	1990-1999	2000-2009	2010-2018	1990-2018	1990-1999	2000-2009	2010-2018	1990-2018
Afghanistan	-1.0	-2.3	-4.1	-2.4	1.2	1.1	0.5	0.9
Albania	2.4	0.8	0.2	1.2	0.9	0.1	0.0	0.4
Angola	-1.9	-3.3	-4.3	-3.1	-0.1	-0.6	-3.3	-1.2
Armenia	14.4	0.4	0.0	4.8	21.1	1.1	0.0	7.3
Bangladesh	14.8	14.3	11.9	13.7	6.6	5.6	4.2	5.5
Benin	1.7	1.2	0.8	1.2	0.1	0.0	0.0	0.1
Bolivia	6.4	4.6	2.7	4.6	2.0	1.0	0.4	1.2
Brazil	2.3	0.5	0.1	1.0	0.1	-0.1	-0.2	-0.1
Burkina Faso	3.7	3.0	2.1	3.0	0.6	0.3	0.2	0.4
Burundi	14.3	13.7	5.1	11.4	6.4	3.2	0.8	3.6
Cambodia	29.3	24.0	6.8	20.6	20.2	11.3	2.2	11.5
Cameroon	-0.4	-0.4	-0.4	-0.4	0.5	0.3	0.2	0.3
Chad	3.6	3.7	3.6	3.6	0.3	0.3	0.2	0.3
Colombia	2.1	1.3	0.7	1.4	0.6	0.2	0.1	0.3
Comoros	5.9	4.0	2.2	4.1	2.3	1.2	0.6	1.4
Republic of the Congo	2.7	1.8	0.9	1.8	7.6	2.4	0.4	3.6
Democratic Republic of the Congo	2.5	2.3	1.4	2.1	1.7	1.1	0.4	1.1
Cote d'Ivoire	2.1	1.8	1.4	1.8	0.0	-0.1	-0.1	-0.1
Dominican Republic	0.4	0.2	0.1	0.3	0.0	0.0	0.0	0.0
Egypt	9.2	6.0	2.9	6.1	4.4	2.2	1.0	2.6
Ethiopia	14.8	15.1	13.3	14.5	8.9	5.7	2.5	5.8
Gabon	0.8	0.6	0.4	0.6	0.6	0.3	0.2	0.4
Ghana	2.9	2.6	2.0	2.5	0.9	0.6	0.4	0.7
Guatemala	4.4	3.6	2.7	3.6	0.4	0.3	0.2	0.3
Guinea	2.4	2.5	2.3	2.4	0.5	0.4	0.3	0.4
Haiti	2.8	2.8	2.7	2.8	0.0	-0.1	-0.1	-0.1
Honduras	6.5	5.4	2.2	4.8	1.3	0.5	0.2	0.7
India	8.4	6.9	4.4	6.7	2.9	1.8	1.0	1.9
Indonesia	9.8	8.0	5.2	7.8	4.2	2.8	1.6	2.9
Jordan	1.3	0.6	0.3	0.7	0.8	0.2	0.1	0.4
Kazakhstan	0.0	0.0	0.0	0.0	0.0	-0.2	-0.6	-0.3
Kenya	1.9	1.8	1.6	1.8	1.1	0.8	0.6	0.9
Kyrgyz Republic	0.0	0.0	0.0	0.0	0.9	0.2	0.1	0.4
Lesotho	5.6	5.1	3.2	4.7	5.5	3.3	1.3	3.4
Liberia	11.6	11.0	6.3	9.8	4.4	3.4	1.8	3.2
Madagascar	-2.2	-2.3	-2.3	-2.3	0.8	0.7	0.6	0.7
Malawi	4.7	2.7	1.3	3.0	0.2	0.1	0.1	0.1
Maldives	70.0	37.5	0.2	37.2	0.0	0.0	0.0	0.0
Mali	3.4	2.9	2.2	2.8	0.1	0.1	0.1	0.1
Morocco	7.7	4.8	2.2	5.0	0.9	0.4	0.2	0.5
Mozambique	2.9	2.4	1.8	2.4	0.0	0.0	-0.1	0.0
Namibia	1.2	1.0	0.8	1.0	0.5	0.3	0.2	0.4
Nepal	14.1	13.1	9.5	12.4	6.6	4.8	2.8	4.8
Nicaragua	0.2	-0.3	-0.2	-0.1	3.5	0.4	-0.1	1.3
Niger	3.3	3.3	3.0	3.2	0.1	0.1	0.0	0.1
Nigeria	-1.0	-0.9	-0.9	-0.9	-0.4	-0.5	-0.6	-0.5
Pakistan	8.7	7.4	5.3	7.2	2.8	1.9	1.1	2.0
Peru	8.5	5.9	3.1	5.9	2.3	1.0	0.4	1.3
Philippines	7.7	6.9	5.4	6.7	5.7	3.7	2.0	3.9
Rwanda	12.0	8.0	3.2	7.9	5.6	2.2	0.7	2.9
Senegal	3.4	2.4	1.6	2.5	0.2	0.2	0.1	0.2
Sierra Leone	15.7	21.1	10.8	16.3	13.4	14.7	5.4	11.5
South Africa	2.8	1.4	0.6	1.7	0.6	0.3	0.2	0.4
Tajikistan	7.0	8.0	4.2	6.6	6.7	6.4	2.7	5.4
Tanzania	1.0	0.9	0.8	0.9	0.4	0.4	0.3	0.4
Timor-Leste	10.6	15.8	15.4	14.0	8.7	10.8	7.1	9.0
Togo	3.4	2.6	1.8	2.6	1.1	0.6	0.3	0.7
Turkey	6.2	2.2	0.6	3.0	2.8	0.4	0.1	1.1
Uganda	5.8	4.6	3.1	4.5	2.1	1.4	0.8	1.4
Vietnam	9.8	4.6	1.2	5.3	7.0	1.6	0.3	3.0
Yemen	4.2	4.1	3.7	4.0	-1.4	-2.0	-2.6	-2.0
Zambia	2.2	1.9	1.6	1.9	0.4	0.3	0.2	0.3
Zimbabwe	0.0	0.0	0.0	0.0	-0.2	-0.3	-0.4	-0.3

Table S8: Trends in utilization (%, 95% credible intervals) of institutional delivery services in low- and middle-income countries by education

	Below secondary education				Secondary+ education			
**Country**	**1990**	**2000**	**2010**	**2018**	**1990**	**2000**	**2010**	**2018**
Afghanistan	79.1 (12.5-99.6)	71.5 (24.8-95.9)	56.2 (41.6-69.5)	40.4 (19.5-65.3)	61.4 (5-98.5)	69.1 (24.8-95.2)	77 (65.5-86.5)	80.1 (61.6-91.7)
Albania	71.7 (23.7-96.7)	90.4 (73.7-97.5)	97.5 (95.8-98.5)	99.1 (98.4-99.5)	90.5 (62.8-99.3)	98.7 (96.4-99.7)	99.8 (99.7-99.9)	100 (99.9-100)
Angola	71.5 (29.8-95.6)	58.8 (32.7-81.6)	42.2 (32.9-52.5)	29.9 (17.7-44.4)	99.7 (98.7-100)	98.6 (96.4-99.6)	92.1 (88.3-94.7)	71 (53.1-84.3)
Armenia	25.8 (2.7-70.7)	96.3 (90.6-98.9)	100 (99.9-100)	100 (100-100)	12.9 (4.8-27.2)	90.2 (84.4-94.1)	99.8 (99.8-99.9)	100 (100-100)
Bangladesh	0.7 (0.4-1.1)	3.1 (2.3-3.9)	12.7 (9.7-16.2)	32.9 (23.7-44.7)	11.5 (8.7-14.7)	22.1 (19.3-24.6)	38.3 (33.4-42.7)	53.8 (45.4-61)
Benin	61.1 (44.9-74.7)	72.4 (64.2-78.9)	81.3 (76.4-85.8)	86.5 (79.7-91.8)	97.2 (94.1-99)	97.9 (96.8-98.8)	98.4 (97.6-98.9)	98.6 (97.3-99.3)
Bolivia	20.5 (12.5-30.4)	38.5 (33.4-44.2)	60.5 (48.9-71)	75.3 (58.4-87.2)	69 (53.5-81.9)	83.9 (79.8-87.9)	92.2 (87.2-95.9)	95.5 (89.6-98.6)
Brazil	74 (59.3-85)	91.8 (80.9-97.5)	96.3 (78.9-99.9)	97.3 (77.1-100)	97.8 (95.8-98.9)	98.3 (95.3-99.6)	97.6 (86.4-100)	96.2 (71.7-100)
Burkina Faso	29.2 (16-46.5)	42.1 (33.2-51.7)	56.6 (41.8-70.8)	67 (44.1-85.4)	88.9 (83.5-93)	94.1 (92.6-95.3)	96.9 (95.4-97.9)	98.1 (96.5-99)
Burundi	3.8 (0.1-22.4)	15.9 (2.8-44.9)	60.1 (45.5-73.8)	90.1 (82.6-94.9)	36 (3.4-88.9)	67.4 (32.1-92.3)	91.1 (85.1-95)	97.2 (95.1-98.6)
Cambodia	0.3 (0.1-0.7)	5.4 (3.2-8.2)	54.1 (45.3-62.4)	92.9 (87.8-96.4)	3.8 (0.9-9.8)	28 (17-40.8)	81 (74.4-86.5)	96.6 (92.9-98.6)
Cameroon	51.1 (39.6-61.7)	49.4 (42.8-55.5)	47.6 (37.6-57.1)	46.2 (30.4-61.6)	84.5 (74.9-91.6)	88.4 (84.9-91.6)	91 (86.3-94.6)	92.5 (85.5-96.9)
Chad	7.7 (3.7-13.4)	11 (7.6-15.3)	16 (11.4-21.2)	21.4 (12.5-31.4)	53.5 (38.4-68.8)	55 (47.1-62.9)	56.5 (48.7-63.9)	57.6 (45.2-70.1)
Colombia	62 (50.9-71.4)	75.8 (70.6-80)	85.7 (81.4-89.4)	90.9 (86.2-94.4)	90.9 (87.8-93.2)	95.9 (95.1-96.6)	98.2 (97.7-98.6)	99.1 (98.7-99.4)
Comoros	26.1 (12.2-43.3)	46.5 (35.7-58.1)	68.7 (57.8-78)	81.7 (67-91)	63.7 (42.1-80.9)	79.7 (71.1-86.3)	89.6 (84.2-93.6)	94 (87.3-97.6)
Republic of the Congo	52.4 (11-92.2)	68.4 (44.1-87.9)	81.8 (74-87.8)	87.6 (70.8-96)	36.6 (4.7-84.1)	76.2 (50.6-91.7)	95.7 (93.7-97.1)	98.9 (97-99.7)
Democratic Republic of the Congo	42.9 (5.4-89.5)	55.3 (26.6-82.7)	69.3 (59.6-77.9)	77.5 (60.9-91)	68.1 (18.1-96.9)	80.8 (57.3-94.7)	89.4 (85.6-92.9)	92.6 (82.9-97.5)
Cote d'Ivoire	39 (26.5-53.2)	48.2 (41-55.5)	57.7 (47.7-67.3)	64.7 (47.7-78.2)	82.2 (73-89.3)	81.9 (77.2-85.7)	81.1 (73.6-86.8)	80.2 (66.9-89.2)
Dominican Republic	91.9 (87.8-95.1)	95.8 (94.6-96.9)	97.8 (96.6-98.6)	98.6 (97.4-99.3)	99.2 (98.7-99.6)	99.6 (99.5-99.7)	99.8 (99.7-99.9)	99.9 (99.8-99.9)
Egypt	15 (12.4-18.2)	37.1 (34.6-39.8)	66.3 (62.9-69.3)	83.8 (80-86.7)	45.1 (38.2-52.6)	69.4 (66.2-72.4)	86.2 (83.5-88.5)	93.3 (90.8-95.1)
Ethiopia	0.5 (0.1-1.3)	2.2 (1.2-3.8)	10 (7-13.8)	29.1 (16.9-44)	16.9 (8.1-31.7)	40.8 (31.3-52)	70.8 (64.8-76.5)	86.8 (79.8-91.6)
Gabon	73.3 (49-90.9)	79.6 (69.4-88.1)	84.2 (76.9-89.6)	86.7 (73.7-94.3)	86.9 (70.1-95.8)	92.5 (87.9-95.6)	95.5 (93-97.3)	96.8 (92.3-98.8)
Ghana	28.6 (17.7-42)	38.3 (30.5-46)	49.4 (39.5-59.3)	58.2 (42.2-72.9)	68.9 (50.6-83.3)	75 (66.9-82.2)	79.9 (70.2-87.3)	82.8 (69.1-92.3)
Guatemala	22.3 (13.7-32.5)	34.5 (27.8-41.4)	49.4 (38.4-59.1)	61.4 (45.8-74.6)	84.2 (73.2-92.2)	87.7 (82.3-91.6)	90.2 (85.6-93.6)	91.6 (84.5-95.9)
Guinea	21.5 (9.3-38.9)	27.2 (19.1-36.1)	34.8 (26.3-44.1)	41.7 (23.5-61.1)	68.4 (48.9-83.9)	71.8 (64.2-79.5)	74.6 (66.5-81.4)	76.3 (60.3-87.6)
Haiti	11.8 (7.3-18)	15.6 (12-19.7)	20.6 (16.9-25.1)	25.6 (18.2-34.6)	61 (50.2-70.8)	60.7 (55.1-66.3)	60.4 (55.5-65)	60.1 (52.5-68.2)
Honduras	22.4 (1.2-74)	43.2 (15.8-72.6)	72.9 (64-80.8)	86.9 (67.5-97)	80.9 (36.3-98.8)	91.5 (80.2-97.8)	96.1 (94.2-97.5)	97.4 (91.1-99.4)
India	10.2 (5.1-18.1)	23.5 (17-30.7)	46.2 (35.4-58.4)	65.8 (49.1-80.7)	51.6 (34.9-67.6)	68.7 (60-76.6)	81.8 (74.7-87.8)	88.6 (80.5-94.4)
Indonesia	7.2 (4.9-10.1)	19.1 (15.5-23.1)	41.9 (34.3-49.7)	63.7 (51.4-73.9)	37.1 (27.7-48.2)	56.2 (49.9-62.9)	73.8 (67.1-79.6)	83.9 (75.3-89.7)
Jordan	79.4 (68.8-88.2)	89.8 (85.9-92.8)	95.2 (93.2-96.8)	97.4 (95.5-98.6)	89.5 (83.5-93.8)	96.7 (95.5-97.5)	99 (98.6-99.3)	99.6 (99.3-99.8)
Kazakhstan	100 (100-100)	100 (100-100)	100 (99.9-100)	99.8 (99.3-100)	98.2 (94-99.7)	98.3 (96.5-99.3)	95.9 (72.9-99.9)	91.6 (28.9-100)
Kenya	29.4 (18.3-41.4)	35.5 (28.4-42.8)	42.4 (33.2-51.8)	48.1 (33.3-63.7)	65 (50.9-78.2)	72.6 (65.7-78.8)	78.8 (71.6-85.1)	82.7 (72.2-90.5)
Kyrgyz Republic	100 (100-100)	100 (100-100)	100 (100-100)	100 (100-100)	89.2 (78.8-95.6)	97.4 (96.1-98.4)	99.4 (99.1-99.7)	99.8 (99.6-99.9)
Lesotho	19 (2.9-50.5)	33.4 (15.9-52.8)	54.8 (45.7-64.3)	70.9 (51.5-85.6)	34.8 (10.1-68)	59.9 (41.3-75.7)	82 (76.7-86)	91.3 (83-95.9)
Liberia	4.7 (0.4-19.8)	15.2 (5.1-34)	45 (36.2-53.9)	74.5 (60.7-85.5)	33.7 (6-77)	51.9 (26.4-77)	72 (63.6-78.6)	83.5 (74.5-90.6)
Madagascar	39.9 (27-52.9)	32 (25.8-38.1)	25.3 (16.2-35.7)	21 (9.7-36.9)	48.9 (37.5-61.6)	52.9 (46.6-58.8)	56.7 (45.5-67.4)	59.6 (42.1-75.8)
Malawi	39.2 (23.7-58.1)	62 (52-71.4)	80.6 (74.1-86.1)	89.6 (82.7-94.4)	90.9 (81.7-96.1)	92.3 (88.1-95.1)	93.1 (89.6-95.8)	93.5 (87.5-97.2)
Maldives	0 (0-0)	3.3 (0.7-10.3)	98 (96.7-98.8)	100 (100-100)	99.7 (98.5-100)	99.7 (99.1-99.9)	99.7 (99.5-99.8)	99.5 (99.2-99.8)
Mali	27.4 (17.9-39.7)	38.2 (32.4-45)	50.7 (42.1-58)	60.5 (45.9-72.1)	88 (81.7-92)	89 (86.7-91.1)	89.7 (87-92.2)	90.2 (85.7-93.8)
Morocco	21 (11.1-33.6)	44.6 (33.9-55.3)	70.7 (50.1-85.8)	84.3 (60.8-96.3)	83 (72.6-91.2)	90.5 (86.8-93.6)	94.5 (88.7-97.8)	96.2 (88.2-99.2)
Mozambique	33.5 (20.2-49.4)	44.7 (36.2-53.7)	56.8 (49.6-64.2)	65.8 (54.4-76.9)	94.5 (88.6-98)	94.6 (91.7-96.6)	94.4 (92-96.3)	94 (89-97.1)
Namibia	55.8 (45.9-64.6)	62.9 (57.5-67.5)	69.4 (63.8-75.3)	74.1 (65.7-81.7)	85.8 (78.2-91.7)	90.2 (87-92.9)	93.2 (90.5-95.2)	94.8 (91.2-97.2)

Nepal	1.6 (0.7-2.9)	6.4 (4.5-9)	23.6 (18.3-29.7)	50.5 (37.2-65)	20.3 (11-33.1)	39.1 (30.6-48.1)	62.4 (54.8-69.6)	77.8 (67.8-85.9)
Nicaragua	55.1 (9.1-95.3)	56.1 (45.2-66.8)	54.5 (5.8-96.1)	53.8 (0.6-99.4)	66.4 (16.9-97.5)	92.2 (89-94.9)	95.8 (72.1-99.9)	95.3 (46.3-100)
Niger	12.9 (7.4-20.1)	17.9 (13.6-22.9)	24.8 (17.3-33.7)	31.6 (18.3-47.6)	75.6 (63.8-85.1)	76.5 (69.8-82)	77 (68.1-84.5)	77.1 (63.2-88.1)
Nigeria	24.7 (17.9-32.5)	22.5 (18.7-26.6)	20.4 (16.4-25.1)	19 (13.3-25.9)	79.3 (71.9-85.7)	75.9 (71.1-80.5)	71.8 (65.9-77.5)	68.3 (57.1-77)
Pakistan	7.6 (4.6-12)	18.1 (13.8-23.2)	37.5 (32.2-43.6)	57.2 (48.3-65.2)	49.6 (36-63.3)	65.2 (56.7-72.5)	78.2 (72.6-82.6)	85.7 (80.3-90.2)
Peru	15.3 (11-20.2)	35.4 (31.2-39.8)	62.6 (57.9-66.9)	80.3 (74.5-85.4)	68 (61.9-74.1)	84.8 (82.9-86.7)	93.6 (92.6-94.5)	96.9 (96-97.6)
Philippines	7.4 (3.9-12.4)	15.9 (11.7-21)	31.5 (24.7-39.1)	48.4 (35.9-61.5)	28.4 (17-42.5)	50 (41.3-59.1)	71.9 (64.8-78.5)	84.2 (76-90.7)
Rwanda	9.5 (3.6-18.9)	31.1 (21.4-41.7)	67.2 (55.2-77.1)	86.9 (74.9-93.9)	43.1 (23.8-63.2)	74 (64-81.7)	91.5 (87.3-94.6)	96.8 (93.3-98.6)
Senegal	38.6 (33-44)	53.7 (50.3-57.1)	68.2 (66.3-70.2)	77.8 (75.6-80.1)	89.9 (84.8-94)	92.1 (89.7-94.1)	93.8 (92.6-94.9)	94.9 (93.3-96.2)
Sierra Leone	0.9 (0-5.1)	4.3 (0.5-15.5)	34.2 (25.1-44)	81.1 (56.5-94.8)	3.5 (0-23.4)	13.8 (1.9-41.8)	57.9 (48-68.1)	89 (73-96.9)
South Africa	59.5 (37.7-80.1)	78.5 (68.9-86.6)	90.1 (85.9-93.7)	94.8 (90.6-97.4)	87.3 (74-94.5)	92.4 (88.5-95.5)	95.4 (93.3-97.1)	96.8 (94.6-98.5)
Tajikistan	13.6 (0.1-85.2)	27.7 (2.1-81.1)	60.7 (39.5-80.1)	85 (73.5-92.5)	19.9 (0.2-92.4)	39.2 (4.5-89.3)	72.9 (52.8-87.8)	90.3 (81-95.2)
Tanzania	43.3 (33.4-52.8)	47.7 (41.9-53.3)	52.1 (45.7-58.9)	55.6 (45.7-65.8)	79.8 (73.3-85.3)	83.4 (80.2-86)	86.3 (83-88.9)	88.3 (83.4-91.6)
Timor-Leste	0.8 (0-4.7)	2.3 (0.3-9)	11.4 (6.6-18.2)	38.9 (22.5-57.3)	5.7 (0.1-38.9)	13.8 (1.9-45.2)	40.1 (27.3-55.4)	71.1 (51.6-85.3)
Togo	34.9 (17.6-56)	48.8 (35.8-60.5)	63.2 (52-73.6)	73 (57.5-85.7)	79.8 (61.5-91.7)	88.5 (82.8-93.4)	93.6 (90.3-96)	95.9 (91.8-98.2)
Turkey	41.6 (27.6-56.6)	75.3 (68.8-81)	92.9 (89.8-95.1)	97.6 (95.6-98.8)	73.6 (56.7-86.1)	95.8 (94-97.1)	99.5 (99.2-99.7)	99.9 (99.8-100)
Uganda	19.1 (10.2-29.9)	33.8 (26-41.7)	53 (44.8-60.8)	67.9 (54.9-78.5)	59.2 (44.7-73.7)	72.9 (66.2-79.1)	83.2 (78.4-86.8)	88.8 (83.1-93.1)
Vietnam	20.5 (3.9-52.7)	53.7 (43.5-63.4)	83.2 (50.2-97.4)	91.7 (50.8-99.8)	42.2 (11.2-77.1)	82.3 (75.5-87.6)	95.8 (84.3-99.5)	98.1 (87.5-100)
Yemen	10.9 (6-18)	16.7 (11.3-23.3)	25.2 (16.3-35.8)	33.7 (17.9-51.8)	77.1 (65.2-87)	67 (57.9-76.6)	54.8 (42.6-67.2)	44.6 (27.5-63)
Zambia	33.3 (20.9-45.6)	41.3 (33.5-49.8)	50.1 (38.2-61.2)	57 (39.6-73.4)	76.4 (65-85.1)	79.2 (73.6-84.1)	81.4 (74.2-87.2)	82.8 (71.2-90.8)
Zimbabwe	57.7 (39.3-74)	57.7 (48-67)	57.6 (47.7-66.7)	57.4 (41.1-71.6)	87 (77.4-93.6)	85 (79-89.5)	82.2 (75.7-87.3)	79.5 (67.1-87.6)

Supplement to: Hasan MM, Magalhaes RJS, Fatima Y, Ahmed S, Mamun AA. Levels, trends and inequalities in using institutional delivery services in low- and middle-income countries: a stratified analysis by facility type. *Glob Health Sci Pract*. 2021;9(1). https://doi.org/10.9745/GHSP-D-20-00533

Table S9: Change rates of institutional delivery services in low- and middle-income countries by age

	Adolescent, 15-19 years of age				Adult, 20-49 years of age			
**Country**	**1990-1999**	**2000-2009**	**2010-2018**	**1990-2018**	**1990-1999**	**2000-2009**	**2010-2018**	**1990-2018**
Afghanistan	1.8	2.8	3.0	2.5	4.6	7.2	6.5	6.1
Albania	67.2	39.2	0.4	36.8	2.8	0.7	0.1	1.2
Angola	-1.0	-1.5	-2.0	-1.5	-0.5	-0.7	-0.8	-0.7
Armenia	7.3	0.2	0.0	2.4	20.9	1.1	0.0	7.3
Bangladesh	14.7	13.4	9.6	12.7	13.3	11.9	8.6	11.4
Benin	1.8	1.2	0.7	1.2	1.8	1.2	0.7	1.3
Bolivia	3.2	2.1	1.2	2.2	4.0	2.7	1.6	2.8
Brazil	1.3	0.2	0.0	0.5	3.0	0.3	0.0	1.1
Burkina Faso	4.4	3.1	1.9	3.2	3.6	2.9	2.0	2.9
Burundi	10.4	7.4	2.2	6.8	15.6	14.1	4.9	11.9
Cambodia	22.3	16.3	4.6	14.8	27.4	21.2	5.3	18.5
Cameroon	0.2	0.2	0.1	0.2	0.3	0.3	0.2	0.3
Chad	3.4	3.5	3.4	3.4	4.2	4.2	4.0	4.2
Colombia	1.7	0.7	0.3	0.9	1.8	0.8	0.3	1.0
Comoros	5.4	3.4	1.8	3.6	5.8	3.6	1.8	3.8
Republic of the Congo	2.7	1.3	0.5	1.5	4.7	2.3	0.7	2.6
Democratic Republic of the Congo	3.2	2.5	1.3	2.4	2.8	2.3	1.2	2.1
Cote d'Ivoire	1.1	1.0	0.9	1.0	2.2	1.8	1.4	1.8
Dominican Republic	0.4	0.1	0.0	0.2	0.4	0.1	0.1	0.2
Egypt	9.4	4.8	1.7	5.4	8.5	4.5	1.7	5.0
Ethiopia	11.0	11.5	10.0	10.9	12.6	12.7	11.0	12.2
Gabon	0.5	0.3	0.2	0.3	1.2	0.7	0.4	0.7
Ghana	3.5	2.6	1.8	2.7	4.0	2.9	1.9	3.0
Guatemala	4.4	3.2	2.1	3.3	4.4	3.3	2.3	3.4
Guinea	0.6	0.7	0.7	0.7	3.1	3.0	2.6	2.9
Haiti	1.7	1.7	1.6	1.7	4.1	3.7	3.2	3.7
Honduras	7.1	4.3	1.3	4.4	8.1	5.7	1.8	5.3
India	9.5	6.4	3.1	6.5	7.7	5.4	2.9	5.4
Indonesia	10.1	7.8	4.6	7.6	8.9	6.1	3.2	6.2
Jordan	0.3	0.2	0.1	0.2	1.1	0.3	0.1	0.5
Kazakhstan	0.0	0.0	0.0	0.0	0.0	-0.2	-0.5	-0.2
Kenya	2.2	1.9	1.5	1.9	2.0	1.8	1.5	1.8
Kyrgyz Republic	0.0	0.0	0.0	0.0	1.0	0.2	0.1	0.4
Lesotho	8.0	5.7	2.3	5.5	7.3	5.5	2.7	5.3
Liberia	14.3	12.3	5.5	10.9	11.3	10.1	5.3	9.1
Madagascar	-1.2	-1.1	-1.0	-1.1	-1.5	-1.5	-1.5	-1.5
Malawi	5.4	2.6	1.0	3.1	4.8	2.7	1.2	2.9
Maldives	0.0	0.0	0.0	0.0	7.1	1.6	0.2	3.0
Mali	3.2	2.5	1.8	2.5	3.5	2.9	2.2	2.9
Morocco	5.8	3.2	1.4	3.5	7.2	4.1	1.7	4.4
Mozambique	3.5	2.6	1.7	2.7	3.2	2.6	1.9	2.6
Namibia	0.6	0.4	0.3	0.5	2.0	1.2	0.7	1.3
Nepal	16.0	13.0	6.7	12.2	14.2	12.3	7.6	11.6
Nicaragua	1.0	-0.3	-0.5	0.1	2.0	0.6	0.1	0.9
Niger	3.3	3.2	2.9	3.1	3.5	3.4	3.1	3.3
Nigeria	-0.3	-0.2	-0.2	-0.2	0.8	0.8	0.7	0.8
Pakistan	7.4	5.9	3.9	5.8	7.5	6.0	4.0	5.9
Peru	6.6	3.4	1.4	3.8	5.2	2.8	1.2	3.1
Philippines	9.1	6.3	3.2	6.3	6.8	4.9	2.8	4.9
Rwanda	12.9	5.9	1.4	6.9	11.7	7.5	2.9	7.5
Senegal	3.6	2.5	1.6	2.6	3.3	2.3	1.5	2.4
Sierra Leone	17.0	21.8	9.6	16.6	14.7	20.2	9.9	15.4
South Africa	1.4	0.5	0.2	0.7	1.6	0.8	0.4	0.9
Tajikistan	8.1	6.3	1.8	5.5	7.5	6.8	2.9	5.9
Tanzania	1.3	1.1	0.9	1.1	1.3	1.2	1.0	1.2
Timor-Leste	13.0	17.1	13.2	14.6	11.4	15.4	12.1	13.1
Togo	3.1	2.3	1.5	2.3	3.8	2.8	1.7	2.8
Turkey	15.5	1.3	0.0	5.6	6.5	1.9	0.4	3.0
Uganda	4.8	3.4	2.0	3.5	6.2	4.6	2.9	4.6
Vietnam	12.5	4.1	0.7	5.9	7.4	2.4	0.6	3.5
Yemen	4.5	4.1	3.4	4.0	4.5	4.2	3.7	4.2
Zambia	3.1	2.2	1.5	2.3	1.9	1.6	1.3	1.6
Zimbabwe	0.3	0.2	0.1	0.2	0.5	0.4	0.3	0.4

Table S10: Trends in the utilization (%, 95% credible intervals) of institutional delivery services in low- and middle-income countries by age

	Adolescent, 15-19 years of age				Adult, 20-49 years of age			
**Country**	**1990**	**2000**	**2010**	**2018**	**1990**	**2000**	**2010**	**2018**
Afghanistan	27 (0.4-88.2)	32.4 (4.1-76.1)	43 (28.4-57.8)	54.5 (29.9-79.4)	10.3 (0.1-67.6)	16.6 (1.9-55.4)	34.2 (21.9-47.6)	57.7 (32.4-79.7)
Albania	0 (0-0)	2.8 (0.6-7.4)	96.8 (94.9-98.2)	100 (100-100)	70.8 (23.9-95.4)	92.1 (78.9-97.9)	98.4 (97.5-99.1)	99.5 (99.2-99.8)
Angola	70.3 (25.4-95)	63.6 (36.5-84.3)	54.4 (43.1-65.8)	46.3 (27.9-63.6)	56.1 (15.5-91.8)	53.1 (28-78.6)	49.4 (38.9-59.8)	46.3 (29.4-62.9)
Armenia	50.7 (12.2-88.4)	98.2 (95.6-99.5)	100 (99.9-100)	100 (100-100)	13.1 (4.6-25.9)	90.3 (85-94)	99.8 (99.8-99.9)	100 (100-100)
Bangladesh	1.5 (1.1-1.9)	6.4 (5.6-7.3)	23.8 (21.2-26.8)	51.5 (44.7-58.3)	2.1 (1.6-2.8)	7.9 (6.8-8.9)	25.8 (22.2-29.6)	51.4 (43.8-59.8)
Benin	62.6 (47.9-77.4)	74.7 (67.2-81.3)	83.8 (79.2-87.5)	88.8 (83.3-93)	62.3 (46.9-77.5)	74.3 (66.9-80.9)	83.5 (78.8-87.5)	88.6 (82.5-92.8)
Bolivia	44.9 (29.1-60.4)	61.8 (55-68.9)	76 (64.5-85.6)	83.9 (68.4-93.7)	37.3 (24.3-51)	55.3 (49-61.2)	72 (60.9-81.2)	81.7 (65.7-91.7)
Brazil	85.5 (75.4-92.6)	96.8 (91.2-99)	98.8 (92.2-100)	99.2 (92.2-100)	73.6 (57.7-85.3)	97.1 (92.7-99.2)	99.5 (96.9-100)	99.7 (98.5-100)
Burkina Faso	32 (18.3-48.2)	49.2 (39.8-57.9)	66.7 (52.3-79)	77.5 (57.3-91.4)	30.3 (15.8-47.6)	43.4 (34-53.2)	58 (42.9-73)	68 (45-87.6)
Burundi	14 (0.7-66.2)	39.3 (11.4-77.2)	79.4 (68.1-87.6)	94.3 (89-97)	3.3 (0.1-18.3)	15.8 (3-43.6)	61.4 (47.1-73.7)	90.9 (84.4-95.1)
Cambodia	1.5 (0.4-3.6)	13.3 (8.1-20.2)	63.6 (54.4-71.7)	92.2 (85.3-96.5)	0.5 (0.1-1.3)	8.1 (4.7-12.3)	61.4 (51.3-70.1)	94 (88.4-97.2)
Cameroon	59.4 (47.2-71)	60.6 (54-67.4)	61.7 (52-70.4)	62.4 (46.5-75.3)	58.2 (45.4-69.1)	60.1 (53.1-66.6)	61.8 (51.1-71.9)	63 (46.2-76.6)
Chad	11 (4.9-19.9)	15.5 (10.5-21.7)	21.9 (15.8-29.1)	28.7 (16.8-43.3)	8 (3.7-14.7)	12.2 (8.4-16.6)	18.6 (13.5-24.7)	25.6 (15.5-38.2)
Colombia	75.6 (66.4-84.1)	89 (86.4-91.6)	95.4 (93.9-96.7)	97.8 (96.4-98.8)	73.8 (65.3-80.6)	88.2 (85.7-90.3)	95.1 (93.7-96.4)	97.7 (96.4-98.5)
Comoros	31.4 (15.5-50.2)	53.3 (42.1-64.3)	74.2 (62.6-82.9)	85.4 (70.5-93.4)	29.7 (14.7-50.3)	52.5 (41.1-63.7)	74.6 (63-83)	86.2 (74.3-93.7)
Republic of the Congo	62.2 (16.6-93.8)	81.2 (62.4-93)	92.1 (88.6-94.8)	95.7 (88.5-98.8)	45.6 (8.2-89.8)	71.9 (48.1-88.9)	89.8 (84.8-93.4)	95.2 (86.6-98.9)
Democratic Republic of the Congo	43.9 (6.7-85.7)	60.6 (31.8-83.6)	77.4 (69-83.8)	85.7 (72.1-94.2)	46.6 (6-91.7)	61.4 (31-86.7)	76.5 (68.5-83.2)	84.1 (66.2-93.6)
Cote d'Ivoire	44.4 (33.6-56.3)	49.5 (43.8-55)	54.5 (46.1-61.3)	58.5 (44.3-69.8)	41.3 (27.5-55)	51.4 (44.1-58.4)	61.4 (51.5-71.1)	68.6 (54-82.2)
Dominican Republic	94.7 (91.1-97.1)	98.1 (97.4-98.6)	99.3 (98.9-99.6)	99.7 (99.3-99.9)	94.2 (90.8-96.4)	97.5 (96.8-98.1)	98.9 (98.5-99.3)	99.5 (99.1-99.7)
Egypt	20.6 (16.5-25.1)	51.4 (47.6-54.3)	81.1 (78.1-84.1)	92.9 (90.6-94.9)	22.6 (19-26.6)	51.9 (48.7-55)	80 (77.1-82.3)	91.9 (89.6-93.7)
Ethiopia	1.6 (0.3-5.5)	4.9 (2.3-9.6)	15.6 (10.7-22)	34.8 (18.6-53.9)	1.1 (0.3-2.6)	3.8 (2.1-6.3)	13.5 (9.7-18.2)	32.4 (18.7-48.4)
Gabon	85.3 (67.1-95)	89.7 (83.9-93.8)	92.4 (88.7-95.3)	93.8 (87-97.7)	76.2 (52.8-91.7)	85.6 (77.4-91.6)	91.4 (86.7-94.5)	94 (87.2-97.8)
Ghana	35.6 (21.4-52.1)	50.4 (41.7-58.8)	65.3 (54.7-74.7)	75.2 (59.4-86.8)	32.7 (21-45.8)	48.4 (40.9-56.2)	64.5 (55.3-73.1)	75.3 (61.4-85.5)
Guatemala	29.9 (18.9-42.5)	46.1 (37.5-54.3)	63.4 (53.9-72.3)	75 (62.7-85.2)	28.1 (19.4-39.3)	43.2 (36.6-50.9)	59.9 (51.4-68)	71.8 (60.1-82.1)
Guinea	35 (19.5-55.3)	37.2 (29-47)	39.8 (31.6-48.7)	42.1 (26.9-59.5)	21 (8.6-38.9)	28.5 (19.7-38.1)	38.5 (30.2-48.3)	47.3 (29.4-67)
Haiti	25.8 (13.5-41.6)	30.5 (22.6-39.9)	36.1 (28.2-45.1)	41 (28.2-56.7)	14.9 (9.7-21.8)	22.5 (18.1-27.4)	32.6 (27.9-37.5)	42.2 (33.3-50.3)
Honduras	27.6 (2.8-82.3)	55.6 (27.9-83.8)	84.1 (77.2-88.8)	93.5 (82.4-98.2)	20.6 (1-71)	46.3 (18.4-74.7)	79.8 (72.3-86)	92.2 (78.8-98.2)
India	13.6 (6.9-23.6)	34.6 (26.1-44.3)	64.7 (52.5-74.8)	82.8 (69.6-91.4)	17.5 (9.1-28.1)	37.5 (28.6-46.4)	63.3 (53.3-72.7)	79.7 (66.9-89.3)
Indonesia	8.6 (5.7-12.3)	23.2 (19.2-28)	49.7 (41.8-56.7)	71.6 (60.8-80.1)	14.3 (10.1-19)	34.4 (29.4-39.6)	62.3 (55.3-68.3)	80.5 (72.8-86)
Jordan	93.4 (87.4-97.1)	96.2 (94.1-97.7)	97.8 (96.4-98.7)	98.5 (97-99.3)	86.1 (79.2-91.7)	95.5 (93.8-96.7)	98.6 (98.1-99)	99.5 (99.1-99.7)
Kazakhstan	100 (100-100)	100 (100-100)	100 (99.8-100)	99.8 (98.9-100)	98.2 (93.7-99.7)	98.2 (96.5-99.3)	96 (79.4-99.9)	92.2 (41-100)
Kenya	38.4 (26.3-52.5)	47.9 (40.4-56.1)	57.7 (49.1-65.9)	65 (50.8-76.9)	35.9 (23.1-50.5)	43.8 (35.4-51.8)	52.1 (42.8-61.6)	58.6 (43.7-72.1)
Kyrgyz Republic	100 (100-100)	100 (100-100)	100 (100-100)	100 (100-100)	88.4 (76.4-96)	97.3 (95.7-98.6)	99.4 (99-99.6)	99.8 (99.6-99.9)
Lesotho	19.1 (2.8-54.9)	42.3 (22-64.6)	73.3 (65.9-80.3)	88.2 (74.3-95.5)	18.9 (3.6-48.9)	38.8 (20.6-58.7)	66.5 (57.8-74.4)	82.7 (68.4-92.3)
Liberia	3.9 (0.3-15.2)	16.3 (4.8-34.6)	54 (44.7-62.5)	83.8 (73.9-90.7)	6.1 (0.7-23.5)	18.9 (6.8-39.1)	50.9 (41.5-60.6)	78 (65.7-87.3)
Madagascar	36.3 (24.2-49.9)	32.3 (26.1-39.1)	28.9 (18.3-41.4)	26.7 (12.3-46.2)	42.9 (29.3-57.1)	37 (29.8-43.6)	31.7 (20.7-43.3)	28.2 (13.2-47)
Malawi	40.2 (25.4-55.2)	68.1 (59.7-75.6)	87.3 (82.3-91.2)	94.5 (90.4-97.2)	39.8 (24.3-56.2)	63.4 (53.9-72.2)	82 (74.7-87.7)	90.6 (82.4-95.2)
Maldives	100 (99.9-100)	100 (100-100)	100 (100-100)	100 (100-100)	43.2 (5.2-87)	85.1 (60.8-96)	98.4 (97.4-99)	99.7 (99.5-99.9)
Mali	35.3 (22.1-50.9)	48.4 (40.7-56)	62 (53.9-69.6)	71.5 (59-82.4)	27.9 (17.5-40.1)	39.4 (33.1-46)	52.4 (45.1-59.6)	62.5 (49.1-73.7)
Morocco	33.2 (19.5-50)	58.5 (47.4-68.5)	79.5 (60.3-91.2)	89 (68.6-97.6)	25.6 (14.3-38.3)	51.9 (41.1-63.6)	76.7 (57.8-90.4)	88 (67.3-97.4)
Mozambique	36.6 (21.4-51.5)	51.8 (42.8-60.1)	67 (61.1-73)	77 (67.8-84.5)	33.8 (20-51.5)	46.4 (37.5-56.2)	59.9 (52.1-66.8)	69.6 (56.9-79.2)
Namibia	76.5 (64.5-86)	81.1 (75.3-85.8)	84.7 (78.7-89.3)	86.9 (78.2-93.1)	62.6 (51.9-72.1)	75.9 (71.1-80.1)	85.5 (81.5-89.1)	90.6 (86-94.3)

Nepal	2.5 (1.3-4.3)	12.4 (9.3-16.1)	44.3 (37.9-51.2)	75.8 (66.6-84)	2.5 (1.3-4.6)	10.5 (7.6-13.8)	35.2 (27.8-43.2)	64.7 (51.6-76.4)
Nicaragua	64.6 (13.8-96.9)	71.2 (61.9-78.9)	69 (13-97.9)	66.4 (2-99.8)	55.7 (9.3-94)	67.6 (57.4-76.7)	71.8 (15.5-98.3)	72.1 (3-99.8)
Niger	15.4 (8.5-24.6)	21.4 (16.1-27.5)	29.5 (20.4-39.9)	37.2 (21.6-56.2)	13.7 (7.9-21.1)	19.3 (14.8-24.9)	27.1 (19.6-36.5)	34.6 (20.7-50.7)
Nigeria	25.7 (18-33.7)	25 (20.9-29.2)	24.4 (19.9-29.9)	24.1 (17.3-32.8)	32.7 (26.2-39.2)	35.3 (31-39.4)	38.1 (33.2-43.2)	40.4 (33.1-49.2)
Pakistan	13 (7.5-20.9)	27.1 (20.4-34.2)	48.4 (40.5-55.8)	66.2 (56.1-75)	12.6 (7.2-19.7)	26.7 (19.8-34)	48.1 (40.6-55.6)	66.2 (55.3-75.2)
Peru	31.5 (23.5-39.5)	59.8 (54.9-64.3)	82.9 (79.7-85.8)	92.5 (89.9-94.8)	38.4 (30.4-45.9)	63.9 (59.7-67.8)	83.4 (80.7-85.8)	92 (89.4-94.2)
Philippines	14.1 (7.2-24.1)	34.5 (25.5-43.5)	63.4 (53.6-71.5)	81.6 (70.8-88.7)	19.8 (11.5-29.8)	38.6 (30.4-46.6)	62.1 (54-69.4)	77.7 (67.6-85.1)
Rwanda	13.9 (6-26.5)	48.9 (37.6-60.2)	85.6 (78.7-90.8)	96.1 (92.3-98.4)	10.7 (4.8-20)	34 (24.5-44.6)	69.8 (59.6-79)	88.2 (78.9-94.3)
Senegal	39.4 (31-48.5)	56.3 (51-61.4)	72 (69-74.9)	81.7 (78.1-84.7)	40.8 (35.8-45.9)	56.4 (53.4-59.4)	71 (69.1-72.8)	80.2 (78-82.2)
Sierra Leone	0.8 (0-5.6)	4.7 (0.5-17.6)	39.8 (30.3-49.9)	85.7 (65.2-96.7)	1.1 (0-7.6)	5.1 (0.6-19)	37.2 (28-46.8)	82.5 (60-94.8)
South Africa	81.4 (66.3-92.3)	92.9 (89.2-96.1)	97.5 (96.4-98.4)	98.9 (98.1-99.5)	74.6 (57.1-88.1)	87 (80.6-92.3)	93.8 (91-96)	96.6 (93.9-98.2)
Tajikistan	20.1 (0.3-89.4)	45.1 (7.1-90.8)	82.2 (67.5-92.4)	95.2 (90.7-97.6)	17.3 (0.1-75.5)	36.7 (3.8-79)	71.3 (52.2-84.3)	90 (82.9-94.7)
Tanzania	49.5 (38.5-60)	56.2 (49.9-61.7)	62.6 (56.6-68)	67.4 (58.1-75.6)	42.8 (32.1-53.7)	48.8 (43-54.9)	54.8 (48-61.4)	59.5 (49.4-69.4)
Timor-Leste	1 (0-6.5)	3.8 (0.5-14.5)	20.9 (13-31.4)	60.2 (41-76.4)	1.5 (0-12)	4.8 (0.6-19.5)	22.6 (14-34.8)	59.6 (38.9-76.6)
Togo	41.5 (22-64.1)	56.5 (43.7-68.1)	70.7 (60.2-80)	79.4 (64.2-89.7)	36 (17.5-59.8)	52.6 (40-66.3)	69 (58.3-78.3)	79.2 (64.3-89)
Turkey	21 (6.4-43.4)	88.5 (81.4-93.6)	99.6 (99.2-99.8)	100 (99.9-100)	43.1 (28.2-58.9)	80.3 (74.6-85.1)	95.7 (93.6-97.3)	98.8 (97.8-99.5)
Uganda	30.1 (18.7-42.7)	48.4 (40.3-55.5)	67.3 (61.3-73.2)	79.3 (71.1-86.3)	20.3 (11.5-31.8)	37.4 (29.9-45.6)	58.8 (50.9-66.2)	73.9 (62.3-82.7)
Vietnam	18.6 (3.6-50.5)	62.2 (50.9-72.5)	91.3 (67.2-99)	96.5 (74.4-100)	35.5 (8.7-71.9)	72.6 (63.3-80)	91.2 (69.4-98.9)	95.3 (68.8-99.9)
Yemen	14.7 (7.8-23.7)	23 (16.3-31)	34.5 (23.9-45.4)	45.2 (29.3-61.4)	11.7 (6.1-19.7)	18.4 (12.8-25.7)	28 (18.1-38.7)	37.7 (21.3-54.9)
Zambia	41.1 (27.9-55.3)	55.7 (47.4-63.5)	69.4 (59-78.6)	78.1 (63.5-88.5)	40.6 (27.8-54.5)	49.2 (41-56.8)	57.9 (47.2-68.4)	64.3 (48.2-78.8)
Zimbabwe	70.3 (52.1-83.8)	72.4 (63.1-79.9)	74 (65.3-81.5)	74.9 (60.6-86.1)	67.6 (51.3-80.7)	70.7 (63.2-77.9)	73.4 (65.5-80.1)	75.2 (62.6-85.3)

Supplement to: Hasan MM, Magalhaes RJS, Fatima Y, Ahmed S, Mamun AA. Levels, trends and inequalities in using institutional delivery services in low- and middle-income countries: a stratified analysis by facility type. *Glob Health Sci Pract*. 2021;9(1). https://doi.org/10.9745/GHSP-D-20-00533

Figure S1: Changes of absolute inequalities in the utilization of institutional delivery services between earliest and latest DHS rounds by place of residence

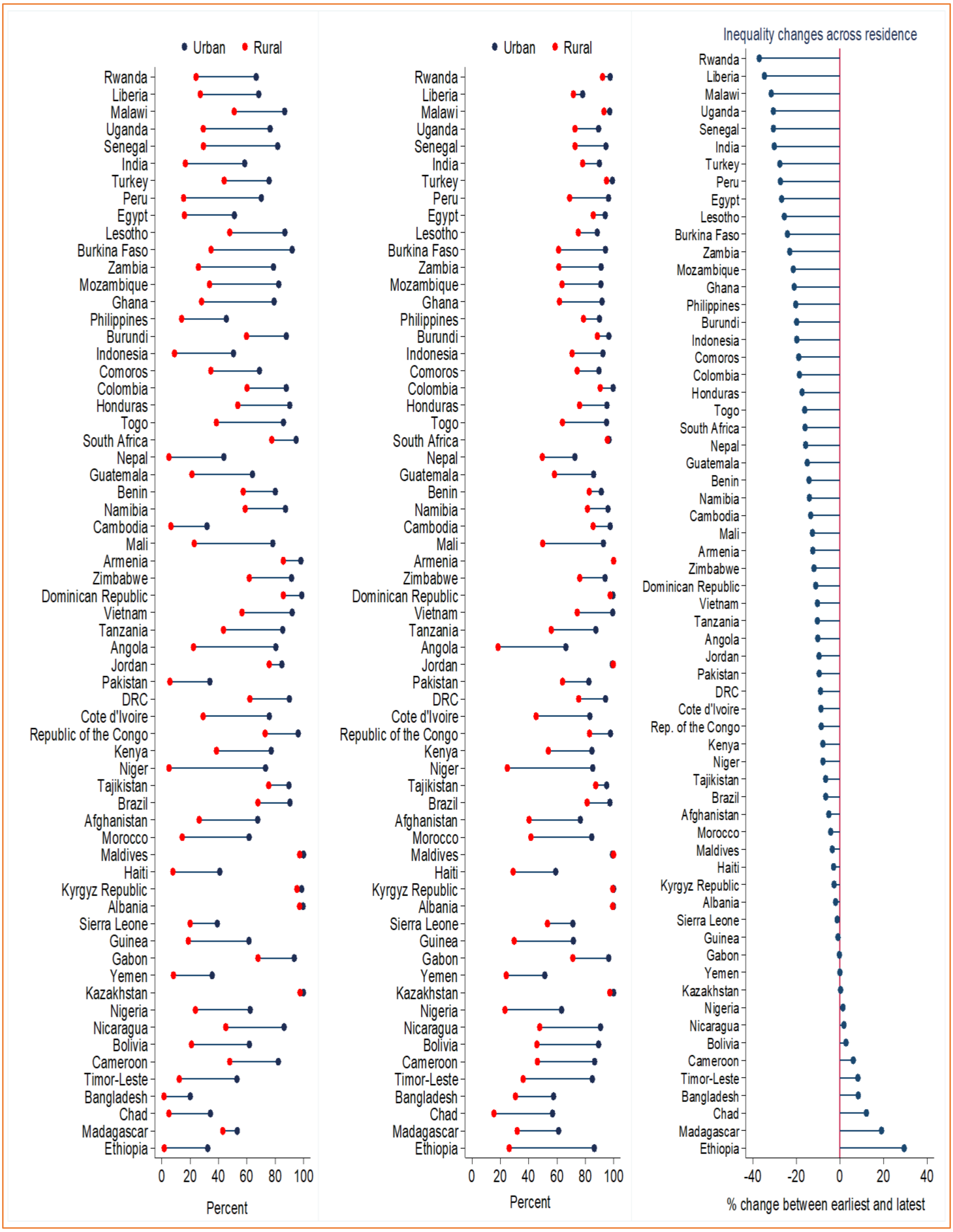
Urban
Rural
Rwanda
Liberia
Malawi
Uganda
Senegal
India
Turkey
Peru
Egypt
Lesotho
Burkina Faso
Zambia
Mozambique
Ghana
Philippines
Burundi
Indonesia
Comoros
Colombia
Honduras
Togo
South Africa
Nepal
Guatemala
Benin
Namibia
Cambodia
Mali
Armenia
Zimbabwe
Dominican Republic
Vietnam
Tanzania
Angola
Jordan
Pakistan
DRC
Cote d'Ivoire
Republic of the Congo
Kenya
Niger
Tajikistan
Brazil
Afghanistan
Morocco
Maldives
Haiti
Kyrgyz Republic
Albania
Sierra Leone
Guinea
Gabon
Yemen
Kazakhstan
Nigeria
Nicaragua
Bolivia
Cameroon
Timor-Leste
Bangladesh
Chad
Madagascar
Ethiopia
0 20 40 60 80 100
Percent
Inequality changes across residence
Rep. of the Congo
-40 -20 0 20 40
% change between earliest and latest

Supplement to: Hasan MM, Magalhaes RJS, Fatima Y, Ahmed S, Mamun AA. Levels, trends and inequalities in using institutional delivery services in low- and middle-income countries: a stratified analysis by facility type. *Glob Health Sci Pract*. 2021;9(1). https://doi.org/10.9745/GHSP-D-20-00533

Figure S2: Changes of absolute inequalities in the utilization of institutional delivery services between earliest and latest DHS rounds by education

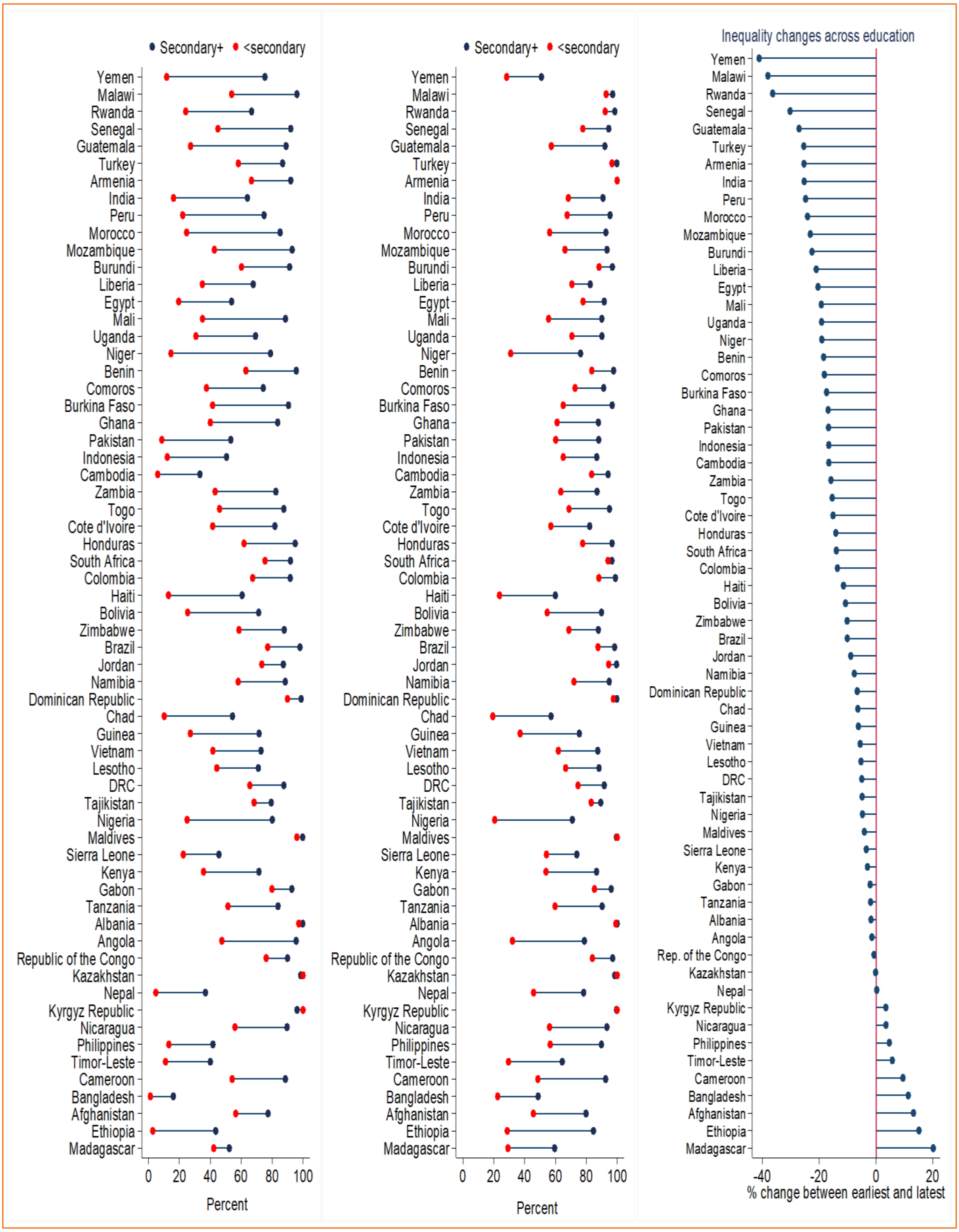
Secondary+ <secondary
Percent
Secondary+ <secondary
Percent
Inequality changes across education
% change between earliest and latest
Yemen
Malawi
Rwanda
Senegal
Guatemala
Turkey
Armenia
India
Peru
Morocco
Mozambique
Burundi
Liberia
Egypt
Mali
Uganda
Niger
Benin
Comoros
Burkina Faso
Ghana
Pakistan
Indonesia
Cambodia
Zambia
Togo
Cote d'Ivoire
Honduras
South Africa
Colombia
Haiti
Bolivia
Zimbabwe
Brazil
Jordan
Namibia
Dominican Republic
Chad
Guinea
Vietnam
Lesotho
DRC
Tajikistan
Nigeria
Maldives
Sierra Leone
Kenya
Gabon
Tanzania
Albania
Angola
Republic of the Congo
Rep. of the Congo
Kazakhstan
Nepal
Kyrgyz Republic
Nicaragua
Philippines
Timor-Leste
Cameroon
Bangladesh
Afghanistan
Ethiopia
Madagascar
0 20 40 60 80 100
-40 -20 0 20

Supplement to: Hasan MM, Magalhaes RJS, Fatima Y, Ahmed S, Mamun AA. Levels, trends and inequalities in using institutional delivery services in low- and middle-income countries: a stratified analysis by facility type. *Glob Health Sci Pract*. 2021;9(1). https://doi.org/10.9745/GHSP-D-20-00533

Figure S3: Changes of absolute inequalities in the utilization of institutional delivery services between earliest and latest DHS rounds by age

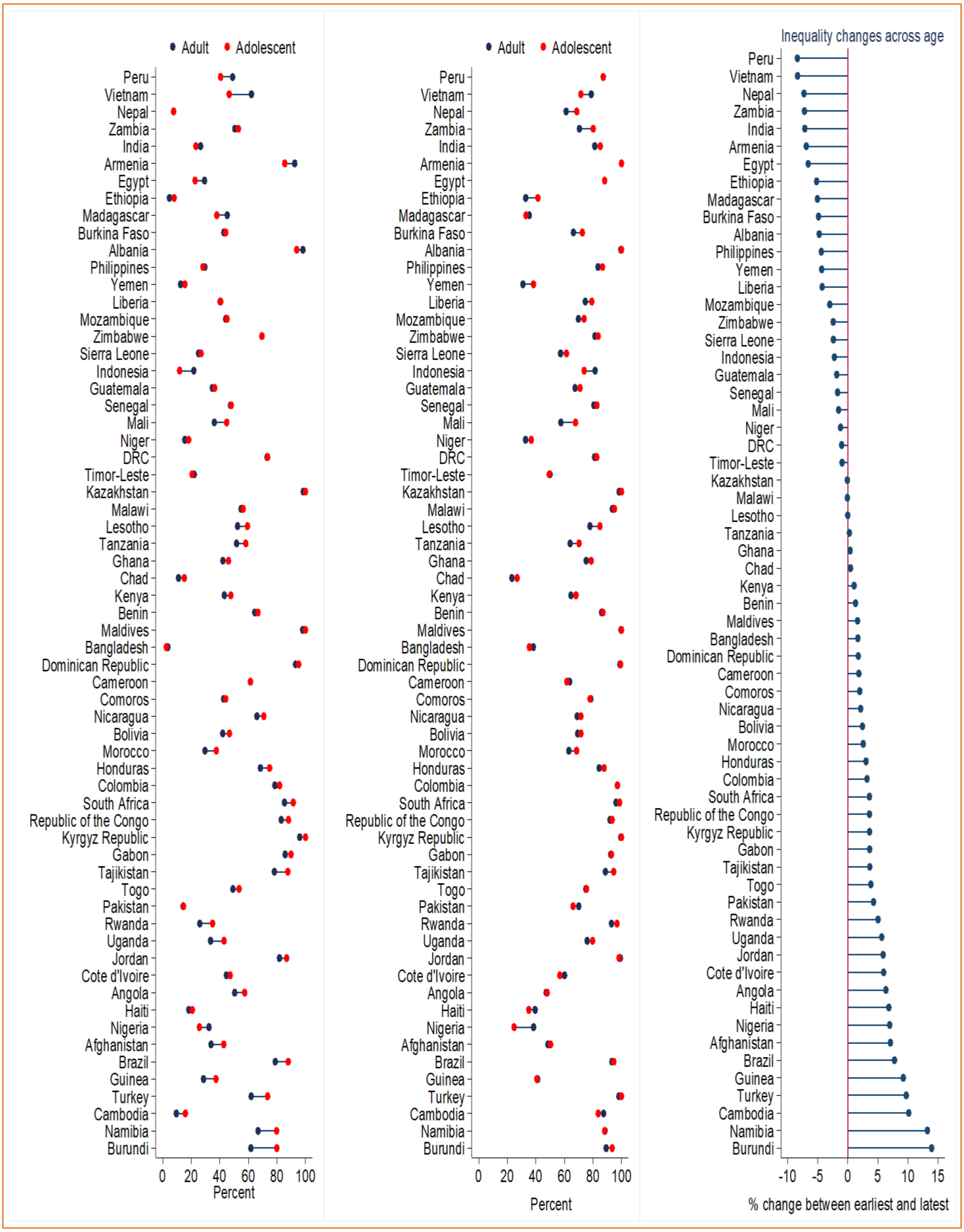
Adult
Adolescent
Peru
Vietnam
Nepal
Zambia
India
Armenia
Egypt
Ethiopia
Madagascar
Burkina Faso
Albania
Philippines
Yemen
Liberia
Mozambique
Zimbabwe
Sierra Leone
Indonesia
Guatemala
Senegal
Mali
Niger
DRC
Timor-Leste
Kazakhstan
Malawi
Lesotho
Tanzania
Ghana
Chad
Kenya
Benin
Maldives
Bangladesh
Dominican Republic
Cameroon
Comoros
Nicaragua
Bolivia
Morocco
Honduras
Colombia
South Africa
Republic of the Congo
Kyrgyz Republic
Gabon
Tajikistan
Togo
Pakistan
Rwanda
Uganda
Jordan
Cote d'Ivoire
Angola
Haiti
Nigeria
Afghanistan
Brazil
Guinea
Turkey
Cambodia
Namibia
Burundi
0 20 40 60 80 100
Percent
Adult
Adolescent
Peru
Vietnam
Nepal
Zambia
India
Armenia
Egypt
Ethiopia
Madagascar
Burkina Faso
Albania
Philippines
Yemen
Liberia
Mozambique
Zimbabwe
Sierra Leone
Indonesia
Guatemala
Senegal
Mali
Niger
DRC
Timor-Leste
Kazakhstan
Malawi
Lesotho
Tanzania
Ghana
Chad
Kenya
Benin
Maldives
Bangladesh
Dominican Republic
Cameroon
Comoros
Nicaragua
Bolivia
Morocco
Honduras
Colombia
South Africa
Republic of the Congo
Kyrgyz Republic
Gabon
Tajikistan
Togo
Pakistan
Rwanda
Uganda
Jordan
Cote d'Ivoire
Angola
Haiti
Nigeria
Afghanistan
Brazil
Guinea
Turkey
Cambodia
Namibia
Burundi
0 20 40 60 80 100
Percent
Inequality changes across age
Peru
Vietnam
Nepal
Zambia
India
Armenia
Egypt
Ethiopia
Madagascar
Burkina Faso
Albania
Philippines
Yemen
Liberia
Mozambique
Zimbabwe
Sierra Leone
Indonesia
Guatemala
Senegal
Mali
Niger
DRC
Timor-Leste
Kazakhstan
Malawi
Lesotho
Tanzania
Ghana
Chad
Kenya
Benin
Maldives
Bangladesh
Dominican Republic
Cameroon
Comoros
Nicaragua
Bolivia
Morocco
Honduras
Colombia
South Africa
Republic of the Congo
Kyrgyz Republic
Gabon
Tajikistan
Togo
Pakistan
Rwanda
Uganda
Jordan
Cote d'Ivoire
Angola
Haiti
Nigeria
Afghanistan
Brazil
Guinea
Turkey
Cambodia
Namibia
Burundi
-10 -5 0 5 10 15
% change between earliest and latest

Table S11: Changes of relative inequalities in the utilization of institutional delivery services between earliest and latest DHS rounds by wealth quintiles

Country	Earliest survey year	Latest survey year	Earliest		Latest		Rate ratio (Q5/Q1)		Difference in rate ratio between earliest and latest
			Poorest, Q1	Richest, Q5	Poorest, Q1	Richest, Q5	Earliest	Latest	
Afghanistan	2010	2015	11.7	65.1	22.4	83.2	5.6	3.7	-1.8
Albania	2009	2018	94.3	99.6	99.7	100.0	1.1	1.0	-0.1
Angola	2007	2016	14.3	94.1	13.7	86.9	6.6	6.4	-0.2
Armenia	2000	2016	84.5	99.7	100.0	100.0	1.2	1.0	-0.2
Bangladesh	1994	2014	0.4	16.2	14.9	71.2	45.8	4.8	-41.1
Benin	1996	2018	34.5	96.6	65.4	99.7	2.8	1.5	-1.3
Bolivia	1994	2008	7.9	94.1	32.4	99.0	11.9	3.1	-8.8
Burkina Faso	1993	2010	28.3	88.5	46.4	93.5	3.1	2.0	-1.1
Burundi	2010	2017	53.3	80.9	82.6	97.1	1.5	1.2	-0.3
Cambodia	2000	2014	1.5	47.2	72.9	97.4	31.2	1.3	-29.9
Cameroon	1991	2011	29.0	94.3	16.6	96.7	3.2	5.8	2.6
Chad	1997	2015	2.2	34.9	13.7	60.7	16.2	4.4	-11.8
Colombia	1990	2015	49.1	97.7	89.4	100.0	2.0	1.1	-0.9
Comoros	1996	2012	19.7	76.6	59.3	93.4	3.9	1.6	-2.3
Republic of the Congo	2005	2012	66.7	98.7	76.8	99.1	1.5	1.3	-0.2
Democratic Republic of the Congo	2007	2014	58.6	97.5	66.7	98.6	1.7	1.5	-0.2
Cote d'Ivoire	1994	2012	16.1	83.1	34.8	94.3	5.2	2.7	-2.5
Dominican Republic	1996	2013	90.4	98.5	96.4	100.0	1.1	1.0	-0.1
Egypt	1995	2014	13.8	73.2	78.0	97.4	5.3	1.2	-4.0
Ethiopia	2000	2016	0.8	22.9	13.9	77.5	29.6	5.6	-24.0
Gabon	2000	2012	63.4	98.4	77.3	99.2	1.6	1.3	-0.3
Ghana	1993	2014	23.8	84.2	49.8	98.0	3.5	2.0	-1.6
Guatemala	1995	2015	9.7	92.1	39.5	96.5	9.5	2.4	-7.1
Guinea	1999	2012	10.1	68.3	18.6	81.0	6.7	4.3	-2.4
Haiti	1995	2017	2.7	59.8	14.1	79.9	22.3	5.7	-16.7
Honduras	2006	2012	37.1	97.5	61.3	98.4	2.6	1.6	-1.0
India	1993	2016	6.6	71.2	63.0	96.2	10.8	1.5	-9.3
Indonesia	1997	2017	4.0	54.1	54.7	96.9	13.5	1.8	-11.7
Jordan	1990	2018	67.8	89.5	97.7	99.9	1.3	1.0	-0.3
Kazakhstan	1995	1999	97.0	100.0	95.5	100.0	1.0	1.0	0.0
Kenya	1993	2014	21.4	75.1	33.7	94.1	3.5	2.8	-0.7
Kyrgyz Republic	1997	2012	90.6	99.3	99.7	100.0	1.1	1.0	-0.1
Lesotho	2004	2014	32.5	81.4	61.5	93.8	2.5	1.5	-1.0
Liberia	2007	2016	20.4	71.5	64.0	77.9	3.5	1.2	-2.3
Madagascar	1997	2009	25.4	44.4	18.1	67.5	1.7	3.7	2.0
Malawi	1992	2016	42.6	76.1	91.0	97.5	1.8	1.1	-0.7
Maldives	2009	2017	94.8	100.0	99.9	98.4	1.1	1.0	-0.1
Mali	1996	2013	10.2	80.0	30.8	94.4	7.8	3.1	-4.8
Morocco	1992	2004	4.7	73.9	31.2	94.0	15.8	3.0	-12.8
Mozambique	1997	2015	17.1	83.0	52.0	96.5	4.9	1.9	-3.0
Namibia	1992	2013	50.4	93.1	74.4	98.5	1.8	1.3	-0.5
Nepal	1996	2016	1.8	30.3	38.6	89.9	17.3	2.3	-15.0
Nicaragua	1998	2001	35.9	92.3	30.4	97.7	2.6	3.2	0.6
Niger	1998	2012	4.1	64.9	15.5	73.2	15.8	4.7	-11.1
Nigeria	2003	2013	11.8	82.0	6.4	81.0	6.9	12.6	5.7
Pakistan	1991	2018	2.2	47.9	46.5	92.9	21.9	2.0	-19.9
Peru	1992	2012	13.8	92.2	60.6	99.4	6.7	1.6	-5.0
Philippines	1993	2017	7.6	72.6	64.7	98.3	9.5	1.5	-8.0
Rwanda	1992	2015	20.9	44.6	88.1	97.9	2.1	1.1	-1.0
Senegal	1997	2017	23.8	82.9	56.9	97.2	3.5	1.7	-1.8
Sierra Leone	2008	2013	18.2	40.6	51.2	73.7	2.2	1.4	-0.8
South Africa	1998	2016	70.7	99.2	92.5	99.1	1.4	1.1	-0.3
Tajikistan	2012	2017	59.7	92.0	79.0	95.8	1.5	1.2	-0.3
Tanzania	1996	2016	26.9	80.5	43.7	95.6	3.0	2.2	-0.8
Timor-Leste	2010	2016	5.2	60.2	18.2	88.4	11.5	4.9	-6.6
Togo	1998	2014	24.4	91.0	45.4	97.8	3.7	2.2	-1.6
Turkey	1993	2013	30.7	94.4	93.5	100.0	3.1	1.1	-2.0
Uganda	1995	2016	19.8	66.1	67.3	93.4	3.3	1.4	-2.0
Vietnam	1997	2002	34.8	92.9	48.0	98.9	2.7	2.1	-0.6
Zambia	1992	2014	18.8	91.3	54.2	95.9	4.9	1.8	-3.1
Zimbabwe	1994	2015	54.6	92.7	67.7	97.3	1.7	1.4	-0.3

Supplement to: Hasan MM, Magalhaes RJS, Fatima Y, Ahmed S, Mamun AA. Levels, trends and inequalities in using institutional delivery services in low- and middle-income countries: a stratified analysis by facility type. *Glob Health Sci Pract*. 2021;9(1). https://doi.org/10.9745/GHSP-D-20-00533

Table S12: Changes of relative inequalities in the utilization of institutional delivery services between earliest and latest DHS rounds by place of residence

Country	Earliest survey year	Latest survey year	Earliest		Latest		Rate ratio (Q5/Q1)		Difference in rate ratio between earliest and latest
			Rural	Urban	Rural	Urban	Earliest	Latest	
Afghanistan	2010	2015	26.5	67.7	40.3	76.5	2.6	1.9	-0.7
Albania	2009	2018	97.1	99.6	99.3	99.8	1.0	1.0	0.0
Angola	2007	2016	22.4	80.4	18.4	66.3	3.6	3.6	0.0
Armenia	2000	2016	85.7	98.1	100.0	100.0	1.1	1.0	-0.1
Bangladesh	1994	2014	1.7	20.1	30.7	57.6	12.2	1.9	-10.3
Benin	1996	2018	57.5	80.1	82.8	91.3	1.4	1.1	-0.3
Bolivia	1994	2008	21.0	61.8	45.9	89.4	2.9	1.9	-1.0
Brazil	1991	1996	67.8	90.4	81.3	97.4	1.3	1.2	-0.1
Burkina Faso	1993	2010	34.8	92.0	61.1	94.2	2.6	1.5	-1.1
Burundi	2010	2017	59.8	87.8	88.5	96.7	1.5	1.1	-0.4
Cambodia	2000	2014	6.6	32.0	85.5	97.5	4.9	1.1	-3.7
Cameroon	1991	2011	48.0	82.2	46.2	86.6	1.7	1.9	0.2
Chad	1997	2015	5.2	34.4	15.5	56.9	6.7	3.7	-3.0
Colombia	1990	2015	60.1	87.8	90.6	99.6	1.5	1.1	-0.4
Comoros	1996	2012	34.7	68.9	74.3	89.6	2.0	1.2	-0.8
Republic of the Congo	2005	2012	73.0	96.2	83.0	97.7	1.3	1.2	-0.1
Democratic Republic of the Congo	2007	2014	62.2	90.0	75.3	94.2	1.4	1.3	-0.2
Cote d'Ivoire	1994	2012	29.2	75.8	45.3	83.2	2.6	1.8	-0.8
Dominican Republic	1991	2013	85.7	98.7	97.6	99.5	1.2	1.0	-0.1
Egypt	1992	2014	16.0	51.2	85.6	94.1	3.2	1.1	-2.1
Ethiopia	2000	2016	1.8	32.5	26.3	86.3	17.6	3.3	-14.3
Gabon	2000	2012	67.9	93.5	71.2	96.5	1.4	1.4	0.0
Ghana	1993	2014	28.2	79.2	61.7	91.8	2.8	1.5	-1.3
Guatemala	1995	2015	21.3	64.0	58.2	85.9	3.0	1.5	-1.5
Guinea	1999	2012	18.8	61.5	29.8	71.6	3.3	2.4	-0.9
Haiti	1995	2017	8.0	41.0	29.0	59.1	5.1	2.0	-3.1
Honduras	2006	2012	53.7	90.3	76.0	95.2	1.7	1.3	-0.4
India	1993	2016	16.7	58.5	78.2	89.9	3.5	1.2	-2.4
Indonesia	1991	2017	9.1	50.6	70.7	92.4	5.6	1.3	-4.3
Jordan	1990	2018	75.8	84.7	99.8	99.1	1.1	1.0	-0.1
Kazakhstan	1995	1999	97.5	99.9	97.3	100.0	1.0	1.0	0.0
Kenya	1993	2014	38.6	77.2	53.9	84.7	2.0	1.6	-0.4
Kyrgyz Republic	1997	2012	95.4	98.7	99.4	100.0	1.0	1.0	0.0
Lesotho	2004	2014	48.0	86.8	75.1	88.4	1.8	1.2	-0.6
Liberia	2007	2016	27.3	68.4	71.6	78.2	2.5	1.1	-1.4
Madagascar	1992	2009	43.1	53.3	32.0	61.2	1.2	1.9	0.7
Malawi	1992	2016	51.1	86.7	93.2	97.2	1.7	1.0	-0.7
Maldives	2009	2017	97.2	100.0	99.9	99.2	1.0	1.0	0.0
Mali	1996	2013	22.9	78.3	50.0	92.8	3.4	1.9	-1.6
Morocco	1992	2004	14.5	61.6	41.7	84.5	4.3	2.0	-2.2
Mozambique	1997	2015	33.7	82.5	63.6	91.0	2.4	1.4	-1.0
Namibia	1992	2013	58.9	87.2	81.6	96.0	1.5	1.2	-0.3
Nepal	1996	2016	5.1	43.8	49.7	72.6	8.5	1.5	-7.1
Nicaragua	1998	2001	45.2	86.3	47.8	90.7	1.9	1.9	0.0
Niger	1992	2012	5.2	73.2	25.0	85.2	14.1	3.4	-10.7
Nigeria	1990	2013	23.8	62.4	23.3	63.3	2.6	2.7	0.1
Pakistan	1991	2018	5.9	33.9	63.9	82.5	5.8	1.3	-4.5
Peru	1992	2012	15.5	70.2	68.9	96.4	4.5	1.4	-3.1
Philippines	1993	2017	14.1	45.5	78.7	90.0	3.2	1.1	-2.1
Rwanda	1992	2015	24.2	66.6	92.2	97.5	2.7	1.1	-1.7
Senegal	1993	2017	29.5	81.7	72.8	94.6	2.8	1.3	-1.5
Sierra Leone	2008	2013	20.1	39.2	53.3	71.2	2.0	1.3	-0.6
South Africa	1998	2016	77.6	94.7	95.6	96.8	1.2	1.0	-0.2
Tajikistan	2012	2017	75.4	89.7	87.4	95.1	1.2	1.1	-0.1
Tanzania	1992	2016	43.6	85.3	56.0	87.4	2.0	1.6	-0.4
Timor-Leste	2010	2016	12.4	53.1	36.1	85.0	4.3	2.4	-1.9
Togo	1998	2014	38.6	85.8	63.9	95.0	2.2	1.5	-0.7
Turkey	1993	2013	44.1	75.7	95.0	99.1	1.7	1.0	-0.7
Uganda	1995	2016	29.3	76.4	72.8	89.4	2.6	1.2	-1.4
Vietnam	1997	2002	56.6	92.0	74.3	99.3	1.6	1.3	-0.3
Yemen	1992	2013	8.3	35.6	24.2	51.4	4.3	2.1	-2.1
Zambia	1992	2014	25.9	78.7	61.3	91.1	3.0	1.5	-1.6
Zimbabwe	1994	2015	61.8	91.5	76.1	93.9	1.5	1.2	-0.2

Supplement to: Hasan MM, Magalhaes RJS, Fatima Y, Ahmed S, Mamun AA. Levels, trends and inequalities in using institutional delivery services in low- and middle-income countries: a stratified analysis by facility type. *Glob Health Sci Pract*. 2021;9(1). https://doi.org/10.9745/GHSP-D-20-00533

Table S13: Changes of relative inequalities in the utilization of institutional delivery services between earliest and latest DHS rounds by education

Country	Earliest survey year	Latest survey year	Earliest		Latest		Rate ratio (Q5/Q1)		Difference in rate ratio between earliest and latest
			Below Secondary	Secondary+	Below Secondary	Secondary+	Earliest	Latest	
Afghanistan	2010	2015	56.4	77.3	45.7	79.8	1.4	1.7	0.4
Albania	2009	2018	97.2	99.8	99.1	100.0	1.0	1.0	0.0
Angola	2007	2016	47.4	95.6	32.1	78.8	2.0	2.5	0.4
Armenia	2000	2016	66.7	92.0	100.0	100.0	1.4	1.0	-0.4
Bangladesh	1994	2014	1.2	16.0	22.6	48.8	13.3	2.2	-11.1
Benin	1996	2018	63.0	95.6	83.5	97.7	1.5	1.2	-0.3
Bolivia	1994	2008	25.3	71.3	54.6	89.9	2.8	1.6	-1.2
Brazil	1991	1996	77.2	98.0	87.6	98.3	1.3	1.1	-0.1
Burkina Faso	1993	2010	41.5	90.6	65.0	96.8	2.2	1.5	-0.7
Burundi	2010	2017	60.2	91.3	88.3	96.9	1.5	1.1	-0.4
Cambodia	2000	2014	6.0	33.3	83.4	94.1	5.6	1.1	-4.4
Cameroon	1991	2011	54.1	88.6	48.6	92.5	1.6	1.9	0.3
Chad	1997	2015	10.2	54.3	19.4	57.1	5.3	3.0	-2.4
Colombia	1990	2015	67.4	91.7	88.1	98.9	1.4	1.1	-0.2
Comoros	1996	2012	37.5	74.2	72.7	91.2	2.0	1.3	-0.7
Republic of the Congo	2005	2012	76.1	89.9	83.9	97.1	1.2	1.2	0.0
Democratic Republic of the Congo	2007	2014	65.6	87.6	74.6	91.6	1.3	1.2	-0.1
Cote d'Ivoire	1994	2012	41.5	81.8	57.0	82.2	2.0	1.4	-0.5
Dominican Republic	1991	2013	89.9	98.8	97.5	99.8	1.1	1.0	-0.1
Egypt	1992	2014	19.6	53.7	77.8	91.6	2.7	1.2	-1.6
Ethiopia	2000	2016	2.8	43.5	28.7	84.6	15.7	2.9	-12.7
Gabon	2000	2012	79.9	92.7	85.3	96.1	1.2	1.1	0.0
Ghana	1993	2014	40.1	83.6	61.1	87.8	2.1	1.4	-0.7
Guatemala	1995	2015	27.2	89.0	57.3	92.0	3.3	1.6	-1.7
Guinea	1999	2012	27.1	71.6	37.1	75.5	2.6	2.0	-0.6
Haiti	1995	2017	12.9	60.6	23.7	59.9	4.7	2.5	-2.2
Honduras	2006	2012	61.8	94.9	77.7	96.7	1.5	1.2	-0.3
India	1993	2016	16.2	64.0	68.3	90.8	4.0	1.3	-2.6
Indonesia	1991	2017	12.1	50.5	65.0	86.8	4.2	1.3	-2.8
Jordan	1990	2018	73.3	87.3	94.5	99.6	1.2	1.1	-0.1
Kazakhstan	1995	1999	100.0	98.5	100.0	98.4	1.0	1.0	0.0
Kenya	1993	2014	35.7	71.4	53.8	86.6	2.0	1.6	-0.4
Kyrgyz Republic	1997	2012	100.0	96.1	100.0	99.6	1.0	1.0	0.0
Lesotho	2004	2014	44.2	71.0	66.6	88.2	1.6	1.3	-0.3
Liberia	2007	2016	34.8	67.8	70.7	82.5	1.9	1.2	-0.8
Madagascar	1992	2009	42.2	52.3	29.2	59.5	1.2	2.0	0.8
Malawi	1992	2016	53.8	96.1	92.9	97.1	1.8	1.0	-0.7
Maldives	2009	2017	96.0	99.7	100.0	99.6	1.0	1.0	0.0
Mali	1996	2013	34.9	88.6	55.5	90.0	2.5	1.6	-0.9
Morocco	1992	2004	24.6	85.2	56.3	92.7	3.5	1.6	-1.8
Mozambique	1997	2015	42.6	93.0	66.2	93.4	2.2	1.4	-0.8
Namibia	1992	2013	58.0	88.5	72.0	94.8	1.5	1.3	-0.2
Nepal	1996	2016	4.7	36.8	45.9	78.3	7.8	1.7	-6.1
Nicaragua	1998	2001	56.0	89.6	56.2	93.4	1.6	1.7	0.1
Niger	1992	2012	14.6	78.9	31.0	76.3	5.4	2.5	-3.0
Nigeria	1990	2013	25.0	80.1	20.6	70.9	3.2	3.4	0.2
Pakistan	1991	2018	8.6	53.2	60.1	88.0	6.2	1.5	-4.7
Peru	1992	2012	22.2	74.7	67.6	95.4	3.4	1.4	-2.0
Philippines	1993	2017	13.2	41.7	56.6	89.8	3.2	1.6	-1.6
Rwanda	1992	2015	24.1	66.7	92.2	98.5	2.8	1.1	-1.7
Senegal	1993	2017	44.9	92.0	77.7	94.6	2.0	1.2	-0.8
Sierra Leone	2008	2013	22.5	45.6	54.2	73.8	2.0	1.4	-0.7
South Africa	1998	2016	75.4	91.9	94.1	96.6	1.2	1.0	-0.2
Tajikistan	2012	2017	68.3	79.4	83.2	89.4	1.2	1.1	-0.1
Tanzania	1992	2016	51.4	83.8	59.8	90.3	1.6	1.5	-0.1
Timor-Leste	2010	2016	11.0	40.0	29.6	64.4	3.6	2.2	-1.5
Togo	1998	2014	46.0	87.5	68.8	95.0	1.9	1.4	-0.5
Turkey	1993	2013	58.2	86.8	96.6	99.8	1.5	1.0	-0.5
Uganda	1995	2016	30.7	69.2	70.7	90.1	2.3	1.3	-1.0
Vietnam	1997	2002	41.7	72.8	61.9	87.4	1.7	1.4	-0.3
Yemen	1992	2013	11.7	75.3	28.4	50.9	6.4	1.8	-4.6

Zambia	1992	2014	43.1	82.4	63.5	86.9	1.9	1.4	-0.5
Zimbabwe	1994	2015	58.6	87.8	68.7	87.8	1.5	1.3	-0.2

Table S14: Changes of relative inequalities in the utilization of institutional delivery services between earliest and latest DHS rounds by age

Country	Earliest survey year	Latest survey year	Earliest		Latest		Rate ratio (Q5/Q1)		Difference in rate ratio between earliest and latest
			Adolescent	Adult	Adolescent	Adult	Earliest	Latest	
Afghanistan	2010	2015	42.8	33.9	50.3	48.5	0.8	1.0	0.2
Albania	2009	2018	93.9	98.2	100.0	99.6	1.0	1.0	-0.1
Angola	2007	2016	57.4	50.5	47.9	47.3	0.9	1.0	0.1
Armenia	2000	2016	85.6	92.5	100.0	100.0	1.1	1.0	-0.1
Bangladesh	1994	2014	2.6	3.7	35.5	38.2	1.4	1.1	-0.3
Benin	1996	2018	66.7	64.5	87.0	86.1	1.0	1.0	0.0
Bolivia	1994	2008	46.7	42.1	71.6	69.4	0.9	1.0	0.1
Brazil	1991	1996	88.0	78.9	94.6	93.3	0.9	1.0	0.1
Burkina Faso	1993	2010	44.2	42.8	72.6	66.4	1.0	0.9	-0.1
Burundi	2010	2017	80.0	61.8	93.5	89.2	0.8	1.0	0.2
Cambodia	2000	2014	15.9	9.6	83.7	87.5	0.6	1.0	0.4
Cameroon	1991	2011	61.4	61.5	61.7	63.7	1.0	1.0	0.0
Chad	1997	2015	15.2	11.1	26.9	23.3	0.7	0.9	0.1
Colombia	1990	2015	81.9	78.6	97.3	97.1	1.0	1.0	0.0
Comoros	1996	2012	44.1	42.7	77.9	78.5	1.0	1.0	0.0
Republic of the Congo	2005	2012	88.1	83.1	93.4	92.0	0.9	1.0	0.0
Democratic Republic of the Congo	2007	2014	73.5	73.1	82.7	81.2	1.0	1.0	0.0
Cote d'Ivoire	1994	2012	47.3	44.7	56.8	60.1	0.9	1.1	0.1
Dominican Republic	1991	2013	95.3	93.1	99.4	98.9	1.0	1.0	0.0
Egypt	1992	2014	22.7	29.3	88.2	88.2	1.3	1.0	-0.3
Ethiopia	2000	2016	8.1	4.7	41.4	32.9	0.6	0.8	0.2
Gabon	2000	2012	89.9	85.8	93.0	92.5	1.0	1.0	0.0
Ghana	1993	2014	46.1	42.2	78.8	75.3	0.9	1.0	0.0
Guatemala	1995	2015	36.4	34.8	71.0	67.5	1.0	1.0	0.0
Guinea	1999	2012	37.3	28.6	40.8	41.3	0.8	1.0	0.2
Haiti	1995	2017	20.8	18.3	35.1	39.5	0.9	1.1	0.2
Honduras	2006	2012	74.9	68.5	87.8	84.4	0.9	1.0	0.0
India	1993	2016	23.3	26.6	85.1	81.3	1.1	1.0	-0.2
Indonesia	1991	2017	11.9	21.8	73.8	81.5	1.8	1.1	-0.7
Jordan	1990	2018	86.8	81.8	98.3	99.2	0.9	1.0	0.1
Kazakhstan	1995	1999	100.0	98.4	100.0	98.3	1.0	1.0	0.0
Kenya	1993	2014	47.8	43.2	68.1	64.6	0.9	0.9	0.0
Kyrgyz Republic	1997	2012	100.0	95.9	100.0	99.6	1.0	1.0	0.0
Lesotho	2004	2014	59.4	52.5	84.9	77.9	0.9	0.9	0.0
Liberia	2007	2016	40.7	40.3	79.2	74.6	1.0	0.9	0.0
Madagascar	1992	2009	37.9	45.2	33.1	35.4	1.2	1.1	-0.1
Malawi	1992	2016	56.4	55.0	95.0	93.6	1.0	1.0	0.0
Maldives	2009	2017	100.0	98.1	100.0	99.7	1.0	1.0	0.0
Mali	1996	2013	44.8	36.2	67.8	57.6	0.8	0.8	0.0
Morocco	1992	2004	37.6	29.7	68.6	63.2	0.8	0.9	0.1
Mozambique	1997	2015	45.1	44.1	73.8	69.9	1.0	0.9	0.0
Namibia	1992	2013	79.8	66.8	88.2	88.5	0.8	1.0	0.2
Nepal	1996	2016	7.7	7.6	68.7	61.2	1.0	0.9	-0.1
Nicaragua	1998	2001	70.8	66.1	71.6	68.9	0.9	1.0	0.0
Niger	1992	2012	18.3	15.5	36.8	32.8	0.8	0.9	0.0
Nigeria	1990	2013	25.8	32.4	24.8	38.4	1.3	1.5	0.3
Pakistan	1991	2018	14.6	14.3	66.1	70.1	1.0	1.1	0.1
Peru	1992	2012	40.6	49.0	87.2	87.2	1.2	1.0	-0.2
Philippines	1993	2017	28.2	29.5	86.7	83.5	1.0	1.0	-0.1
Rwanda	1992	2015	35.0	26.0	96.9	93.0	0.7	1.0	0.2
Senegal	1993	2017	48.0	47.5	82.8	80.7	1.0	1.0	0.0
Sierra Leone	2008	2013	26.9	25.2	61.5	57.4	0.9	0.9	0.0
South Africa	1998	2016	91.5	85.4	98.7	96.1	0.9	1.0	0.0
Tajikistan	2012	2017	87.6	78.2	94.6	88.8	0.9	0.9	0.0
Tanzania	1992	2016	58.2	51.7	70.3	64.1	0.9	0.9	0.0
Timor-Leste	2010	2016	20.6	22.2	49.4	50.0	1.1	1.0	-0.1
Togo	1998	2014	53.5	49.2	75.5	75.1	0.9	1.0	0.1
Turkey	1993	2013	73.5	62.0	100.0	98.2	0.8	1.0	0.1

Uganda	1995	2016	43.0	33.5	79.8	76.0	0.8	1.0	0.2
Vietnam	1997	2002	46.6	62.2	71.6	78.8	1.3	1.1	-0.2
Yemen	1992	2013	15.6	12.6	38.4	31.0	0.8	0.8	0.0
Zambia	1992	2014	53.0	50.6	80.2	70.5	1.0	0.9	-0.1
Zimbabwe	1994	2015	69.4	69.6	83.7	81.4	1.0	1.0	0.0

Table S15: Change rates in the utilization of delivery services in public and private facilities in low- and middle-income countries

Country	Delivery in public facilities				Delivery in private facilities			
	**1990-1999**	**2000-2009**	**2010-2018**	**1990-2018**	**1990-1999**	**2000-2009**	**2010-2018**	**1990-2018**
Afghanistan	4.9	7.9	7.6	6.8	-4.6	-4.6	-0.3	-3.3
Albania	0.0	-0.1	-0.3	-0.2	27.9	30.8	32.1	30.2
Angola	-0.8	-1.2	-1.4	-1.1	-1.5	1.1	3.3	0.9
Armenia	0.2	0.1	0.1	0.1	25.0	26.5	25.9	25.8
Bangladesh	9.4	9.2	8.4	9.0	14.8	14.3	12.0	13.8
Benin	1.4	1.2	0.9	1.2	1.4	1.5	1.6	1.5
Bolivia	5.4	3.9	2.3	3.9	-2.4	-1.9	-1.3	-1.9
Brazil	1.6	0.5	0.1	0.7	16.0	9.4	3.1	9.7
Burkina Faso	3.7	3.0	2.0	2.9	2.0	2.4	2.8	2.4
Burundi	15.0	13.8	5.4	11.8	-6.6	-7.5	-5.8	-6.7
Cambodia	22.5	18.8	7.6	16.7	15.6	15.6	13.6	15.0
Cameroon	-0.8	-0.8	-0.8	-0.8	3.0	2.9	2.7	2.9
Chad	4.9	4.9	4.6	4.8	-1.0	-0.1	0.6	-0.2
Colombia	1.4	1.0	0.6	1.0	-5.7	-5.4	-4.8	-5.3
Comoros	5.3	3.5	1.9	3.6	20.0	20.5	20.3	20.3
Republic of the Congo	0.8	0.6	0.2	0.6	4.5	7.4	8.2	6.7
Democratic Republic of the Congo	3.8	3.8	2.6	3.4	-3.2	-4.0	-3.1	-3.5
Cote d'Ivoire	1.5	1.3	1.1	1.3	10.4	10.9	11.0	10.7
Dominican Republic	0.3	0.3	0.2	0.3	-0.1	0.0	0.1	0.0
Egypt	1.8	1.8	1.7	1.8	9.9	7.3	4.1	7.2
Ethiopia	12.3	12.6	11.1	12.1	11.9	12.6	13.2	12.6
Gabon	-0.3	-0.4	-0.5	-0.4	3.3	3.8	3.7	3.6
Ghana	4.7	3.8	2.6	3.7	-1.2	-1.2	-1.1	-1.2
Guatemala	4.5	3.6	2.6	3.6	1.5	1.7	1.9	1.7
Guinea	1.6	1.7	1.7	1.7	11.1	12.0	12.2	11.8
Haiti	5.9	5.5	4.8	5.4	-1.9	-1.7	-1.4	-1.7
Honduras	7.0	5.5	2.1	5.0	-4.8	-3.2	0.0	-2.8
India	6.8	6.1	4.6	5.9	3.8	3.6	3.3	3.6
Indonesia	5.1	4.9	4.5	4.8	8.0	6.7	4.7	6.6
Jordan	0.5	0.4	0.4	0.4	0.9	0.9	0.9	0.9
Kazakhstan	0.0	-0.2	-0.5	-0.2	1.3	1.2	1.2	1.2
Kenya	1.8	1.7	1.6	1.7	1.8	1.9	2.0	1.9
Kyrgyz Republic	0.6	0.2	0.1	0.3	-1.2	-11.7	16.4	-2.4
Lesotho	6.2	5.0	2.7	4.7	4.4	6.7	8.4	6.4
Liberia	10.3	9.8	6.5	9.0	2.1	3.2	4.0	3.1
Madagascar	-1.7	-1.8	-1.7	-1.7	5.1	5.5	5.7	5.4
Malawi	4.8	3.5	2.2	3.5	-0.3	0.0	0.2	-0.1
Maldives	-0.3	-0.6	-1.3	-0.7	4.3	6.2	7.2	5.9
Mali	5.6	4.7	3.3	4.6	10.0	10.6	10.9	10.5
Morocco	7.0	4.4	2.1	4.6	5.0	5.6	5.5	5.4
Mozambique	3.2	2.6	1.9	2.6	5.7	6.3	7.0	6.3
Namibia	1.5	1.1	0.7	1.1	4.2	4.5	4.7	4.5
Nepal	13.2	11.9	8.5	11.3	12.7	12.8	12.0	12.5
Nicaragua	0.2	-0.7	-0.6	-0.4	12.6	19.9	6.7	13.5
Niger	3.4	3.3	3.0	3.3	2.2	2.7	3.2	2.7
Nigeria	-1.4	-1.3	-1.2	-1.3	9.4	9.3	8.0	8.9
Pakistan	4.0	4.0	3.8	3.9	8.4	7.4	5.8	7.3
Peru	5.3	3.3	1.7	3.5	-0.1	0.0	0.1	0.0
Philippines	6.1	5.2	4.0	5.1	3.2	3.1	2.9	3.1
Rwanda	11.4	7.4	3.0	7.4	-4.4	-4.0	-3.6	-4.0
Senegal	3.3	2.5	1.7	2.5	0.6	0.6	0.7	0.6
Sierra Leone	16.1	22.1	10.9	16.8	-9.2	-9.7	-4.8	-8.1
South Africa	1.2	0.8	0.5	0.9	-0.7	-0.4	-0.1	-0.4
Tajikistan	6.4	6.2	2.8	5.3	-0.4	-2.5	44.7	11.5
Tanzania	0.8	0.8	0.8	0.8	3.1	3.2	3.3	3.2
Timor-Leste	11.4	15.4	12.3	13.2	0.0	-0.1	-6.2	-1.8
Togo	2.4	2.1	1.6	2.1	6.0	6.4	6.4	6.3
Turkey	0.3	0.3	0.2	0.3	11.4	10.2	7.4	9.8
Uganda	6.9	5.8	4.1	5.7	0.4	0.5	0.6	0.5
Vietnam	6.3	2.5	0.7	3.2	13.0	17.7	11.7	14.4
Yemen	2.5	2.6	2.6	2.6	10.9	10.8	10.0	10.6
Zambia	3.1	2.6	2.0	2.6	-4.1	-4.1	-4.0	-4.0
Zimbabwe	0.6	0.6	0.5	0.6	-1.2	-1.1	-1.0	-1.1

Table S16: Trends in the utilization (%, 95% credible intervals) of delivery services in public facilities in low- and middle-income countries by wealth quintiles

	Poorest				Richest			
**Country**	**1990**	**2000**	**2010**	**2018**	**1990**	**2000**	**2010**	**2018**
Afghanistan	2.2 (0-17.2)	3.8 (0.3-16.8)	11.1 (5.9-17.4)	30.3 (13.5-51.7)	10.3 (0.1-72.3)	20.8 (2.5-67.1)	49.9 (36.1-63.6)	76.8 (55-90.3)
Albania	16.1 (1.7-56.6)	62.7 (33.8-86.4)	95.5 (92.7-97.2)	99.4 (98.9-99.6)	100 (100-100)	99.9 (99.8-100)	98.5 (97.5-99.1)	84.4 (75.5-91.3)
Angola	20.1 (2.6-62.2)	16.5 (5.4-36.5)	14.1 (9.5-19.7)	13.5 (6.4-23.3)	94.6 (78.6-99.5)	91.7 (80.8-97.5)	85.5 (79.6-90.3)	76.5 (61.2-87.3)
Armenia	73.5 (55.7-88.2)	86.3 (80.7-91.1)	93.4 (91.4-95.2)	96.3 (93.9-97.9)	99.9 (99.8-100)	99.5 (99.1-99.8)	97 (95.6-98.1)	87.1 (76.9-94.1)
Bangladesh	0.2 (0.1-0.4)	1.1 (0.8-1.5)	5.7 (4-7.9)	18.9 (11.2-29.6)	10 (5.5-16)	13.2 (10.2-17)	17.8 (12.9-23.6)	22.5 (13.2-34.8)
Benin	31.5 (17.6-46.8)	44.1 (34.6-53.1)	57.7 (49.6-64.9)	67.8 (55.4-78.6)	69.3 (61.1-76.5)	68.4 (63.6-72.5)	67.4 (63.3-70.8)	66.5 (60.7-72.2)
Bolivia	6.1 (3.1-11.3)	16.6 (12.3-21.1)	39 (24.6-53.2)	61 (34.1-80.2)	47.7 (30.7-64.8)	56.8 (48.9-64.2)	65.3 (51.6-77.9)	70.9 (47.5-87.8)
Burkina Faso	20.2 (10.6-34.2)	27.6 (20.7-35.9)	37.4 (24-53)	45.9 (21.9-71.3)	78.6 (64.3-88.7)	82.1 (76.1-86.9)	84.5 (75-91.2)	85.7 (69.6-94.8)
Burundi	3.3 (0.1-16.7)	13.5 (2.6-38.3)	51 (37.3-65.9)	84.4 (74.2-91.9)	11.8 (0.5-55.2)	32.7 (8.2-70.6)	71.6 (59.1-82.2)	90.9 (83.9-95.3)
Cambodia	0 (0-0.1)	1.3 (1-1.8)	33.8 (29-39.2)	90.3 (85.9-93.8)	24 (11.3-41.4)	38.4 (29-48.6)	55.9 (49.6-62.8)	69 (56.5-80)
Cameroon	26.3 (18.5-35.3)	20 (16-23.9)	15 (10.7-20.1)	12 (6.7-19.4)	70.1 (57.9-80.4)	64 (57.3-70.9)	57.1 (45.9-68.6)	51.4 (33.9-68.6)
Chad	0.3 (0.1-0.9)	1.2 (0.7-2.2)	5 (2.6-8.7)	15.1 (5.4-30.4)	24.2 (13.5-37.8)	35.6 (28.3-43.4)	49.2 (40.7-57.2)	60.3 (45.8-72.7)
Colombia	42.5 (30.6-56.2)	60.8 (53.2-68)	76.5 (68.4-83.1)	85.3 (75.9-91.6)	60.7 (38.1-80.8)	70 (56.7-81.2)	77.5 (63-87.3)	81.8 (61.3-93)
Comoros	12 (4.9-24.5)	27.8 (18.7-37.8)	53 (40-65.2)	72.4 (54.4-85.3)	70.2 (48.8-85.5)	79.4 (70.4-86)	86.1 (79.7-91)	89.7 (81.1-95.3)
Republic of the Congo	51.1 (9.1-91)	58.2 (31.9-81.4)	65.6 (55.7-74.4)	69.7 (43.6-88.7)	86.8 (54.1-99.1)	89 (75.7-96.4)	89.3 (84.5-92.8)	87.9 (68.7-96.2)
Democratic Republic of the Congo	35.2 (4.4-84.3)	44.4 (17.9-74.2)	56.5 (45.8-66)	65.6 (42.9-83.4)	31.2 (3.1-84.4)	36.6 (12.9-68.2)	44.8 (34.4-54.9)	52.3 (25.3-75)
Cote d'Ivoire	13.4 (6.8-23.4)	21.3 (15.9-27.1)	32.9 (23.7-43.3)	44 (26.7-63.2)	82.1 (70.6-90.3)	82.3 (76.5-87)	82.1 (74.1-88.5)	81.5 (66-91.4)
Dominican Republic	87 (77.6-92.9)	90.1 (87-92.5)	92.3 (89.2-94.9)	93.6 (88.4-97)	43.2 (29-58.8)	39.4 (32.5-47.8)	35.9 (27-45.7)	33.5 (19.3-50)
Egypt	8.1 (4.4-13.1)	14.2 (11.1-17.7)	24.3 (18.8-30.3)	35.5 (23.4-49.3)	32.5 (25.8-39.7)	28 (25.2-31.2)	24 (21.1-27.6)	21.2 (16.5-27.6)
Ethiopia	0.1 (0-0.2)	0.5 (0.2-0.9)	3.2 (2-4.7)	13.7 (6.2-23.7)	6.1 (2.1-14)	18.4 (11.8-27)	45.6 (37.5-53.8)	70.4 (57.5-82.4)
Gabon	46.5 (21.6-72.2)	58.9 (45.6-71.1)	70.4 (59.2-80.4)	77.6 (59.5-90.6)	71.6 (45.9-89.8)	62.9 (48.9-75.4)	52 (39.3-63.8)	43.3 (22.3-65.9)
Ghana	11.1 (5.5-19.9)	18.9 (13.9-25.1)	31 (21.4-41.3)	43.2 (25.1-61)	57.7 (47.3-68.6)	66.9 (61.4-72.2)	74.8 (69.1-79.7)	80 (71.3-86.7)
Guatemala	4.8 (2.4-8.5)	11.8 (7.8-16.4)	26.9 (18-37.1)	45.1 (27.2-62.8)	60 (48.3-70.8)	60.6 (53.7-66.9)	61 (52.1-69.3)	61.2 (46.8-73.7)
Guinea	6.8 (2.5-14.4)	10.4 (6.9-15.2)	16.6 (11.4-22.9)	23.9 (11.8-40.2)	63.8 (42.2-81.1)	62.3 (52.7-71.4)	60.4 (51-69.4)	58.6 (38.2-74.4)
Haiti	1.2 (0.6-2)	2.8 (2-3.7)	6.7 (5.1-8.7)	13.1 (8.7-19)	23.9 (15-36.4)	35.5 (27.9-43.3)	49.3 (41.3-56.5)	60.6 (47.7-71.1)
Honduras	7.5 (0.3-37.1)	19.6 (5.5-44.2)	52 (40.8-62.3)	77.9 (48.2-93.7)	55.5 (8.4-94.7)	68.9 (38.2-89.5)	80.7 (73.4-86.4)	85.7 (66.6-96.3)
India	2.7 (1.2-5)	9 (6-12.9)	26.5 (17.7-37.1)	50 (31.6-68.8)	24.8 (15.2-35.7)	27 (20.6-33.8)	29.5 (22.4-38)	31.9 (20.7-44.9)
Indonesia	1 (0.4-2.1)	3.8 (2.4-5.8)	13.7 (10.1-17.8)	32.7 (22-45.2)	15 (7.3-25.5)	15.5 (10.8-21.1)	16.5 (12.7-20.5)	17.5 (10.8-25.5)
Jordan	71.8 (58-81.7)	75.1 (68.1-81.1)	77.9 (70.8-83.9)	79.8 (69.6-87.8)	34.1 (22.7-47.2)	31 (24.3-38.4)	28.3 (21.7-35.8)	26.4 (17-37.3)
Kazakhstan	97.7 (92.9-99.6)	94.8 (90-97.7)	81.5 (27.9-99.1)	66.2 (2.9-99.6)	100 (100-100)	100 (100-100)	100 (99.8-100)	99.7 (99.1-100)
Kenya	11.7 (5.8-20)	15.3 (10.7-20.8)	20.5 (13.5-29)	25.8 (13.4-42.1)	47.8 (35.8-60.5)	49.4 (43.2-56.4)	51.1 (43.1-58.5)	52.3 (39.1-65.1)
Kyrgyz Republic	63.3 (42.2-80.9)	95.2 (92.7-97.1)	99.6 (99.3-99.8)	99.9 (99.8-100)	99.4 (98.5-99.8)	99.2 (98.7-99.5)	98.9 (98.2-99.4)	98.6 (96.8-99.4)
Lesotho	9.4 (1.1-35.5)	20.7 (8.4-40.6)	44.5 (34.5-55.9)	66.6 (46.1-83.6)	67.1 (35.5-90)	76.3 (62.5-87.5)	83.3 (78.5-87.4)	87 (76.6-93.7)
Liberia	0.6 (0.1-2.7)	4.3 (1.5-10.3)	28.1 (21.7-35.4)	69.8 (56.6-80.5)	45 (8.2-86.5)	45.1 (19.9-72.6)	45.4 (36-55.7)	45.9 (30.8-62)
Madagascar	29 (13.7-49.2)	22 (15.9-28.9)	16.9 (10.4-25.8)	14.2 (5.3-30.9)	30.7 (13.8-52.4)	41.4 (32.3-51.4)	53.6 (39.6-66.8)	62.7 (37-83)
Malawi	20.7 (11.4-32.4)	37.7 (29.4-47.2)	59 (48.6-67.8)	73.9 (59.8-84.2)	50.1 (36.8-63.6)	62.5 (55.4-69.7)	73.4 (66-80.1)	80.3 (69.6-87.9)
Maldives	84.6 (42.4-99.2)	88.9 (69.9-97.9)	91.6 (87.1-94.9)	92.4 (86.5-96.2)	93.7 (68-99.7)	85.7 (63.6-96.7)	65.7 (53.8-77)	40.9 (26.4-57.8)
Mali	5 (1.8-10.8)	12.1 (7.9-17.4)	27.7 (18.2-38.7)	46.3 (25.9-68.5)	53.7 (34.9-71.4)	69.7 (62-76.9)	81.9 (74.2-87.9)	88.3 (77.6-94.6)
Morocco	3.4 (1.7-6.4)	17.8 (11.8-25)	57.2 (33.1-78)	83.5 (57.8-96.3)	52.6 (35.9-69.7)	59.4 (48.9-69.1)	65.3 (40.9-83.5)	69 (33.9-91.7)
Mozambique	10.8 (5.2-19.5)	20.8 (14.6-27.6)	37 (29.6-44.8)	52.9 (39.1-67)	76.4 (60.7-86.8)	86.9 (82-90.7)	93.1 (90.8-94.9)	95.8 (93.3-97.6)
Namibia	45.1 (31.9-58.3)	56.6 (48.4-64.6)	67.4 (57.8-75.6)	74.7 (61.7-84.9)	88.1 (81.9-93.1)	82.4 (78-86.4)	74.3 (66.5-81.3)	66.3 (51.7-78.6)
Nepal	0.3 (0.1-0.6)	1.9 (1.2-2.9)	11.5 (8.2-15.5)	37.4 (23.9-52.3)	14.9 (9.6-20.9)	29.8 (24.9-34.7)	51 (45.2-56.2)	68 (59.3-75.1)

Nicaragua	51.2 (5.7-93.4)	32 (22.3-42.5)	23.6 (1-80.8)	22.2 (0.1-94.8)	87 (41.4-99.3)	73 (63.1-80.7)	43.1 (3.9-92.8)	29.6 (0.2-97.5)
Niger	1.9 (0.5-4.8)	4.4 (2.5-7)	10.9 (6.7-17.1)	21.9 (8.8-43.2)	56.8 (30.8-79.8)	61.6 (48.8-73.4)	66 (55.2-76.8)	68.8 (47.4-85.6)
Nigeria	11.5 (2.5-31.7)	7.6 (3.7-13.7)	5.4 (3.8-7.5)	4.5 (1.8-9.5)	26.9 (8.2-55.1)	32.5 (19.9-45.8)	39.7 (31.5-48.1)	46.3 (27.2-64.2)
Pakistan	1.2 (0.6-2.2)	3 (1.9-4.4)	7.2 (5.2-9.7)	14.1 (9.3-20)	24 (17.6-31.5)	22.8 (18.8-26.9)	21.8 (18.3-25.4)	21 (16.4-26.7)
Peru	6.4 (3.6-10.1)	20.6 (16.4-25)	50.1 (43.2-56.9)	74.7 (64.8-83.1)	62 (49-74.1)	67.9 (61.3-74.2)	73.1 (67.1-78.5)	76.7 (67-84.8)
Philippines	3.5 (1.8-6.4)	10 (7-14)	26 (18.8-33.4)	46.8 (32.5-60.3)	26.2 (18.1-35.7)	29.6 (24.5-35.5)	33.4 (28.2-39.3)	36.6 (27.4-46.8)
Rwanda	7.7 (2.9-16.2)	25 (16.6-35.2)	58.4 (46.5-68.6)	81.2 (66.7-90.2)	28.3 (15.4-43.9)	56.7 (46.7-65.8)	81.6 (74.5-87)	92 (85.4-96)
Senegal	15.7 (9.4-24.4)	25.6 (19.9-32.4)	39.4 (35.5-43.3)	52 (45.3-58)	65.6 (51-78.3)	73.6 (66.2-79.9)	80.2 (77.1-82.8)	84.4 (80.9-87.6)
Sierra Leone	0.3 (0-1.3)	2.2 (0.3-7.9)	28.7 (19.9-38.1)	82.2 (62.8-94.5)	0.7 (0-4.3)	4.4 (0.6-16.4)	41.7 (31.4-52.1)	87.8 (70.1-96.3)
South Africa	53.1 (31.5-73.3)	73.5 (63-82.7)	87.3 (81.8-91.5)	93.3 (88.2-96.6)	55.8 (34.7-74.7)	58.1 (45.7-70)	60.2 (50.1-70.7)	61.7 (46.8-76.4)
Tajikistan	8.1 (0-58.2)	18.6 (1.2-64.2)	50.9 (29.4-72)	81.7 (69.1-90.7)	45.1 (1.2-98.4)	68.4 (18.6-97.6)	89.4 (80-95.9)	95.8 (91.7-98)
Tanzania	18.5 (12.3-26.2)	23.7 (19.5-28.1)	30 (25.8-34.5)	35.7 (28.5-44)	76.2 (68.4-83)	76.3 (72.2-80.2)	76.2 (72.7-79.1)	76 (69.8-81.2)
Timor-Leste	0.2 (0-1.4)	0.8 (0.1-3.2)	5.4 (3.1-9)	27.1 (13.9-44.1)	4.6 (0.1-29.8)	16.7 (2.5-46.7)	57.8 (41.3-71.7)	87.9 (77.2-95)
Togo	17 (7-32.3)	26.4 (17.5-36.6)	39.3 (29.5-50.2)	51 (34.1-68.9)	73.8 (51.7-88.9)	73.1 (61.6-82.3)	71.8 (61.5-80.6)	70.4 (54.2-83.3)
Turkey	22.6 (14.8-31.5)	48.8 (42.5-54.8)	75.8 (69.9-81.5)	88.9 (83-93.4)	85.6 (77.7-91.5)	67.7 (61-73.8)	42.1 (32.8-50.8)	24.1 (13.3-36.6)
Uganda	5.7 (2.4-11.3)	15.3 (10.3-21.5)	36.2 (27.6-44.9)	58.5 (41.8-72.7)	34.6 (25.7-44)	45.5 (40.1-50.8)	56.9 (52.1-61.9)	65.6 (58-72.9)
Vietnam	23.8 (5.2-58.7)	39.4 (30.1-51.5)	59 (21.1-89.9)	69.4 (12.6-98.4)	79.1 (45.5-95.4)	93.6 (91-95.7)	97.3 (90.7-99.6)	97.9 (88.8-100)
Yemen	87.4 (0-100)	85 (0.1-100)	70.9 (3-99.9)	2.9 (0-16.9)	20.3 (0-0)	20.3 (0-0.1)	19.7 (0-12.8)	78.5 (0-100)
Zambia	6.3 (2.8-12)	15.3 (10.8-20.4)	33.7 (24.9-42)	53.7 (35.7-68.6)	50.5 (32.4-67.6)	70.6 (63.2-77.6)	85 (80-89.1)	91.6 (85.6-95.9)
Zimbabwe	35.1 (20.6-51.5)	40.2 (31.5-49.4)	45.8 (35.5-55.2)	50.3 (34.1-65.7)	84 (80.1-87.3)	79.2 (76.6-81.4)	73.4 (70.2-76.1)	68 (62.9-72.5)

Table S17: Trends in the utilization (%, 95% credible intervals) of delivery services in private facilities in low- and middle-income countries by wealth quintiles

	Poorest				Richest			
**Country**	**1990**	**2000**	**2010**	**2018**	**1990**	**2000**	**2010**	**2018**
Afghanistan	0.7 (0-5.3)	0.5 (0-2.6)	0.8 (0.4-1.4)	1.7 (0.6-3.8)	30.5 (1-92.8)	22.9 (3.5-66.8)	16 (9.3-26)	13.9 (5.4-29.2)
Albania	N/A	N/A	N/A	N/A	0 (0-0)	0 (0-0.1)	1 (0.6-1.6)	15.5 (9.2-24.9)
Angola	0 (0-0.2)	0.1 (0-0.2)	0.2 (0.1-0.3)	0.4 (0.2-0.8)	6.3 (0.6-29.9)	5.9 (1.7-16.3)	6.5 (4.2-9.4)	7.9 (3.5-14.6)
Armenia	0 (0-0.2)	0.2 (0-0.7)	2 (1.2-3.4)	12.9 (4.3-28)	0.4 (0-1.8)	1 (0.2-2.6)	3.3 (2-5.1)	10.2 (4-19.7)
Bangladesh	0 (0-0)	0.2 (0.1-0.3)	2 (1.2-3.1)	12.7 (5.1-22.9)	4.5 (2.9-6.5)	13.6 (11.1-16.2)	34.6 (29.2-40.5)	58.3 (47.5-68.3)
Benin	4.3 (1.8-8.3)	4.4 (2.8-6.5)	4.7 (3.2-6.6)	5.1 (2.7-8.8)	27.4 (20.1-35.8)	29.4 (25.1-34.2)	31.5 (27.5-35.7)	33.4 (27.3-40)
Bolivia	0.7 (0.2-1.7)	1 (0.6-1.5)	1.5 (0.6-3.1)	2.2 (0.4-7)	46.2 (27.4-64.5)	39.5 (31.2-48)	33.6 (20.1-50.5)	29.8 (11.4-56.2)
Burkina Faso	N/A	N/A	N/A	N/A	3.3 (1.8-5.4)	4.4 (3.3-5.6)	5.9 (3.7-8.8)	7.7 (3.6-14.7)
Burundi	42.8 (2.3-90.6)	14.6 (1.9-39.7)	2.9 (1.5-4.9)	0.9 (0.4-1.6)	20.8 (1-72.3)	13.9 (2.4-40.3)	9.2 (5.1-14.9)	7.5 (3.7-13.2)
Cambodia	0 (0-0)	0.1 (0-0.2)	1.3 (0.7-2.1)	9.7 (2.9-22.1)	4.6 (1.9-9.4)	12.1 (8.2-17)	29.4 (23.8-36.1)	50.3 (35.6-64.5)
Cameroon	5 (2.4-9.2)	4.7 (3.2-6.7)	4.7 (2.4-8.3)	5 (1.6-11.7)	20.9 (13.5-29.1)	28.1 (22.6-34)	36.9 (28.2-48)	44.6 (29.8-61.9)
Chad	0.4 (0.2-1)	0.4 (0.3-0.7)	0.4 (0.3-0.7)	0.5 (0.2-0.9)	1.7 (0.6-4)	2.2 (1.3-3.4)	3 (1.8-4.7)	4.1 (1.7-8.6)
Colombia	3 (1.2-6.5)	2.2 (1.3-3.6)	1.8 (0.8-3.2)	1.5 (0.5-3.7)	39 (18-62.8)	26.6 (15.7-39.2)	17.5 (9.1-30.5)	12.8 (4.1-30.5)
Republic of the Congo	1.5 (0.1-8.4)	2.9 (0.9-7.8)	7.8 (4.8-11.5)	18.7 (5.6-42.3)	10.9 (1-42.4)	9.4 (3.1-21.8)	9.6 (6.1-13.8)	11.4 (3.3-26.8)
Democratic Republic of the Congo	10.8 (0.8-50.7)	7.2 (1.8-20.1)	5.4 (3.4-7.9)	5.2 (1.8-11)	63.6 (13.6-96.4)	59.5 (27.2-86.3)	53 (41.6-63.5)	47.3 (24.4-72.6)
Cote d'Ivoire	0.1 (0-0.3)	0.2 (0.1-0.4)	0.6 (0.3-1.4)	1.8 (0.4-5.7)	1.8 (0.9-3.6)	4.3 (3-6)	10.2 (6.1-16.6)	19.8 (8.5-35.9)
Dominican Republic	3.1 (1.2-6)	3.4 (2.2-4.8)	4 (2.4-6.3)	4.7 (1.9-10.5)	50.8 (35.5-65.3)	57.5 (49.9-64.5)	63.9 (54.8-72)	68.4 (53.8-80.8)
Egypt	2.6 (1.6-4)	10.8 (8.7-13.1)	35.7 (29.9-41.6)	65.1 (54.2-74.8)	38.6 (30.1-48.2)	55.4 (51.2-59.8)	71.2 (66.5-75.9)	81 (74.6-86.6)
Ethiopia	0 (0-0)	0 (0-0)	0 (0-0)	0.1 (0-0.1)	0.2 (0.1-0.5)	0.8 (0.4-1.3)	3.2 (2.3-4.3)	9.6 (5.3-16)
Gabon	4.7 (1.4-11.3)	4.5 (2.5-7.3)	4.6 (2.8-7.2)	5 (1.9-11)	26.2 (8.8-52)	35.4 (22.1-49.4)	47.1 (34.6-59.5)	56.6 (35.2-76.5)
Ghana	7.1 (3.9-11.5)	3.7 (2.6-5)	1.9 (1.3-2.7)	1.1 (0.6-2.1)	23.3 (15.2-32.6)	21.6 (17.5-26)	20.1 (15.3-25.5)	19.2 (11.9-28.2)
Guatemala	0.3 (0.1-0.6)	0.2 (0.1-0.4)	0.2 (0.1-0.4)	0.2 (0.1-0.5)	30.6 (20.9-43.1)	32.3 (26.5-40.1)	34.3 (26.2-42.8)	36 (23.4-50.9)
Guinea	N/A	N/A	N/A	N/A	2 (0.8-4.5)	5.8 (3.8-8.5)	16.6 (11.6-23.1)	34.2 (19-52.7)
Haiti	1 (0.3-2.4)	1.1 (0.6-1.8)	1.3 (0.8-2)	1.6 (0.6-3.2)	37.1 (22.6-53.6)	31 (22.8-39.4)	25.6 (19.6-32.3)	22 (14.2-31.9)
Honduras	0.2 (0-1.2)	0.4 (0.1-1.1)	1.1 (0.7-1.7)	3.6 (0.9-9.7)	40 (4.7-90.5)	27.6 (9.7-56.8)	17.5 (11.9-24.7)	13.6 (3.5-34.4)
India	1.3 (0.7-2.2)	2.8 (2-3.8)	6.1 (4.1-8.7)	11.4 (6.3-18.5)	41.8 (30.2-53.8)	50.1 (42.9-57)	58.5 (49.3-67.3)	64.7 (51.6-76.3)
Indonesia	0.5 (0.1-1.3)	2.3 (1.3-4)	11.2 (7.3-16.1)	32.7 (18.1-48.6)	35 (18-55)	53.3 (41.8-64.1)	71.2 (63.7-77.8)	81.9 (71.9-89.5)
Jordan	6.6 (4.4-9.6)	10.9 (8.6-13.6)	17.8 (14.3-21.9)	25.5 (18.3-34.1)	59.2 (45.4-72)	65.5 (57.8-73)	71.2 (63.3-78.1)	75.1 (63.8-83.9)
Kenya	5.7 (2.9-9.8)	4.4 (3-6.3)	3.5 (2.1-5.4)	3 (1.3-5.8)	22.6 (15.6-31.2)	28.2 (23.3-33.5)	34.7 (28.2-41.6)	40.4 (28.9-51.7)
Lesotho	0.3 (0.1-1)	0.6 (0.2-1.1)	1.2 (0.9-1.6)	2.4 (1.1-4.6)	0.2 (0-1)	1 (0.2-2.4)	5.7 (3.5-8.7)	24 (8.1-48.3)
Liberia	7.5 (0.9-31)	4.7 (1.5-12)	3.2 (2.3-4.3)	2.6 (1.4-4.6)	20.5 (3.5-57)	24.3 (10.2-45.1)	30.1 (23.4-37.2)	36.1 (24-48.5)
Madagascar	2.3 (0.7-5.7)	0.5 (0.3-0.8)	0.1 (0.1-0.2)	0 (0-0.1)	3.8 (1.4-8.1)	7.4 (4.9-10.3)	14.9 (8.9-22.6)	25.3 (10.7-47)
Malawi	11.1 (6.1-18.4)	12.1 (8.6-16.3)	13.5 (9.4-18.2)	14.9 (8.3-23.1)	23.6 (13.2-36.3)	21.5 (15.6-28.4)	19.9 (14.4-27.5)	19 (10.7-30.4)
Maldives	0.8 (0-3.9)	1.4 (0.3-4.4)	3.5 (2.1-5.5)	8.4 (4.2-14.7)	6.9 (0.3-31.2)	14.7 (2.9-37.7)	33.5 (21.4-45.8)	56.9 (40.5-71.6)
Mali	0.1 (0-0.3)	0.4 (0.2-0.6)	1.3 (0.6-2.3)	4 (1.1-9.9)	1.4 (0.8-2.1)	3.2 (2.5-4)	7.3 (5.8-9.3)	13.7 (9-20.1)
Morocco	N/A	N/A	N/A	N/A	18.5 (9.8-29.5)	27.7 (19.9-37.7)	39.9 (21-64.7)	50.2 (19.8-82.7)
Mozambique	0.1 (0-0.2)	0.1 (0.1-0.2)	0.2 (0.1-0.3)	0.3 (0.2-0.7)	0.1 (0-0.2)	0.2 (0.1-0.4)	0.7 (0.4-1.1)	2 (0.8-4)
Namibia	4.2 (0.5-15.7)	1.2 (0.5-2.6)	0.4 (0.2-0.8)	0.2 (0.1-0.7)	6.1 (3.7-9.3)	12.3 (9.5-15.7)	23.6 (17.7-30.3)	36.6 (25.2-49.4)
Nepal	0.4 (0.1-0.7)	0.6 (0.4-0.9)	1 (0.7-1.4)	1.6 (0.8-2.6)	2.7 (1.2-5.5)	6.9 (4.5-10.2)	17.2 (12.7-23.1)	32.4 (20.1-46.7)
Nicaragua	3.2 (0.1-18.6)	0.3 (0.2-0.5)	0.2 (0-1.8)	0.7 (0-4.8)	3.8 (0.2-20)	22.3 (15.4-30)	74.8 (16-98.3)	89 (14.1-100)
Niger	0.2 (0-1)	0.1 (0-0.4)	0.2 (0.1-0.2)	0.2 (0.1-0.6)	0.5 (0.2-1.2)	1 (0.6-1.6)	2.2 (1.4-3.4)	4.4 (1.7-9.2)
Nigeria	25.5 (7.2-56.6)	7.6 (3.8-13.3)	1.9 (1.3-2.6)	0.7 (0.3-1.5)	58.3 (26.2-84.1)	50.6 (35.5-65.8)	42.4 (34.8-50.3)	36.4 (20.8-55.4)

Pakistan	0.6 (0.3-1.1)	2.9 (2-4.1)	13 (9.7-17.1)	35.3 (25.2-45.8)	23.1 (16.2-32.2)	39.4 (33-46.5)	58.6 (52.2-64.1)	72.4 (64.3-78.3)
Peru	0.8 (0.5-1.3)	0.8 (0.6-1.1)	0.8 (0.6-1.1)	0.9 (0.5-1.3)	30.1 (19.3-41.8)	27.8 (22-33.3)	25.9 (20.9-31.9)	24.6 (16.2-35)
Philippines	0.5 (0.3-0.9)	1.3 (0.9-1.8)	3.2 (2.3-4.4)	6.7 (3.9-10.8)	42.6 (29.7-55.6)	49.4 (41.4-57)	56.2 (49.4-63.4)	61.5 (49.8-71.4)
Rwanda	0.7 (0.1-2.1)	0.5 (0.3-1)	0.6 (0.3-1)	0.7 (0.1-2)	6.3 (2.5-13.1)	5 (3.2-7.4)	4.1 (3.1-5.1)	3.6 (2-5.9)
Senegal	0.1 (0-0.3)	0.2 (0.1-0.3)	0.3 (0.2-0.4)	0.5 (0.3-0.7)	15.7 (9.7-22.7)	15 (11.4-18.8)	14.5 (12.6-16.3)	14.1 (11.6-16.7)
Sierra Leone	47.3 (3.5-96.9)	8.1 (1.2-28.7)	0.5 (0.3-0.7)	0.1 (0-0.2)	37.1 (1.5-94.8)	22.9 (4.1-62.6)	11.6 (7.8-16.8)	8.3 (1.8-22.7)
South Africa	0.9 (0.3-1.9)	0.5 (0.3-0.8)	0.3 (0.2-0.4)	0.2 (0.1-0.3)	43.4 (22.8-64)	40.9 (28.3-53.7)	38.7 (29.5-48.6)	37.1 (23.5-53.1)
Tanzania	2.9 (1.5-5.2)	4.1 (2.8-5.5)	5.9 (4.3-7.7)	8.1 (4.8-12.5)	3.7 (2.1-5.9)	7.3 (5.5-9.3)	14 (11.4-16.8)	22.6 (16.6-29.2)
Togo	0.4 (0.2-1)	0.4 (0.3-0.7)	0.5 (0.3-0.7)	0.5 (0.2-1.1)	14.5 (5.7-28.2)	18.5 (11.3-27.7)	24.2 (16.4-33)	29.8 (16.5-45.7)
Turkey	1.3 (0.6-2.3)	3.1 (2.2-4.2)	7.8 (5.3-11.5)	15.7 (8.2-26.9)	10.8 (6.5-17.9)	28.7 (22.9-34.5)	57.6 (47.9-66.6)	77.9 (64.4-87)
Uganda	6.3 (3.4-10.9)	6.5 (4.7-8.7)	6.8 (4.9-9)	7.2 (4.1-11.2)	27 (17.6-37.8)	28.3 (22.7-33.9)	29.8 (24.8-35.2)	31.1 (22.5-40.5)
Vietnam	0.8 (0.1-2.6)	3.4 (2.2-5.1)	20.4 (3.4-60.7)	47.8 (4.3-96.5)	0.6 (0.1-2.3)	2.8 (1.7-4)	17.8 (2.6-51.8)	45.5 (2.6-95.1)
Zambia	6.4 (3.8-10.2)	5.8 (4.4-7.4)	5.3 (4.1-6.8)	5 (3.1-7.8)	40.3 (22.2-62)	20.5 (14.2-27.8)	9.1 (5.7-13)	4.7 (1.9-9.2)
Zimbabwe	19.9 (12.1-30)	13.3 (10.1-17.1)	8.8 (6.5-11.9)	6.3 (3.9-10)	9.3 (5.1-15.2)	14 (10.7-18.1)	21 (16.3-26.1)	28.4 (18.7-39)

Note: N/A denotes not application and refers that the indicator has no sufficient data to examine trends

Table S18: Trends in the utilization (%, 95% credible intervals) of delivery services in public facilities in low- and middle-income countries by place of residence

	Rural				Urban			
**Country**	**1990**	**2000**	**2010**	**2018**	**1990**	**2000**	**2010**	**2018**
Afghanistan	6.2 (0.1-49.5)	10.2 (1-40.4)	23.8 (14.7-36)	47.3 (24.9-70.3)	26.9 (0.4-88.4)	36.4 (5.3-79.9)	53.6 (40.5-67.7)	68.1 (46-86.7)
Albania	93.9 (72.9-99.6)	96 (89.1-99.1)	97.1 (95.5-98.4)	97.5 (95.6-98.6)	100 (99.8-100)	99.8 (99.4-100)	98.7 (97.8-99.3)	93.3 (88.5-96.2)
Angola	35.1 (5.2-79.8)	27.6 (9.4-54.5)	21.1 (14.7-30)	17.7 (8.9-31.8)	90.1 (61.1-98.9)	84.5 (66.5-94.7)	73.4 (63.6-80.9)	59.8 (40.7-76.4)
Armenia	82.9 (60.8-95)	90.2 (83-94.9)	94.3 (91.5-96.2)	96.1 (92.5-98.3)	98.7 (96.1-99.7)	98.1 (96.6-99)	96.9 (95.4-98.1)	95.2 (90.3-97.9)
Bangladesh	0.8 (0.5-1.2)	2.7 (2.2-3.4)	9 (7.1-11.4)	21.4 (15.1-29.7)	11.3 (7.8-15.3)	13.4 (11.1-15.6)	15.9 (12.9-18.9)	18.3 (13.2-23.8)
Benin	51.4 (36.1-66.2)	61.7 (53.3-70)	71 (64.2-76.7)	77.3 (68-84.9)	58.2 (45-68.9)	63.3 (56.4-69.7)	68 (63.1-72.8)	71.5 (64.1-78.5)
Bolivia	11.6 (6.9-17.8)	25.8 (21.1-30.6)	48.7 (36.6-60.8)	67.3 (48.4-82.9)	37.2 (25.6-50.7)	57.8 (51.9-63.4)	76 (66-84)	85.7 (73.2-93.6)
Brazil	62 (44.9-76.2)	81.6 (56.6-93.9)	89 (40.8-99.5)	91.1 (28.5-99.9)	80.2 (68.1-90.3)	79.6 (57.3-92.4)	75.8 (21.4-98.2)	72.9 (7-99.5)
Burkina Faso	23.3 (11.5-39.1)	36.2 (27.7-45.2)	52.1 (35.5-67.7)	64.1 (37.3-84.4)	85 (75.5-91.7)	85.9 (81.8-89.5)	86.3 (79.5-91.4)	86.3 (74.2-94.1)
Burundi	2.8 (0.1-17.2)	13.4 (2.2-38.9)	55.4 (39.7-69)	88.3 (79-94)	41.8 (3.7-90.4)	59.4 (22.3-88.8)	77.9 (67.6-86.5)	87.3 (78.3-93.4)
Cambodia	0.4 (0.1-0.9)	5.3 (3.3-8)	44.5 (36.8-52.5)	86.9 (78.1-92.9)	9.4 (3.7-18.9)	25.7 (18.1-35.4)	54.9 (46.7-62.1)	76.7 (63.4-85.7)
Cameroon	36.4 (26.1-48.3)	33.8 (28.4-39.6)	31.4 (23.7-40.1)	29.7 (18.4-44)	64.7 (54.4-74.1)	58.2 (52-64.8)	51.2 (41.1-60.5)	45.7 (31.2-60.2)
Chad	1.6 (0.7-3.2)	3.8 (2.5-5.6)	9.2 (6.4-13)	18.2 (9.6-28.6)	26.9 (15.5-41.8)	36.6 (28.9-45.4)	48 (38.9-56.5)	57.2 (41.8-71)
Colombia	52.7 (39.1-66.4)	66.4 (58.5-73.2)	77.7 (69.5-84.5)	84.4 (74.2-91.5)	67.8 (46.9-84.4)	78 (68.5-85.4)	85.2 (76.1-91.8)	89 (76.8-96)
Comoros	23.1 (11.2-38.7)	44.2 (32.5-54.4)	68.1 (56.2-77.9)	82.2 (68-91.8)	59.6 (36.4-77.2)	73 (61.8-81.2)	83.1 (74.9-89)	88.4 (77.4-94.8)
Republic of the Congo	53 (10.1-91)	63 (37.7-83.4)	72.7 (63-80.8)	77.6 (54.3-92.7)	86.8 (49.6-98.8)	85.1 (69.1-94.7)	80.5 (73-86.6)	73.7 (48.6-90.9)
Democratic Republic of the Congo	22.6 (1.3-73.4)	37.6 (11.8-70.6)	60.5 (50-69.9)	76.2 (52.3-90.3)	38.2 (4.6-87.8)	43.6 (17-73.7)	50.9 (39.3-62)	56.9 (33.2-79.8)
Cote d'Ivoire	26.9 (16.6-39.2)	34.7 (27.9-42.2)	43.8 (34.1-54.5)	51.4 (34.2-68.9)	75.5 (62.6-84.7)	76.2 (71-81.2)	76.6 (69-82.9)	76.7 (62.9-86.2)
Dominican Republic	77.2 (67.1-84.9)	78.3 (73.6-82.5)	79 (70.9-85.4)	79.3 (66.5-89.1)	66 (54.8-75.8)	70.3 (64.1-75.6)	74 (66.6-81.7)	76.6 (64.3-86.6)
Egypt	9.9 (7-13.4)	15.3 (12.7-17.8)	23 (18.3-27.8)	30.9 (21.9-41)	32.7 (28.2-37.6)	32.6 (30.2-35.2)	32.5 (29.3-35.8)	32.5 (27.5-38.2)
Ethiopia	0.3 (0.1-0.7)	1.3 (0.6-2.3)	6.7 (4.4-9.6)	23 (11.4-37.8)	11.9 (3.8-25.9)	28.5 (17.5-40.4)	55.8 (47.1-63.8)	75.9 (61.9-85.6)
Gabon	58.7 (31.8-82.6)	62.1 (47.1-75)	65.2 (54.1-74.8)	67 (46-83.6)	77.1 (49.3-92.2)	73.7 (59.6-84.6)	68.8 (57.3-78.8)	64.1 (42.4-82.4)
Ghana	15.2 (8.9-23.8)	27 (21.4-33.5)	43.9 (33.9-53.4)	58.5 (42.4-73.5)	51.9 (38.3-63.9)	62.2 (55.7-67.6)	71.4 (63.4-77.6)	77.6 (65.9-86)
Guatemala	12.4 (7.4-19.4)	24.4 (18.9-31)	43 (32.9-52.9)	59.5 (42.7-73.7)	47.5 (34-60)	56.7 (48.3-63.4)	65.5 (56.1-72.7)	71.7 (58.1-81.5)
Guinea	13.4 (5.5-27.7)	18.6 (12.8-26)	26.3 (18.7-33.4)	34 (18.6-51.4)	60.3 (37.7-77.6)	58.7 (48.1-68.3)	56.7 (47.5-66)	55 (37.7-72.2)
Haiti	3 (1.5-5.1)	6.7 (4.8-9.2)	14.8 (11.4-18.7)	26.2 (17.9-35.9)	18.4 (11.2-27.8)	27.7 (22.4-34)	39.8 (33.8-45.8)	50.5 (39.6-60.3)
Honduras	12.8 (0.5-61.1)	30.9 (9.5-63.3)	66 (55.8-75.7)	85.5 (63.7-96.6)	46.8 (6.3-93.2)	68.9 (40-89.7)	86.4 (80.2-91)	92.5 (79.2-98)
India	6.3 (2.9-11.9)	15.3 (10.7-21.2)	33.3 (22.7-44.2)	52.9 (33.7-69.2)	25.7 (16.2-37.2)	31.6 (25.3-38.7)	38.6 (30.3-47.2)	44.5 (31.1-58.9)
Indonesia	3.2 (2-4.7)	7.3 (5.6-9.3)	16.1 (12.1-21.3)	28.1 (19.2-40.1)	16 (11.1-22.3)	17.7 (14.3-21.3)	19.7 (14.9-24.9)	21.5 (13.9-30.9)
Jordan	72.9 (65.7-78.8)	78.3 (75.1-81.4)	82.9 (79.8-85.4)	86 (81.9-89.1)	53.6 (48.6-58.4)	57 (54-59.8)	60.3 (57.1-63.5)	62.9 (57.9-67.5)
Kazakhstan	97.2 (90.9-99.5)	97.1 (94.4-98.7)	93.7 (67-99.8)	88.4 (24.5-99.9)	96 (85.3-98.3)	100 (100-100)	100 (100-100)	100 (100-100)
Kenya	23.4 (13-35.9)	27.9 (21.2-35.1)	33.4 (24.9-43)	38.3 (23.8-55.6)	51.7 (38.5-64.9)	51.6 (44.5-58.5)	51.5 (43.1-59.5)	51.4 (37.4-64.2)
Kyrgyz Republic	89.5 (78.8-95.7)	96.6 (94.5-97.9)	98.9 (98.2-99.3)	99.5 (99-99.8)	97.9 (94.9-99.2)	98.8 (98-99.3)	99.3 (98.8-99.6)	99.5 (98.8-99.8)
Lesotho	16.5 (2.6-47.6)	32.7 (15.9-52.9)	58.5 (48.6-66.9)	76.4 (58.3-89.2)	83.4 (53.3-96.4)	83.8 (70.9-92.1)	83.1 (78.1-87.4)	81.4 (67.6-91.4)
Liberia	1.2 (0.1-5.4)	6.4 (1.9-16.5)	33.1 (24.9-42.5)	72.3 (58.7-83.2)	35.8 (8.5-75.7)	42.1 (21.5-66)	49.8 (41.3-58)	56.2 (43.2-69.1)
Madagascar	40.5 (27.8-53.6)	32.9 (26.7-39.9)	26.4 (17.1-37.4)	22.2 (10.5-39.6)	42.2 (28-57.4)	43 (35.4-51.3)	44 (29.3-58.2)	44.9 (23-67.1)
Malawi	24.1 (13.6-37.8)	41 (31.8-51.5)	60.9 (50.2-71)	74.5 (60.6-85.3)	61.4 (45.4-75.2)	70.5 (62.7-77.4)	78.1 (71.8-83.6)	82.8 (73.4-89.4)
Maldives	92.9 (66.2-99.6)	92.6 (78.3-98.3)	91 (86.1-94.5)	88.2 (80.7-93.6)	89.7 (58.4-99.5)	84 (59.9-96.6)	72.4 (61.1-81.5)	57.7 (40.6-72.5)
Mali	7.6 (3.1-16.2)	18.2 (12.1-25.7)	39 (27.1-52.2)	59.3 (35.6-78.4)	53.1 (32-72.2)	67.6 (57.9-75.9)	79.3 (70.4-86.4)	85.6 (73.2-93.9)
Morocco	11.6 (5.7-21.3)	30.1 (21.1-39.1)	58.8 (36.6-78.1)	77.4 (47.6-94.2)	46.8 (30.9-65)	64.3 (53-74)	78 (58.8-90.1)	85.1 (59.6-96.5)
Mozambique	22.6 (10.5-38.1)	34.7 (25.6-44.1)	49.9 (41.2-58.2)	62.1 (47.1-74.2)	77.2 (62.3-88.6)	82.2 (75.5-87.4)	86 (81.5-89.8)	88.3 (81.2-93.3)
Namibia	53.1 (41.1-66.1)	65.4 (59.2-72)	75.9 (68.6-81.7)	82.4 (72.7-89.4)	84.5 (75.8-90.8)	85.8 (81.1-89.5)	86.8 (81.5-91.1)	87.3 (78.6-93.5)

Nepal	1.5 (0.7-2.8)	6 (4.2-8.3)	21.3 (16.4-27.2)	46.2 (33.2-59.9)	28.2 (17.5-41.4)	39 (31.7-46.7)	51.4 (43.3-57.9)	61.2 (48.5-71.5)
Nicaragua	40.1 (3.7-89.1)	45.7 (35.2-57.7)	53.1 (5.4-96)	56.2 (0.9-99.7)	80.4 (31-99.1)	78.9 (71.4-85.4)	66.2 (9.3-97.4)	57.9 (0.7-99.6)
Niger	4.2 (2.4-7.2)	8.7 (6.2-11.8)	17.7 (11.3-25.7)	29.4 (15-46)	67.8 (54.5-80.5)	72.7 (65.5-79.3)	76.8 (67.5-84.5)	79.4 (65.4-89.4)
Nigeria	21.2 (13.9-30.4)	18.4 (14.5-22.6)	16 (11.7-21)	14.4 (8.9-22.3)	53.2 (36.8-68.4)	42.4 (32.4-51.5)	32.4 (23-42.6)	25.6 (13.9-40.6)
Pakistan	3.2 (1.7-5.1)	6.3 (4.3-8.9)	12.2 (9-15.8)	20.1 (13.7-27)	16.1 (9.7-24.6)	18.6 (13.8-24)	21.5 (16.8-26.5)	24.2 (17.4-33)
Peru	8.1 (5.8-11.2)	26.7 (23.1-30.4)	60.2 (55.9-64.6)	82.4 (77.1-86.8)	55.5 (46.9-64.5)	71.7 (68-75.2)	83.7 (81.2-86)	90 (86.7-92.5)
Philippines	6.6 (3.9-10.5)	16.5 (12.7-21.4)	36 (28.8-42.7)	56.4 (44.3-67.3)	21.4 (14.3-28.9)	30.8 (25.4-36.2)	42.3 (36.8-48.5)	52.2 (42.7-61.8)
Rwanda	9 (3.4-18)	30 (20.8-40.2)	66.1 (54.6-75.7)	86.4 (74-93.7)	45.7 (25.7-65.9)	64.2 (53.3-74.3)	79.4 (71.1-85.9)	87.3 (76.8-94)
Senegal	23.7 (20.9-26.7)	39.1 (37-41.3)	57.1 (55.6-58.6)	70.4 (68.5-72.3)	67.5 (60.9-74.1)	75.8 (72.5-79.2)	82.4 (80.8-84.1)	86.6 (84.5-88.6)
Sierra Leone	0.5 (0-4)	2.8 (0.3-12.3)	31.1 (22.1-41.3)	82.8 (54.4-95.4)	1.2 (0-7.2)	6.2 (0.9-22.1)	44 (33.7-54.6)	86.2 (66.6-96.1)
South Africa	62.7 (40.3-80.8)	77.1 (66.3-85.5)	87 (81.7-91.5)	91.9 (86.2-96)	76.7 (57.5-89.9)	81.3 (72-88.7)	84.9 (78.6-89.8)	87.1 (77.8-93.4)
Tajikistan	15.2 (0.1-82.4)	32.3 (3-81.8)	67.5 (46.2-83.4)	88.7 (79.2-94.4)	30.7 (0.6-94.3)	56.5 (11.3-93.7)	85.8 (72.8-93.6)	95.4 (91.5-97.9)
Tanzania	31 (22.4-42)	34.6 (29.3-40.6)	38.6 (32.4-45.9)	41.9 (30.9-53.2)	75.8 (69.4-81.4)	74.3 (70.5-78.1)	72.6 (68.3-76.7)	71.1 (64.2-77.5)
Timor-Leste	0.5 (0-2.9)	2 (0.2-7.4)	12.6 (6.9-20.1)	46.8 (28.8-66.5)	2.8 (0-20.2)	11.1 (1.2-40.6)	50.8 (35.9-67.3)	87.2 (75-94.1)
Togo	28.9 (12.9-49.4)	40.4 (28.2-51.7)	53.8 (42.2-64.5)	64 (47-79)	70.6 (49.4-85.7)	70.2 (58.3-79.9)	69.3 (58.1-78.4)	68.3 (50.4-82.5)
Turkey	41.7 (30.1-55.2)	59.2 (52.6-65.3)	74.6 (67.9-80.3)	83.6 (74.8-89.8)	72.7 (61.2-82.4)	68.2 (62-74)	63.2 (54.2-71.3)	58.8 (42.2-72.6)
Uganda	9.6 (5.1-15.4)	20.9 (15.7-26.3)	40.4 (33.2-47.1)	58.9 (46.7-69.3)	42.7 (30.7-55.5)	51.7 (45.4-58.5)	60.6 (55-65.2)	67.3 (58.6-74.6)
Vietnam	34.1 (8.3-70.2)	64.5 (54.6-73.8)	84.7 (50.4-97.9)	90.5 (43.5-99.8)	76.5 (42-94.8)	93.1 (89.7-95.5)	97.3 (89.9-99.6)	98.1 (88.5-100)
Yemen	7.1 (3.6-12.1)	10.1 (6.7-14.7)	14.7 (9.4-21.7)	19.7 (10.4-32.9)	33.6 (20.5-47.7)	32.6 (23.3-42.4)	32 (21.6-44.1)	31.6 (17.6-48.4)
Zambia	12.7 (7.5-19.6)	23.4 (18-29.5)	39.6 (29.7-50.3)	54.5 (38.1-70.5)	55.4 (44.6-66.1)	70.1 (64.9-75)	81.5 (75.9-86.1)	87.8 (81.2-92.7)
Zimbabwe	43.8 (28.8-60.7)	48.7 (40.2-57.7)	53.8 (45.3-62.1)	57.8 (43.3-72.5)	83.3 (74.6-89.6)	81.3 (76.4-85.3)	78.8 (73.7-83.3)	76.5 (67.6-84.1)

Table S19: Trends in the utilization (%, 95% credible intervals) of delivery services in private facilities in low- and middle-income countries by place of residence

	Rural				Urban			
**Country**	**1990**	**2000**	**2010**	**2018**	**1990**	**2000**	**2010**	**2018**
Afghanistan	12.6 (0.1-71.4)	6.5 (0.5-27.6)	3.4 (1.8-5.9)	2.9 (0.9-6.3)	28.8 (0.4-94)	21.2 (1.9-67.5)	14.5 (8.3-23.4)	13.1 (4.5-30.6)
Albania	N/A	N/A	N/A	N/A	0 (0-0)	0.1 (0-0.2)	0.8 (0.5-1.4)	6.4 (3.6-10.9)
Angola	0.1 (0-0.3)	0.1 (0-0.3)	0.3 (0.2-0.4)	0.6 (0.3-1.1)	3.3 (0.2-15.4)	2.6 (0.7-6.9)	2.5 (1.6-3.7)	2.8 (1.2-5.2)
Armenia	0 (0-0)	0.1 (0-0.2)	1.5 (0.8-2.4)	17 (6.2-35.4)	0 (0-0.2)	0.3 (0.1-0.6)	2.2 (1.3-3.5)	11.5 (4.3-23.9)
Bangladesh	0.2 (0.1-0.4)	1.5 (1.2-1.9)	9 (7-11.1)	30.8 (22.2-40.8)	6.3 (3.4-10.4)	12.2 (9.2-15.8)	23 (17.2-29.5)	35.6 (23.1-49.3)
Benin	5.6 (2.9-9.7)	6.7 (4.6-9)	8.1 (6.2-10.5)	9.6 (6-14.2)	20.2 (14.9-26.5)	20.9 (17.8-24.5)	21.7 (19.3-24.3)	22.4 (18.3-27.2)
Bolivia	8.4 (3.7-15.8)	4.2 (2.9-6)	2.3 (1-4.3)	1.5 (0.4-4.1)	20.2 (10.2-33.8)	16.9 (12.4-22)	14.9 (7.7-25.1)	14 (4.5-32)
Brazil	2.7 (1.3-5.2)	9.4 (2.5-22.5)	29.5 (1.5-82.1)	48.6 (0.9-97.8)	8.2 (3.8-14.9)	26.5 (9.5-53.2)	55.2 (8.2-96.2)	71.1 (6.5-99.7)
Burkina Faso	0.1 (0-0.1)	0.1 (0-0.1)	0.1 (0-0.2)	0.1 (0-0.3)	5.2 (2.8-9)	5.4 (4-7.2)	5.9 (3.5-9.3)	6.6 (2.7-13.2)
Burundi	26 (1.5-78.3)	11.9 (2.1-34.4)	4.5 (2.5-7.7)	2.3 (1.1-4.5)	13.2 (0.5-58.7)	10.9 (1.7-33.1)	9.8 (5.5-15.4)	10.4 (5.1-17.1)
Cambodia	0.2 (0.1-0.4)	1.1 (0.7-1.7)	6.9 (5.2-8.8)	25.4 (16.1-37.3)	2.5 (1-5.4)	8.2 (5.2-11.9)	24.7 (19.6-30.2)	48.5 (35.5-62)
Cameroon	10.1 (7.2-13.4)	11.5 (9.6-13.8)	13.3 (10-17.7)	15 (9.6-22.7)	17.2 (11.1-24.4)	24.9 (20-30.5)	34.9 (25.6-44.9)	44 (28.5-60.9)
Chad	1 (0.3-2.6)	0.6 (0.3-1.1)	0.4 (0.2-0.7)	0.3 (0.1-0.8)	1.4 (0.6-3.1)	2 (1.3-2.9)	2.9 (1.8-4.3)	4 (1.8-7.8)
Colombia	5.5 (2-12.6)	3.3 (1.9-5.6)	2.2 (1-3.8)	1.6 (0.5-3.8)	22.4 (8-42)	12.9 (6.9-20)	7.5 (3.5-13.6)	5.1 (1.4-13.1)
Comoros	N/A	N/A	N/A	N/A	0.2 (0.1-0.3)	0.7 (0.4-1.1)	3.5 (1.9-5.6)	12.2 (5-23.3)
Republic of the Congo	1.9 (0.1-9.7)	3.2 (0.9-8)	7.3 (4.8-10.9)	16 (5-37.2)	7.7 (0.6-31.7)	10.3 (3.3-22.5)	16.9 (11.2-24.1)	26.6 (9.4-52.3)
Democratic Republic of the Congo	25.8 (1.7-73.7)	14.2 (3.7-35)	7.3 (4.8-10.8)	4.9 (1.7-10.8)	46.5 (5.9-90.9)	43.9 (15.8-73.8)	41 (31.4-51.9)	39.6 (19.6-64.3)
Cote d'Ivoire	0.4 (0.1-0.8)	0.7 (0.4-1.2)	1.7 (0.9-2.9)	3.5 (1.2-8.1)	1 (0.6-1.5)	2.6 (1.9-3.3)	6.8 (4.6-9.7)	14.5 (7.6-23.8)
Dominican Republic	12.6 (8.3-18)	15.7 (12.8-18.8)	19.5 (14.5-25.3)	23.2 (14.3-33.7)	32.4 (22.6-43.3)	28.6 (23.3-34)	25.3 (18.2-33.3)	23 (13.2-35.5)
Egypt	5.4 (3.8-7.2)	19.9 (17-22.4)	52.1 (46.3-58.1)	77.8 (71-84.1)	18.7 (14.3-23.8)	35.8 (32.3-39.1)	57.5 (51.6-62.7)	73.3 (65.3-79.5)
Ethiopia	0 (0-0.1)	0.1 (0.1-0.1)	0.2 (0.2-0.3)	0.5 (0.3-0.8)	0.4 (0.1-0.8)	1.2 (0.7-1.8)	4.2 (3.1-5.6)	11.2 (6.3-17.9)
Gabon	6 (1.5-14.8)	5.4 (3-9.2)	5.4 (3.4-8.3)	5.7 (2.1-12.4)	15 (4.7-33.1)	20 (12-30.3)	27.4 (18.3-38.9)	34.9 (16.8-57.4)
Ghana	8.5 (5.4-13.1)	6.4 (5.1-8.1)	4.9 (3.6-6.5)	4 (2.4-6.3)	22 (16-29.3)	18.3 (15.4-21.4)	15.2 (12-18.6)	13.1 (8.7-18.7)
Guatemala	3.4 (1.7-6.1)	3.8 (2.6-5.5)	4.5 (2.6-6.6)	5.2 (2.4-9.7)	11 (6.9-16.8)	12.8 (9.7-16.2)	15 (10.7-20.1)	17.1 (10-26.2)
Guinea	0.1 (0-0.4)	0.3 (0.2-0.5)	0.7 (0.4-1.1)	1.4 (0.4-3.7)	1.1 (0.4-2.6)	3.7 (2.4-5.5)	12.5 (8.7-17.5)	29.5 (15.5-47.4)
Haiti	4.4 (1.8-8.7)	4.5 (2.8-6.9)	4.8 (3-6.9)	5.2 (2.4-9.7)	26.1 (14.7-41)	19.3 (14-25.6)	14.1 (10.6-18.5)	11 (6.7-17.3)
Honduras	2.1 (0.1-11.5)	2.1 (0.5-5.8)	3.2 (2.1-4.8)	5.7 (1.6-14.7)	26.3 (1.5-80.8)	14.3 (3.7-35.9)	7.4 (4.7-10.9)	5.4 (1.2-15.1)
India	6.5 (3.9-10)	10.7 (8-13.5)	17.3 (12.4-22.9)	24.9 (15.3-36.1)	28.5 (20.9-37.4)	33.9 (28.7-39.8)	40 (33.5-46.9)	45 (34.9-55.6)
Indonesia	3.7 (2.3-5.5)	10.2 (8-13)	25.5 (19.7-32)	45.3 (32.5-57.8)	24.7 (17.9-32.8)	40.1 (34.7-45.8)	57.8 (50.8-64)	70.7 (61.2-78.4)
Jordan	8.5 (5.1-12.9)	11.3 (8.5-14.5)	15.1 (11.4-19.4)	19 (12.7-26.6)	35.4 (27.9-43.5)	36.6 (31.8-41.5)	37.8 (32.9-43)	38.8 (31.1-46.4)
Kenya	9.3 (5.6-14.1)	9.3 (7.2-11.7)	9.4 (6.9-12.4)	9.7 (5.6-14.9)	18 (12.8-25.3)	21.9 (18.1-26)	26.5 (21-32.2)	30.7 (21.7-40.8)
Lesotho	0.9 (0.2-3.1)	1.4 (0.6-2.9)	2.5 (1.8-3.3)	4.2 (1.9-7.6)	1 (0-5.3)	1.7 (0.4-4.6)	4.5 (2.7-7)	11.4 (3.3-28.5)
Liberia	7.3 (1-24.8)	6.3 (2.4-13.7)	6 (4.4-8.1)	6.2 (3.7-10.2)	21.5 (3-61.7)	20.3 (6.9-41.4)	20.3 (15-27.5)	21.4 (12.2-33.4)
Madagascar	0.6 (0.3-1.1)	1.2 (0.8-1.7)	2.4 (1.2-4.2)	4.3 (1.3-9.7)	3.5 (1.6-6.4)	5.6 (3.9-7.7)	9.7 (5.2-17.2)	15 (5.5-33.1)
Malawi	17.3 (9.5-28.4)	17 (12.2-23)	17.1 (11.9-23.9)	17.5 (9.7-29.2)	18.6 (10.8-28.4)	15.5 (11.6-20.4)	13.1 (9.2-17.5)	11.6 (6.5-17.9)
Maldives	2.1 (0.1-11.4)	3.2 (0.7-10.2)	6.4 (3.9-10.3)	12.5 (6.5-20.8)	10.1 (0.5-43.2)	15.8 (3.2-38.5)	27.3 (17.6-38.4)	41.9 (27.8-57.7)
Mali	0.1 (0-0.3)	0.4 (0.2-0.6)	1.5 (0.8-2.5)	4.6 (1.5-11.1)	0.8 (0.5-1.3)	2.4 (1.8-2.9)	6.6 (5.1-8.3)	14.5 (9.6-21.5)
Morocco	0.5 (0.2-0.8)	0.7 (0.5-1.2)	1.3 (0.5-2.8)	2.2 (0.4-6.8)	10.9 (5.5-18.2)	12.9 (8.4-18.2)	16.1 (6.3-31.6)	19.6 (4.4-47.8)
Mozambique	0.1 (0-0.2)	0.1 (0.1-0.2)	0.1 (0.1-0.2)	0.2 (0.1-0.4)	0.1 (0-0.1)	0.2 (0.1-0.3)	0.4 (0.3-0.7)	1 (0.5-2)
Namibia	1.2 (0.4-2.8)	1.4 (0.8-2.2)	1.7 (0.8-3)	2.2 (0.7-5.3)	3.8 (2-6.3)	5.6 (4.1-7.5)	8.5 (5.8-12.2)	11.9 (6.5-19.9)
Nepal	0.4 (0.2-0.7)	1.4 (1-2)	5.4 (3.8-7.2)	14.5 (8.8-22.1)	2.9 (1.1-6.1)	5.5 (3.5-8.6)	10.9 (7.2-15.5)	18.4 (9.2-30.8)
Nicaragua	1.7 (0.1-11.5)	1.4 (0.9-2.2)	5.2 (0.1-38.7)	12.8 (0-91)	1.7 (0.1-9.5)	9.9 (6.3-14.3)	57.9 (8.5-96.3)	81.3 (9-100)

Niger	0.2 (0.1-0.5)	0.1 (0.1-0.2)	0.1 (0-0.1)	0.1 (0-0.2)	0.3 (0.1-0.5)	0.9 (0.6-1.3)	2.9 (1.8-4.6)	7.2 (3.1-14.7)
Nigeria	1.8 (0.8-3.8)	4.1 (2.6-6.2)	9.5 (5.5-14.9)	17.9 (8.2-33.3)	5.4 (2.4-10.3)	13.4 (9.2-19.1)	30.6 (21-41.1)	50.4 (31.1-69.1)
Pakistan	2 (1.1-3.5)	7 (5.1-10)	22.3 (17-28.1)	45.5 (33.8-56.1)	16.6 (11.1-23.2)	28.2 (22.7-34.1)	44 (38-49.8)	57.7 (49.1-65.3)
Peru	2.1 (1.3-3.1)	1.9 (1.5-2.3)	1.7 (1.4-2.1)	1.6 (1.1-2.3)	12.7 (9.3-16.8)	12.1 (10.4-14)	11.5 (9.7-13.5)	11.2 (8.3-14.7)
Philippines	3 (1.8-4.7)	5.7 (4.3-7.4)	10.6 (8.1-13.6)	17 (11.4-24.2)	18.9 (13-25.9)	23.5 (19.5-27.8)	28.9 (24.5-33.8)	33.8 (25.6-41.5)
Rwanda	1.6 (0.4-4.2)	0.8 (0.4-1.3)	0.4 (0.3-0.6)	0.3 (0.1-0.5)	9.3 (3.1-19.6)	6.1 (3.6-9.3)	4.2 (3.1-5.7)	3.3 (1.7-6.1)
Senegal	0.5 (0.3-0.7)	0.9 (0.7-1.1)	1.5 (1.3-1.8)	2.3 (1.8-2.9)	10.2 (7.5-13.9)	9.4 (7.8-11.2)	8.6 (7.6-9.7)	8.1 (6.6-9.8)
Sierra Leone	12.2 (0.2-71.9)	2.7 (0.3-11.2)	0.6 (0.4-0.9)	0.3 (0.1-0.8)	24.3 (0.6-83.7)	14 (1.9-42)	7.7 (5-11.2)	6.4 (1.4-17.1)
South Africa	2.5 (0.8-5.6)	3.1 (1.7-4.9)	4 (2.4-5.6)	5.1 (2.5-8.8)	16.8 (6.7-33.2)	13.5 (7.8-20.8)	11.1 (7.3-15.8)	9.7 (4.9-16.4)
Tanzania	4.6 (2.7-7.2)	6.1 (4.6-8)	8.3 (6.3-10.7)	10.6 (6.8-15.7)	6 (3.5-9.6)	8.6 (6.4-11.2)	12.4 (9.2-15.9)	16.6 (10.4-23.8)
Togo	0.5 (0.2-1.1)	1.3 (0.7-1.9)	3.3 (2.1-4.8)	7.2 (3.3-13.1)	12.1 (5.1-23.9)	16.6 (10.6-25.1)	23 (15.5-32.5)	29.6 (15.7-45.2)
Turkey	1.2 (0.6-2.2)	3.8 (2.7-5.3)	11.9 (8.1-16.7)	26.9 (15.1-41.8)	5 (3.4-7.3)	14 (11.4-16.9)	33.9 (27.4-40.9)	56 (43.3-67.1)
Uganda	11.6 (7.4-16.8)	11.9 (9.4-14.7)	12.5 (10.2-15.1)	13 (9.3-17.7)	30.7 (19.3-42.3)	28.1 (22.4-34.8)	25.7 (20.9-31.3)	24.2 (16.6-33.7)
Vietnam	0.7 (0.1-2.4)	2.7 (1.8-3.9)	14.7 (2.5-45.5)	37.6 (3.1-91.8)	0.6 (0.1-1.9)	3.2 (2.1-4.7)	21.8 (4.5-57.8)	52.5 (6.6-95.9)
Yemen	0.8 (0.4-1.6)	2.3 (1.4-3.5)	6.4 (3.9-9.9)	14.1 (6.9-23.9)	1.9 (0.9-3.4)	5.5 (3.6-8)	15.3 (9.9-23.1)	30.9 (17.3-48)
Zambia	8.9 (6.2-12.5)	7.1 (5.8-8.6)	5.7 (4.2-7.5)	4.9 (2.9-7.5)	18.3 (11.7-27.2)	10.1 (7.7-13.3)	5.4 (3.5-8)	3.3 (1.7-5.9)
Zimbabwe	17 (10.7-24.6)	13.8 (10.8-17.3)	11.3 (8.7-13.9)	9.6 (6.1-13.8)	8.7 (5.1-13.1)	10.2 (7.8-12.8)	12 (9.2-15.5)	13.9 (9.1-20.6)

Note: N/A denotes not application and refers that the indicator has no sufficient data to examine trends

Table S20: Trends in the utilization (%, 95% credible intervals) of delivery services in public facilities in low- and middle-income countries by education

	Below secondary education				Secondary+ education			
**Country**	**1990**	**2000**	**2010**	**2018**	**1990**	**2000**	**2010**	**2018**
Afghanistan	67.1 (6.3-98.9)	59.9 (17.1-91.9)	47.9 (34.4-62.4)	37.7 (18.1-62.4)	33.8 (0.8-91.9)	44.8 (8.1-84.5)	61.4 (45.9-73.6)	73.1 (51.8-88.3)
Albania	89.8 (55.6-99)	94.8 (85.1-98.7)	97.3 (95.5-98.4)	98.2 (96.9-99)	100 (99.9-100)	99.9 (99.6-100)	98.8 (98.1-99.3)	92.4 (87.4-96)
Angola	69.8 (26.7-95.1)	57.2 (31.5-80.2)	41.1 (31.1-51.7)	29.4 (16.4-47.7)	99.1 (95.9-99.9)	97.2 (92.5-99.3)	88.7 (83.1-92.7)	68.2 (48.9-82.1)
Armenia	26.8 (3-72.9)	96.4 (90.8-99)	100 (99.9-100)	100 (100-100)	92.6 (82.4-98.1)	94.3 (90.6-97)	95.3 (93.3-96.9)	95.7 (91.5-98.1)
Bangladesh	0.7 (0.4-1.1)	2.1 (1.6-2.7)	6.7 (4.9-8.8)	15.9 (9.5-24.4)	8.2 (5.8-11.2)	11.1 (9.4-12.8)	14.9 (12.2-17.9)	18.8 (13.5-25.4)
Benin	53 (37.5-66.7)	61.6 (53.2-68.8)	69.6 (62.8-75.2)	75 (64.8-83.2)	69 (59.1-78)	70.2 (65-75.1)	71.2 (67.1-74.8)	72 (65.5-78.3)
Bolivia	14.6 (8.3-23.4)	32.2 (26.9-37.8)	57.3 (44.5-69.8)	74.8 (54.4-88.6)	45.6 (31-62.3)	61.7 (54.7-68.5)	75.4 (63-84.8)	83.1 (65.2-93.3)
Brazil	69.8 (51.6-83.9)	88.6 (71.1-96.9)	93.9 (62.7-99.9)	95.2 (54.2-100)	72.1 (56.3-83.6)	77.4 (54.7-92.5)	78.2 (24.7-99)	77.9 (10.1-99.8)
Burkina Faso	28.6 (15.8-44.3)	41.4 (32.5-50)	55.8 (38.9-71.8)	66.1 (41-85.8)	83.3 (74-90.3)	85.8 (81.9-89.2)	87.5 (81-92.4)	88.5 (77.6-95.1)
Burundi	3 (0.1-19.5)	13.6 (2.3-41.1)	55.1 (40.1-68.5)	87.8 (79.1-93.7)	40.4 (3.2-90.6)	60.8 (23.7-89.1)	81.2 (70.4-88.8)	90.4 (83-95)
Cambodia	0.3 (0.1-0.7)	4.7 (3.1-6.8)	44.3 (37.5-50.6)	87.9 (80.5-92.6)	8.4 (3.4-17.4)	24.3 (16.6-33)	54.2 (45.8-61.9)	76.9 (65.3-86.3)
Cameroon	41.6 (31-53.7)	37.1 (30.2-43.9)	32.9 (23.4-43.1)	29.9 (17-44.8)	65.6 (53.8-76.7)	60.2 (53.4-67.1)	54.3 (43.5-64.8)	49.6 (32.3-66.3)
Chad	5.8 (2.5-10.9)	9.1 (6.1-12.6)	14.6 (10.4-19.7)	21 (11.6-32.4)	48 (31.9-63.6)	50.1 (41.2-59.4)	52.1 (43.3-61.6)	53.7 (37.8-68.3)
Colombia	58.6 (45-71.6)	69.3 (61.8-76)	78.2 (70-85.3)	83.5 (72.3-91.6)	65.5 (44.8-82.9)	76.8 (66.1-84.5)	84.8 (74.8-91.7)	88.9 (75.6-96)
Comoros	26.7 (13-45.7)	46.4 (35.2-57.8)	67.8 (56.4-77.9)	80.7 (65.1-90.7)	67.7 (46.3-83.6)	77.6 (68.5-84.8)	84.8 (77-90.7)	88.7 (78.2-95.2)
Republic of the Congo	64 (16.8-95.6)	67.1 (42.5-87)	69.3 (60.2-77.6)	69.4 (43.7-87.8)	76.1 (32.6-97.3)	79.8 (59-92.6)	81.5 (73.3-88.1)	80.8 (56.7-93.8)
Democratic Republic of the Congo	25.6 (2-77.8)	37.7 (13.3-69)	56.1 (45.7-66.3)	69.7 (46.7-86.8)	30.3 (2.3-81.3)	42.8 (14-74.1)	59.9 (49.7-69.7)	71.9 (50.8-87.4)
Cote d'Ivoire	39.4 (26-53.5)	46.9 (39.5-54.8)	54.6 (44-64.9)	60.5 (43.1-76.2)	79.9 (69.7-87.4)	76.4 (71-80.9)	72 (64.4-79.1)	68 (54-80.3)
Dominican Republic	77.7 (68.3-84.6)	85.1 (81.5-88.4)	90.2 (86.4-93.2)	93 (88.2-96.2)	54.2 (42.7-65.4)	59.9 (54-65.3)	65.3 (55.9-72.9)	69.2 (54.8-80.3)
Egypt	13.5 (10.7-17.2)	20.1 (17.9-22.4)	28.8 (25.2-33)	37.3 (30.3-45.2)	27.7 (23.7-31.9)	26 (23.9-28.1)	24.5 (21.7-27.1)	23.3 (18.8-28)
Ethiopia	0.5 (0.1-1.2)	2 (1.1-3.5)	9.5 (6.3-13)	28.5 (15.4-42.9)	20.7 (8.8-37.2)	38 (27.3-49.5)	60.1 (52.7-66.6)	75.4 (64-84.1)
Gabon	69.4 (43.1-89)	67.6 (54.3-79.7)	64.8 (51.6-74.9)	62.1 (37.9-81.1)	75.6 (52.2-91.1)	73.1 (60.1-83.2)	69.5 (57.8-79.1)	65.8 (43.8-83.1)
Ghana	20.4 (12.4-31.3)	30.7 (23.9-37.4)	43.7 (33.2-54.1)	54.7 (37.1-70.4)	47.3 (31.3-63.7)	57.1 (48.9-65.7)	66.4 (56.4-75.6)	72.7 (57.9-85.7)
Guatemala	19.7 (12.8-27.3)	31.5 (25.6-38.2)	46.5 (38.3-55.9)	59.1 (46.3-71.3)	54.6 (39.3-68.7)	61.5 (53.1-69.4)	68 (59.1-75.9)	72.5 (58.9-83.9)
Guinea	21.7 (10.1-39.7)	26.1 (19.5-35.2)	32 (23.7-41.1)	37.4 (21.4-56.3)	74.7 (53.6-88.1)	67.8 (57.7-76)	59.2 (50.2-67.7)	51.8 (33.5-69.5)
Haiti	6.2 (3.4-10)	9.9 (7.2-12.8)	15.7 (12-20)	22.3 (14.9-31)	24.4 (15.3-36)	32.1 (25.9-39.2)	41.1 (34.5-47.9)	48.9 (37.5-59.9)
Honduras	21.6 (1.1-73.9)	40.9 (14.9-71.6)	70 (59.5-78.7)	84.9 (64-96)	54.3 (7.1-94.3)	72.1 (41.7-91.6)	86.1 (80.3-90.3)	91.1 (76.6-97.8)
India	6.5 (3-11.4)	14.7 (9.9-20.7)	30.8 (20.7-40.7)	48.6 (30.5-65.8)	21.4 (11.8-32.5)	30 (22.3-37.7)	40.8 (30.5-51.3)	50.1 (34.3-66.6)
Indonesia	3.4 (2.1-5.1)	7.4 (5.7-9.4)	15.3 (11.8-19.9)	26 (17.4-36)	15.4 (10.2-21.8)	17.5 (13.8-22.3)	20.1 (15-26.3)	22.4 (14.4-32.7)
Jordan	68 (54.6-79.7)	70.4 (62.4-77.3)	72.4 (63.4-79.3)	73.8 (60.7-83.1)	57 (50.9-62)	60.3 (56.8-63.3)	63.5 (60.2-67.1)	65.9 (61-71)
Kazakhstan	100 (100-100)	100 (100-100)	100 (99.8-100)	99.8 (99.1-100)	98.3 (94.3-99.7)	98.3 (96.8-99.2)	96.1 (78.6-99.9)	92.3 (39.5-100)
Kenya	22.5 (13-34)	27.3 (21.4-34)	33.3 (25.3-42.9)	38.5 (24.5-54.3)	44.2 (30.7-58.2)	47.5 (40.1-54.6)	51 (42.3-59.4)	53.7 (39.4-66.9)
Kyrgyz Republic	100 (100-100)	100 (100-100)	100 (100-100)	100 (100-100)	91.7 (84.1-96.8)	97.1 (95.6-98.2)	99 (98.3-99.4)	99.5 (98.9-99.8)
Lesotho	18.6 (3.2-52.2)	32 (16.1-52.6)	52.9 (44-61.5)	69.1 (48.9-84.3)	41.4 (13.9-74.9)	60 (41.6-76.1)	77.1 (70.6-82.5)	86 (73.2-93.2)
Liberia	3 (0.3-12.3)	10.7 (3.5-24.1)	35.9 (28.1-44.3)	67.3 (52.9-79.3)	31.4 (6.7-71.2)	40.1 (19.7-62.9)	50.8 (42.4-58.8)	59.5 (43.7-72.5)
Madagascar	39.5 (26.3-52.2)	31.1 (24.7-38.4)	24.2 (14.4-35.6)	19.9 (8.3-36.5)	45.9 (33.1-57.9)	46.9 (40-54)	47.9 (37.2-59.6)	48.8 (31.3-66.8)
Malawi	26.7 (15.7-40)	43.5 (34.4-52.9)	62.3 (52.4-70.9)	75 (61.9-84.8)	57.1 (40.8-73.5)	64.4 (55.7-72.9)	71 (62.5-78.6)	75.4 (62.3-85.5)
Maldives	83.4 (37.6-99.1)	87.8 (65.2-97.4)	90.6 (85-94.3)	91.3 (84.5-95.5)	89.6 (58.9-99.3)	87 (66.5-97)	81.7 (72.4-88.4)	74.4 (59.9-84.4)
Mali	16.6 (7.3-31)	29 (21.4-38.8)	46.7 (35-58)	61.4 (40.5-78.2)	69.4 (50.2-84.1)	74.2 (66.1-81.3)	78.1 (69.3-85)	80.4 (65.4-90.6)
Morocco	19.7 (10.9-31.7)	42.6 (32.4-53.9)	69.1 (48.9-84.5)	83.4 (59.1-95.7)	53.9 (36.7-69.6)	63.3 (53.4-72.5)	71.3 (53.2-85.8)	76.1 (48.3-93)
Mozambique	34.7 (20.1-52)	45 (36.5-54.7)	56 (48.3-63.8)	64.4 (52-76.5)	94 (88.7-97.4)	93.5 (90.6-95.7)	92.8 (90.5-94.7)	92.1 (87.4-95.4)
Namibia	54 (42.4-64.9)	61.7 (55.8-67.5)	68.8 (61.1-75.3)	73.8 (61.4-82.8)	82.3 (73.8-88.6)	84.4 (79.9-88.2)	86 (81-90)	87 (79-92.4)

Nepal	1.5 (0.7-2.7)	5.4 (3.7-7.6)	18.7 (14.5-23.8)	41.2 (28.9-54)	16.3 (8.3-27)	28.2 (20.9-36.5)	45 (36-53.1)	59.4 (44.6-71.7)
Nicaragua	54.4 (8-95.7)	54.9 (43.7-65.8)	53.1 (4.6-95.8)	52 (0.5-99.5)	82 (34.9-98.9)	77.8 (68.8-84.7)	61.8 (9.2-97)	52 (0.6-99.4)
Niger	12.5 (7.3-19.9)	17.5 (13.3-22.6)	24.5 (17.2-33.4)	31.4 (17.8-48.1)	73.7 (60.5-83.5)	73.6 (67.5-79.5)	73.2 (64.5-81.4)	72.6 (58.1-84.6)
Nigeria	22 (12.9-33.2)	17.3 (12.9-23)	13.7 (9.7-18.7)	11.4 (6.4-19.2)	70.5 (55.4-83.4)	54.4 (44.3-64.2)	37 (27.2-47)	25.2 (14.4-39.2)
Pakistan	4.4 (2.3-7.4)	7.6 (5.3-10.6)	13 (9.9-16.6)	19.6 (13.5-27.1)	25.4 (16-37.6)	24.3 (18.3-31.1)	23.4 (18.2-29.1)	22.8 (15.7-31.8)
Peru	13.4 (9.5-18.7)	32.5 (28-37)	60.3 (55.7-64.9)	79.1 (72.8-84.4)	56.8 (51-62.9)	71.2 (68.6-73.8)	82.4 (80.3-84.2)	88.5 (86.1-90.7)
Philippines	6.1 (3-10.8)	12.6 (8.8-17.4)	24.7 (18.9-31.5)	39 (26.6-51.8)	17.9 (11.4-26)	29.2 (23.6-35.8)	44.1 (37.8-51.1)	56.9 (46.6-67.6)
Rwanda	9.3 (3.9-18.8)	30.7 (21.4-41)	66.8 (55.5-76.9)	86.7 (75.2-93.7)	49.2 (31.1-66.5)	68.7 (60.6-76.6)	83.3 (77.6-88)	90.4 (83.9-95.1)
Senegal	36.4 (31.2-41.4)	50.8 (47.5-54)	65.1 (63.1-67)	74.9 (72.5-77.2)	60.3 (48.8-71)	70.8 (65.1-76.3)	79.5 (76.2-82.4)	84.8 (81-88.2)
Sierra Leone	0.7 (0-5.3)	3.7 (0.4-15.4)	32.4 (24.7-42.1)	80.6 (54.1-94.5)	0.8 (0-5.4)	5.2 (0.6-17.8)	46.6 (36.9-57.3)	89.8 (73-97.5)
South Africa	57.8 (36.1-76.9)	77.3 (67.4-85.5)	89.5 (84.6-93)	94.5 (90.3-97.1)	74.6 (55.4-88.5)	80.9 (71.4-88.2)	85.6 (79.6-90.4)	88.4 (80.6-93.7)
Tajikistan	13.5 (0.1-81.5)	27.6 (2.6-78.8)	60.5 (38.3-79.4)	84.7 (73.1-92.3)	19.9 (0.2-81.5)	39.6 (4.8-83.1)	73 (54-85.3)	90.3 (82.7-95.1)
Tanzania	38.5 (28.9-48.9)	41 (35.2-47.1)	43.6 (37.2-49.7)	45.8 (36-56.1)	70.6 (62.5-77.5)	70.5 (66.3-74.4)	70.3 (65.8-74.7)	70.1 (62.4-76.8)
Timor-Leste	0.8 (0-4.8)	2.2 (0.2-9.4)	11.1 (6.2-17.7)	39 (21.6-58.6)	5.4 (0.1-30.7)	13.6 (2-40)	38.6 (25.7-51.8)	68.8 (52.1-82.7)
Togo	35.2 (17.4-59.2)	44.5 (31.9-57.3)	54.6 (43.3-65.8)	62.3 (45.1-78.5)	74.2 (51.5-87.7)	75.5 (64.3-84)	76.3 (67-84.1)	76.5 (61.3-88.3)
Turkey	55.4 (41.5-67.2)	63.3 (56.8-69.1)	70.4 (63.2-78)	75.3 (64.2-86)	80.6 (72.5-87)	71.6 (66.3-76.2)	60.1 (52-67.5)	50 (35.6-62.4)
Uganda	10.6 (5.9-17.6)	21.9 (16.7-28.3)	40.5 (33.1-49.1)	57.9 (45.1-71.5)	32.3 (28.3-36.7)	44.2 (41.9-46.7)	56.7 (54.6-59.1)	66.3 (62.7-69.4)
Vietnam	21.1 (4.2-52.6)	50.6 (40-61)	79.2 (42.7-96.3)	88.6 (35.6-99.7)	47.9 (15.5-81.6)	79.2 (71.4-85.6)	92.4 (74.8-99)	95.5 (67.2-99.9)
Yemen	10.4 (5.5-17.6)	13.5 (9.4-19.7)	17.8 (11.6-26.5)	22.1 (12.3-36.6)	74.3 (60.1-85.3)	55.1 (44.1-65.7)	34.2 (23.4-46.3)	20.9 (11.1-35.5)
Zambia	23.7 (14.1-35)	33.4 (26.3-40.8)	45 (34.3-56)	54.7 (37.9-70.7)	57.5 (46.3-68.2)	66.2 (60.3-71.8)	73.9 (66.4-80.2)	79 (68.4-87)
Zimbabwe	43.9 (28.4-59.8)	46.1 (37.3-55)	48.4 (39.5-57.7)	50.2 (35.1-65.3)	72.3 (60-82.7)	70.7 (63.8-77.1)	68.7 (61.3-75.5)	67 (54.1-77.8)

Table S21: Trends in the utilization (%, 95% credible intervals) of delivery services in private facilities in low- and middle-income countries by education

	Below secondary education				Secondary+ education			
**Country**	**1990**	**2000**	**2010**	**2018**	**1990**	**2000**	**2010**	**2018**
Afghanistan	53.3 (2.6-98.3)	28.5 (3.6-73.1)	8.6 (4.7-14.3)	3.5 (1.1-8)	57.1 (2.8-98.6)	36.8 (5.6-81.9)	16 (9.4-25.4)	8.2 (2.9-18.7)
Albania	N/A	N/A	N/A	N/A	0 (0-0)	0.1 (0-0.2)	1 (0.5-1.6)	7.5 (4-12.6)
Angola	4 (0.3-15.4)	1.9 (0.5-4.8)	1 (0.6-1.5)	0.7 (0.3-1.3)	2 (0.2-8.1)	2.3 (0.7-5.5)	3 (1.9-4.5)	4.3 (1.9-8)
Armenia	N/A	N/A	N/A	N/A	0 (0-0)	0.1 (0.1-0.3)	2 (1.2-3.2)	16.3 (6.6-31)
Bangladesh	0.1 (0.1-0.2)	0.7 (0.6-1)	4.5 (3.3-6.2)	17.3 (10.6-27)	4.7 (2.7-7.4)	10 (7.7-12.6)	20.6 (15.7-25.7)	33.8 (22.6-46.3)
Benin	9.4 (5.5-14.4)	10.3 (7.7-13.1)	11.4 (9.2-14.1)	12.5 (8.7-17.3)	27.5 (18-39.3)	27.2 (21.2-33.7)	27 (21.7-32.3)	27 (19.5-35.8)
Bolivia	8 (3.8-14.4)	5.2 (3.7-6.8)	3.5 (1.8-6.1)	2.7 (0.9-6.6)	25.4 (14.1-38.7)	21.1 (16.5-26.4)	17.9 (11.1-26.6)	16.1 (6.4-32.5)
Brazil	3.8 (1.7-7.5)	4.5 (1.3-11.3)	8 (0.3-42.9)	12 (0.1-77.7)	24.2 (13.5-38.1)	21.9 (7.1-42.1)	23.3 (1.1-71.8)	24.9 (0.2-88.9)
Burkina Faso	0.5 (0.2-1)	0.5 (0.3-0.7)	0.5 (0.3-1)	0.6 (0.2-1.5)	5.7 (2.5-10.6)	7.5 (5.1-10.6)	10.6 (5.7-18.1)	14.1 (5.1-30.3)
Burundi	24.3 (1.3-78.5)	11.6 (2-35.7)	4.7 (2.5-7.9)	2.6 (1.3-4.6)	28.6 (1.5-83.1)	17.8 (3.2-49.4)	10 (5.2-16.9)	6.9 (3.4-12)
Cambodia	0.2 (0.1-0.4)	1.1 (0.8-1.6)	5.9 (4.5-7.5)	19.9 (12.2-28.3)	2.9 (0.9-6.6)	7.7 (4.7-11.7)	20.2 (15.2-25.7)	38.5 (22.9-55.9)
Cameroon	10.2 (7.2-13.7)	12.4 (10.4-14.6)	15.1 (11.7-19.6)	17.8 (11.4-26)	20.6 (15.1-27.3)	27.7 (23.6-32)	36.4 (29-43.5)	44.1 (31.7-55.2)
Chad	1.2 (0.4-2.9)	0.8 (0.5-1.3)	0.6 (0.3-0.9)	0.5 (0.2-1)	3.5 (1.4-7.3)	3.6 (2.3-5.6)	4.1 (2.6-6.1)	4.6 (2.1-8.8)
Colombia	8.6 (2.5-20.3)	2.6 (1.3-4.8)	0.8 (0.4-1.7)	0.4 (0.1-1.1)	28.9 (13.8-50.3)	14.9 (8.7-23.6)	7.3 (3.3-13.2)	4.3 (1.2-10.6)
Comoros	0 (0-0.1)	0.2 (0.1-0.3)	0.8 (0.5-1.3)	2.7 (1.1-5.3)	0 (0-0)	0 (0-0)	0 (0-0)	0 (0-0)
Republic of the Congo	3.4 (0.3-16.2)	5.6 (1.9-13.3)	12.1 (7.5-17.9)	23.6 (6.9-46)	3.9 (0.3-19)	6.3 (2-15.5)	13.1 (8.8-18.2)	24.9 (8.3-49.5)
Democratic Republic of the Congo	36.4 (4.5-84.3)	23.2 (7.4-47.9)	13.5 (9.2-18.7)	9.5 (3.5-21.2)	53.5 (9.1-91.9)	41.8 (16.4-71)	29.5 (21.7-38.5)	22.4 (9.3-42.2)
Cote d'Ivoire	0.2 (0.1-0.5)	0.8 (0.5-1.2)	2.9 (1.6-4.8)	8.2 (2.8-18.1)	3.2 (1.6-5.6)	5.3 (3.8-7.1)	9.1 (6.1-13.3)	14 (7.2-25.1)
Dominican Republic	14.4 (9.7-20.4)	10.4 (8.3-12.8)	7.6 (5.2-10.7)	5.9 (3.2-10)	45 (34.7-56.4)	39.6 (34-46)	34.5 (25.9-43.3)	30.8 (18.6-44.3)
Egypt	4.8 (3.6-6.1)	14.7 (13.1-16.5)	37.5 (33.1-42.4)	61.9 (54.3-69.3)	23.3 (19.5-28.1)	40.9 (38.1-43.8)	61.2 (56.8-65.1)	75.2 (69.4-79.8)
Ethiopia	0 (0-0)	0.1 (0-0.1)	0.3 (0.2-0.4)	1 (0.5-1.8)	1 (0.2-2.8)	2.5 (1.2-4.7)	6.7 (4.4-9.5)	14.7 (7.7-25.5)
Gabon	8.2 (2.3-21)	12.2 (6.8-20)	19.3 (12.3-27.6)	27.6 (13.2-46.5)	15.2 (4.4-34.3)	19.5 (10.7-30.4)	26 (17.4-37)	32.8 (15.7-55.9)
Ghana	10.9 (6.5-16.6)	7.4 (5.6-9.6)	5.1 (3.7-7)	3.8 (2.2-6.3)	21.9 (16.2-28.4)	16.9 (14.2-19.6)	12.9 (10.5-16)	10.4 (7.3-14.7)
Guatemala	3.2 (1.6-5.7)	3.2 (2.1-4.6)	3.4 (2.1-5.2)	3.6 (1.7-6.7)	30.2 (19.7-42.8)	25.9 (19.6-32.9)	22.1 (15.6-28.4)	19.6 (11.3-29.8)
Guinea	0.4 (0.1-1)	1 (0.6-1.5)	2.6 (1.6-4)	6.1 (2.3-13.1)	1.1 (0.4-2.7)	4.1 (2.7-6.1)	14.8 (9.8-20.8)	35.1 (18.4-55.3)
Haiti	9.5 (4.1-18.8)	5.8 (3.6-8.5)	3.7 (2.4-5.4)	2.6 (1.3-4.9)	42.8 (28-58.3)	28.7 (22.1-35.7)	17.7 (13.7-22.6)	11.7 (7.3-17.4)
Honduras	9.5 (0.4-49.1)	4.6 (1.1-13.4)	2.6 (1.7-3.8)	2.2 (0.5-6.2)	36.3 (3.8-85.8)	20.5 (6-45.2)	10.2 (6.8-14.7)	6.8 (1.7-18.6)
India	5.1 (3.2-7.6)	7.7 (5.9-9.9)	11.7 (8.6-15.4)	16.2 (10-23.5)	36.1 (26.2-46.8)	36.5 (30.2-42.7)	37.1 (30.1-43.6)	37.6 (26.7-47.7)
Indonesia	4.4 (2.7-6.5)	10.7 (8.2-13.6)	24.2 (18.2-30.9)	41.1 (29.3-54.9)	23.9 (16.9-31.7)	36.1 (30.8-41.7)	50.7 (43.4-58.3)	62.2 (51.1-72.8)
Jordan	12.6 (7.5-19.2)	16.7 (12.3-21.6)	21.9 (15.9-28.3)	27.1 (17.8-38.6)	33.7 (25.9-41.2)	34.4 (29.4-39.4)	35.3 (29.8-40.4)	36 (28.3-43.6)
Kenya	6.4 (3.8-10.1)	7.6 (5.8-9.7)	9.3 (6.7-12.5)	10.9 (6.4-17.3)	22.1 (14.2-31.6)	24.3 (19.3-29.3)	26.9 (20.2-33.9)	29.3 (18.6-41.7)
Lesotho	2.5 (0.3-11)	1.9 (0.7-4.7)	1.9 (1.3-2.7)	2.1 (0.7-4.9)	0.9 (0.1-3.9)	1.7 (0.6-4.1)	4.2 (2.8-6)	9.5 (3.5-19.7)
Liberia	9.7 (1.5-30.1)	8.9 (3.3-18.6)	8.7 (6.2-11.7)	9.2 (5.3-14.4)	17 (2.9-47.1)	18.5 (7.1-36.1)	21.3 (15.8-27.7)	24.8 (15.5-35.5)
Madagascar	0.5 (0.2-0.8)	0.9 (0.6-1.2)	1.8 (0.9-3.1)	3.2 (1-7.3)	3.2 (2.3-4.6)	5.6 (4.6-6.6)	9.5 (6.8-12.5)	14.4 (8.7-21.8)
Malawi	16.8 (9-27.4)	16.1 (11.5-22.4)	15.8 (11.5-21.7)	15.9 (8.9-25.9)	31.8 (20.7-45.1)	26.1 (19.8-32.2)	21.2 (15.8-27.4)	18 (10.6-27.5)
Maldives	3.2 (0.1-17.4)	3.8 (0.7-11.3)	5.8 (3.4-9.1)	9.4 (4.8-17.5)	10.4 (0.6-46.1)	12.8 (3-34)	18 (11.2-26.7)	25.4 (14.1-37.8)
Mali	0.2 (0.1-0.5)	0.7 (0.4-1)	2.1 (1.2-3.3)	5.2 (2.1-10.9)	3.7 (1.7-6.8)	6 (4.2-8)	10 (6.6-14.1)	15 (7.8-24.6)
Morocco	1.4 (0.7-2.8)	2.4 (1.6-3.5)	4.4 (1.6-9.6)	7.5 (1.6-21.8)	30.1 (17.9-45.3)	27.2 (18.9-36.7)	25.3 (11-44.3)	24.5 (5.7-53.3)
Mozambique	0.1 (0-0.2)	0.1 (0.1-0.2)	0.2 (0.1-0.2)	0.2 (0.1-0.4)	5.1 (0.6-19.9)	2.1 (0.7-4.8)	1 (0.6-1.5)	0.6 (0.2-1.4)
Namibia	1.9 (0.6-4.2)	0.7 (0.4-1.1)	0.3 (0.1-0.5)	0.1 (0-0.3)	4.4 (2.6-7.2)	5.5 (4.1-7.2)	7 (5-9.7)	8.7 (4.8-14.1)
Nepal	0.2 (0.1-0.4)	0.9 (0.6-1.2)	3.5 (2.6-4.6)	10.4 (6.1-16.3)	4.9 (2.4-8.7)	7.7 (5.5-10.5)	12.4 (9.4-15.8)	18 (11.5-26.1)
Nicaragua	0.5 (0-2.4)	1.4 (0.8-2.1)	15.6 (0.5-66)	42.6 (0.3-98.9)	4 (0.1-22.9)	13.9 (9.4-19.4)	54.9 (6.9-96.2)	75.7 (3.3-99.9)

Niger	0.2 (0.1-0.4)	0.3 (0.2-0.4)	0.3 (0.2-0.5)	0.3 (0.1-0.7)	1.9 (0.9-3.6)	2.6 (1.8-3.9)	3.8 (2.2-6)	5.3 (2.2-10.8)
Nigeria	2 (0.9-3.8)	3.8 (2.4-5.4)	7.2 (4.4-10.9)	12.2 (5.8-21.7)	6.3 (2.7-12.4)	16 (10.5-23.2)	36 (23.5-48.6)	56.9 (36.4-74.9)
Pakistan	3.5 (2-5.6)	9.2 (6.6-12.1)	22.2 (17.7-27.4)	39.6 (30.5-48)	27 (17.4-37.5)	39.1 (31.2-47.2)	53 (44.9-59.6)	63.8 (53.6-72.2)
Peru	2.5 (1.8-3.4)	2.3 (1.9-2.7)	2.1 (1.7-2.5)	1.9 (1.4-2.6)	14.4 (10.5-19.6)	12.5 (10.5-14.8)	10.9 (9-13.1)	9.8 (7-13.1)
Philippines	1.8 (1.1-2.8)	3.2 (2.4-4)	5.7 (4.4-7.1)	8.9 (6.1-12.4)	16.8 (11.6-22.5)	20.1 (16.5-23.8)	24 (20-28.3)	27.6 (21-35.3)
Rwanda	3.5 (0.8-9.5)	1.1 (0.5-1.8)	0.3 (0.2-0.5)	0.2 (0.1-0.3)	12.3 (4.9-24.3)	8.4 (5.5-12.1)	5.9 (4.6-7.6)	4.5 (2.6-7.6)
Senegal	2.4 (1.9-3)	2.6 (2.3-3)	3 (2.7-3.3)	3.3 (2.8-3.7)	30.1 (19.9-43.3)	20.9 (16.1-26.9)	14 (11.5-17.1)	10.1 (7.3-13.8)
Sierra Leone	3.9 (0-27.9)	1.8 (0.2-7.8)	1.3 (0.8-1.9)	1.6 (0.4-4.5)	81.5 (16.1-99.6)	47.8 (12.5-84.4)	9.4 (6.1-13.7)	2.1 (0.5-5.9)
South Africa	2.4 (0.9-5.1)	1.1 (0.6-1.7)	0.5 (0.3-0.7)	0.3 (0.1-0.5)	14.8 (6.8-27.9)	11.9 (7.5-18.4)	9.8 (6.5-14)	8.5 (4.6-14.5)
Tanzania	4.8 (3-7.4)	6.3 (4.9-8)	8.5 (6.4-10.9)	10.8 (7-15.4)	10.2 (6-15.8)	12.5 (9.4-16.2)	15.4 (11.6-20.1)	18.3 (11.6-26.6)
Togo	2.2 (0.7-4.7)	4.2 (2.3-6.6)	8.2 (5.2-12.6)	14.1 (6.7-25.7)	10 (4-21.7)	12.6 (8-18.8)	16.5 (10.7-24.1)	20.7 (10.6-34.5)
Turkey	2.4 (1.6-3.7)	7.3 (5.8-9.2)	20.6 (16-26.1)	40.1 (28.7-51.9)	9.1 (5.9-13.5)	20.3 (16.3-24.4)	39.8 (32.1-47.5)	58.4 (44.8-70.8)
Uganda	12.1 (8-17.1)	11.8 (9.5-14.3)	11.6 (9.5-14)	11.5 (8.1-15.3)	32.1 (21.7-43.6)	28.7 (23.1-34.9)	25.6 (20.8-30.9)	23.4 (16.2-32.2)
Vietnam	1.1 (0.1-4.2)	3.2 (2.1-4.7)	14.8 (2-50.4)	34.8 (1.5-92.4)	0.5 (0.1-1.8)	2.5 (1.7-3.7)	17.7 (3.3-56.5)	45.8 (4.6-96.2)
Yemen	1 (0.5-1.8)	2.7 (1.7-4.1)	7.4 (4.5-11.7)	15.9 (8.3-27.6)	3.5 (1.7-6.1)	8.2 (5.4-11.7)	18.5 (11.7-26.4)	32.4 (18.8-49.5)
Zambia	11 (7.7-15)	7.3 (5.9-8.9)	4.9 (3.6-6.3)	3.5 (2.2-5.3)	20.1 (13.1-28.9)	12.2 (9.4-15.4)	7.3 (5-10.2)	4.9 (2.5-8.3)
Zimbabwe	15 (8.5-23.8)	11.4 (8.4-14.9)	8.6 (6.4-11.1)	7 (4.1-10.8)	15.4 (9.7-21.7)	14 (11-17.5)	13 (10.2-15.8)	12.2 (8.1-16.8)

Note: N/A denotes not application and refers that the indicator has no sufficient data to examine trends

Table S22: Trends in the utilization (%, 95% credible intervals) of delivery services in public facilities in low- and middle-income countries by age

	Adolescent, 15-19 years of age				Adult, 20-49 years of age			
**Country**	**1990**	**2000**	**2010**	**2018**	**1990**	**2000**	**2010**	**2018**
Afghanistan	22.7 (0.5-87.8)	27.3 (4.2-71.8)	36.7 (24.5-50.5)	48.2 (24.3-70.2)	7.3 (0.1-52.1)	12.5 (1.1-44.3)	28.8 (18.1-41.6)	53.8 (30.3-76.1)
Albania	0 (0-0)	2.8 (0.7-8.7)	96.8 (94.9-98.2)	100 (100-100)	99.4 (97.5-100)	99 (97-99.8)	97.7 (96.4-98.6)	94.9 (91.3-97.2)
Angola	70.3 (26.7-95.4)	63.4 (36.4-84.6)	53.9 (42.7-64.1)	45.5 (28.4-64)	54.2 (12-91)	51.2 (25.1-77.4)	47.6 (37.9-57.6)	44.8 (27.7-62.9)
Armenia	89.1 (68.4-97.9)	93.3 (86.5-97)	95.5 (93-97.4)	96.3 (91.6-98.8)	92.3 (80.6-98)	94.4 (90.1-97.1)	95.6 (93.5-97.1)	96.1 (92.4-98.3)
Bangladesh	1.6 (0.9-2.6)	4.2 (3.3-5.4)	10.9 (8.2-14.1)	22 (14-31.8)	1.7 (1-2.6)	4.2 (3.3-5.2)	10.4 (8.1-13.1)	20.4 (13.7-28.3)
Benin	56.7 (41.2-72.5)	65.7 (57.4-73.7)	73.7 (67.9-79.1)	79 (69.5-86.2)	53.8 (40.5-67.4)	62 (53.9-69.4)	69.5 (63.2-75.2)	74.7 (65.9-82.4)
Bolivia	34.5 (20.4-52.6)	52.6 (43.7-60.3)	70 (54.8-81.4)	80.1 (58.3-92.5)	24.9 (15.7-36.4)	42.9 (36.8-49.3)	63.3 (51.7-73.5)	76.6 (59.3-88.2)
Brazil	84.1 (72.8-91.7)	93.3 (83.2-98.2)	95.6 (74-99.9)	96 (64.8-100)	69.9 (54.5-83.1)	81.3 (57.4-93.9)	85.4 (33.9-99.4)	86.7 (18.4-99.9)
Burkina Faso	32.4 (18.3-49.2)	48.5 (39.3-57.2)	65 (50-77.8)	75.6 (52.2-89.8)	29.6 (16.5-46.3)	42.5 (33.6-51.8)	56.9 (40.8-70.9)	67 (41.9-85.2)
Burundi	20.4 (0.5-74.7)	42.3 (8.8-79.6)	74.3 (61-84.5)	90 (82.6-95.3)	3.5 (0.1-22.2)	15 (2.8-44.1)	56.7 (42.4-71)	88.1 (79.2-93.8)
Cambodia	1.8 (0.5-4.6)	11 (6-17.3)	48.2 (38.3-58)	82 (66.7-91.3)	0.8 (0.3-1.5)	7.3 (4.8-10.5)	46.4 (39.1-53.1)	85.2 (76.9-91)
Cameroon	48.5 (37-60.6)	45.2 (38.9-52.1)	42.1 (32.2-53.2)	39.7 (24.2-57.8)	46.4 (35.4-58.1)	42.9 (36.8-49.8)	39.7 (29.4-50.4)	37.3 (22.1-53.6)
Chad	8.6 (3.8-16.1)	13.2 (8.8-18.5)	20.3 (14.4-27.5)	28.2 (16.6-42.7)	6.4 (3.1-11.8)	10.4 (7.4-14)	17 (12.3-22.3)	24.7 (13.8-37.3)
Colombia	72.1 (55.7-85.5)	80.2 (72.4-86)	86.1 (78.3-91.6)	89.3 (78.1-95.4)	62.6 (44.3-78.3)	72.1 (62.2-80.7)	79.6 (68.5-87.7)	84 (70.6-93.2)
Comoros	31.8 (15.7-52)	53.4 (41-65.6)	74.1 (63.7-83.1)	85.2 (73-93)	30.2 (14.8-48.9)	51.2 (38.4-63)	72.1 (59.9-81.6)	83.8 (67.6-93.1)
Republic of the Congo	88 (54.8-99.2)	84.3 (68-95.2)	76.3 (67.9-83.6)	66.2 (39.4-87.4)	64 (20.1-95)	71.2 (48.5-88.1)	77 (69.2-84)	79.4 (55.8-92.4)
Democratic Republic of the Congo	14.5 (1-56.9)	29.3 (9.3-59.7)	56.6 (45.3-65.9)	76.7 (54.7-90.6)	27.3 (2.9-76.3)	39.6 (15.4-70.7)	57.4 (46.9-67.6)	70.5 (46-86.2)
Cote d'Ivoire	45.6 (38.1-53)	48.8 (44.6-52.9)	52 (45.3-57.8)	54.5 (44.1-63.8)	42.1 (28.4-56.3)	49.4 (42.5-57.4)	56.9 (45.9-67.6)	62.4 (44-78)
Dominican Republic	80.7 (70.9-87.1)	85 (81.5-88.3)	88.3 (83.4-92.1)	90.2 (83-95.1)	69.4 (59-78.8)	71.5 (66.1-76.5)	73.2 (64.1-80.4)	74.4 (59.6-84.7)
Egypt	21.4 (14.4-29.4)	22.2 (18.3-25.9)	23.4 (17.9-29.7)	24.6 (15.3-35.2)	18.3 (14.4-22.6)	21.9 (19.5-24.5)	26.3 (22.5-30.5)	30.1 (23.4-37.6)
Ethiopia	1.7 (0.4-4.9)	5 (2.4-9.2)	15.4 (10.2-22)	33.8 (18.7-53.3)	1 (0.3-2.4)	3.5 (1.9-5.8)	12.5 (9.2-16.8)	30.8 (17.7-45.2)
Gabon	69.8 (41.9-88)	74.8 (62.7-84.6)	78.7 (69-86.1)	80.8 (62.7-92.2)	72.1 (44.7-90)	69.9 (55.6-81.3)	66.6 (54.1-76.7)	63.3 (41.6-82.1)
Ghana	27.3 (14.9-40.2)	39.7 (31.5-47.1)	53.9 (43.4-63.5)	64.6 (47.9-78.4)	23.3 (14.7-33.7)	37.7 (30.9-44.8)	55 (45.7-64.4)	68 (54.1-79.7)
Guatemala	25.4 (16.8-35.1)	40 (33.3-46.3)	56.7 (47.8-64.1)	69.1 (55.8-78.3)	23 (15.6-31.6)	35.6 (29.8-41.7)	50.8 (41.5-59.4)	62.8 (47.8-74.1)
Guinea	36.2 (19.2-58.1)	36.3 (27.4-46.2)	36.7 (28.3-45.4)	37.3 (22.7-54.4)	23.2 (10-43.7)	27.8 (19.8-38.6)	33.9 (25.2-43.3)	39.4 (23.4-58.6)
Haiti	9.2 (4.5-16.4)	15.8 (11-21.2)	26.5 (20.1-33.8)	38 (25.1-51.8)	7.5 (4.3-12.7)	13.4 (10.2-17.5)	23.3 (18.6-28.4)	34.3 (24.5-44.2)
Honduras	34.5 (3.5-87.2)	56.8 (27.3-83.4)	80.2 (72.2-87.2)	89.6 (72.7-97.5)	20.5 (0.9-75.1)	42.6 (14-75.1)	74.5 (64.9-82.1)	88.6 (68.4-97.1)
India	9 (4.6-16.1)	19.7 (13.9-26.5)	38.6 (27.7-49.2)	56.9 (39.4-72.9)	9.8 (5-16.3)	19.2 (14-25.9)	35 (25.3-45.3)	50.6 (32.7-68)
Indonesia	4.8 (3.1-7.2)	8.9 (7-11.3)	16.2 (12.5-20.6)	25 (17.1-34.7)	6.8 (4.4-9.8)	11.1 (8.7-13.8)	18 (13.9-23)	25.7 (17.1-36)
Jordan	61.7 (54.1-68)	59.6 (55-63.5)	57.5 (53.3-61.5)	55.7 (49.7-62.4)	58.6 (52-64)	61.5 (57.6-64.6)	64.3 (60.5-67.6)	66.5 (61.1-71.4)
Kazakhstan	100 (100-100)	100 (100-100)	100 (99.8-100)	99.8 (99.1-100)	98.1 (93.6-99.6)	98.2 (96.6-99.2)	96.3 (79.7-99.9)	92.7 (40.2-100)
Kenya	29.2 (19.6-42.5)	37.7 (31.2-45)	47.2 (37.9-56.5)	54.9 (39.3-69.4)	26.5 (16.2-39.5)	31.4 (24.6-38.2)	37.1 (28.1-46)	42 (27-57.1)
Kyrgyz Republic	100 (100-100)	100 (100-100)	100 (100-100)	100 (100-100)	91 (81.8-96.4)	96.9 (95.1-98.1)	98.9 (98.2-99.4)	99.5 (99-99.8)
Lesotho	18.8 (2.6-54)	40.4 (20.7-61.9)	70.2 (60.9-78.3)	85.9 (71-94.3)	20.7 (4.1-55.1)	38.4 (21.4-60.3)	63 (53.9-71.1)	78.7 (59-90.3)
Liberia	2.9 (0.4-10.6)	11.9 (4.3-24.3)	41.2 (33.5-49.4)	73.1 (62.7-82.1)	5.4 (0.8-20.7)	14.8 (5.7-30.6)	38.2 (31-45.4)	64.2 (50-75.9)
Madagascar	37 (25.5-50.5)	31.2 (25-37.8)	26.2 (17.1-35.7)	22.9 (11.3-38.7)	41.6 (29.1-55.8)	34.9 (28.8-41.2)	29 (20.1-39.6)	25.1 (12.5-43.3)
Malawi	28.8 (16.7-42.4)	48.5 (39.7-57.2)	69 (60.2-77)	81.3 (71-89.6)	28.1 (16.6-43.4)	44.8 (36-53.9)	63.1 (53.5-72.5)	75.3 (60.1-86)
Maldives	90.6 (55.8-99.6)	82.3 (56.5-96)	64.3 (52.2-75)	43.4 (26.6-60.6)	93.2 (64.3-99.7)	90.8 (73-98)	85.4 (78.7-90.5)	77 (63-87.7)
Mali	20.2 (9.5-38.4)	36.2 (27-46.2)	57 (44.4-68.4)	71.8 (52.6-86.8)	17.6 (7.4-31.8)	30.2 (22-39)	47.7 (36-60.4)	62 (41.4-79.8)
Morocco	31.7 (19.4-48)	57.3 (46.7-67.5)	79 (60.4-91.3)	88.7 (67.8-97.6)	22.2 (12.3-35.6)	44.8 (34.7-56.4)	69.4 (47.2-85.7)	82.7 (53.9-95.7)
Mozambique	37.3 (24.1-54.7)	51.5 (43.2-60.7)	65.7 (58.8-72.1)	75.3 (64.9-83.8)	33.4 (18.1-49.5)	45.6 (35.1-55.5)	58.6 (50.7-65.8)	68.2 (54.9-78.9)
Namibia	76.1 (65.3-84.5)	79.9 (74.5-84.6)	83 (76.6-87.6)	84.9 (75.9-91.3)	61.2 (50.5-70.1)	71.6 (66.6-75.8)	80.1 (74.4-84.5)	85.3 (77.7-90.3)

Nepal	2.2 (1-3.8)	9.8 (7-13.3)	35.6 (28.6-43.6)	66.7 (54.1-78.4)	2.3 (1.1-4.2)	8.2 (5.7-11.5)	26.1 (19.7-32.5)	51.6 (37.7-64.7)
Nicaragua	66.5 (13.1-96.6)	68.5 (58-77)	63.5 (10-97.4)	59.8 (1.3-99.7)	61.5 (12.4-96.5)	61.4 (50.7-71.8)	56.7 (6.1-95.3)	54 (0.7-99.3)
Niger	15 (8.5-24.5)	21 (16.1-26.6)	29.2 (21.1-39.3)	36.9 (22.4-55)	13.6 (8-21.1)	19 (14.2-23.9)	26.5 (18-35.8)	33.8 (19.2-51.1)
Nigeria	23.9 (15.4-34.2)	20.8 (16.2-26.4)	18.1 (13.6-24)	16.4 (9.7-24.7)	28.6 (17.9-40.6)	24.8 (18.6-30.5)	21.7 (15.7-28.3)	19.5 (11.3-29.5)
Pakistan	8.5 (4.1-15)	12.6 (8.4-17.9)	18.5 (13.3-24.4)	24.8 (16.2-35.6)	6.8 (3.8-10.6)	10.2 (7.3-13.6)	15.3 (11.9-19.3)	20.9 (14.3-28.5)
Peru	29.3 (21.8-38)	54.7 (50-59.4)	77.9 (74.5-81.4)	89.2 (85.1-92.3)	32.7 (27.9-37.3)	54.5 (52-57)	74.7 (72.8-76.5)	85.9 (83.7-88.1)
Philippines	11.1 (6-19.2)	23.8 (16.9-31.4)	44.5 (35.3-53.4)	62.8 (49.6-75.6)	12.9 (7.8-19.4)	23.5 (18.5-28.9)	39.1 (32.5-45.1)	53.7 (42.4-63.5)
Rwanda	14 (5.6-28.1)	48.3 (36-60)	85 (78.6-90.2)	95.8 (91.7-98.2)	11 (4.6-21.4)	33.5 (24-44)	68.2 (56.3-78.1)	86.8 (75-94.1)
Senegal	38.3 (29.7-47.4)	54.4 (49-59.6)	69.6 (66.2-72.6)	79.4 (75.5-83)	37.5 (33.2-42.2)	52.1 (49.1-55)	66.3 (64.4-68.2)	76 (73.5-78.3)
Sierra Leone	0.8 (0-5.1)	4.3 (0.4-17.3)	38 (28.4-48)	85.3 (64.8-96.4)	0.6 (0-4.4)	3.6 (0.5-13.8)	34 (24.9-42.4)	82.8 (58.3-94.8)
South Africa	77 (58.5-90.4)	90.5 (84.3-94.6)	96.4 (94.5-97.7)	98.4 (97-99.2)	69 (47.7-85.5)	77.8 (67.6-86.3)	84.5 (78-89.4)	88.3 (79.7-93.6)
Tajikistan	20.7 (0.2-89.7)	45.6 (6.6-90.3)	82.4 (67.3-92.3)	95.2 (91.3-97.6)	18.3 (0.2-86)	37.2 (5-86.5)	71.4 (53.4-85.7)	89.8 (81.7-94.8)
Tanzania	45.4 (34.8-55)	49.4 (42.9-55.3)	53.4 (46.9-59.7)	56.6 (46.3-66)	38.7 (29.6-48.6)	41.9 (36.4-48.2)	45.2 (39.3-51.6)	47.9 (38.2-56.8)
Timor-Leste	1.5 (0-9.5)	4.4 (0.5-17.2)	20.8 (12.4-32.9)	58 (37.2-75.5)	1.6 (0-9.5)	4.8 (0.6-18.3)	22.1 (13.7-33.1)	58.3 (37.6-75.3)
Togo	41.4 (21.8-63.7)	53 (40.7-65.6)	64.7 (54.2-73.8)	72.6 (56.6-85)	37.1 (18.7-58.5)	47.3 (34.4-60.2)	58.1 (46.6-67.8)	66 (48.3-80.6)
Turkey	65.6 (56.2-74.4)	69.4 (65-73.6)	72.9 (66.8-77.9)	75.3 (65.3-82.6)	61.7 (48.1-74.2)	63.9 (56-70.5)	65.8 (56.9-73.7)	67.1 (53.4-78.5)
Uganda	17.6 (11.1-25.1)	32.9 (27.4-39.3)	53.3 (47-59.1)	69.1 (60.1-77.3)	11.6 (6.7-18.4)	23.6 (18.4-29.3)	42.5 (35.9-49.3)	59.7 (48.5-70)
Vietnam	20.9 (4.6-55.2)	60.2 (49.2-70.6)	88.7 (62.5-98.4)	95 (68.6-99.9)	39.1 (11.2-76.6)	69.7 (60.1-77.9)	87.4 (59.5-98.1)	92.2 (55.4-99.8)
Yemen	13.9 (7.4-23.4)	18.1 (12.3-25.1)	23.6 (15.9-34.1)	29 (15.8-45.2)	11.1 (6-19)	14.2 (9.6-20.2)	18.4 (11.8-27.4)	22.6 (11.8-38.7)
Zambia	32.3 (21.9-44.6)	48.3 (40.6-55.8)	64.7 (55.5-73.6)	75.5 (62.2-86.4)	29.6 (19.2-44.4)	40 (32.2-47.8)	51.7 (40.2-62.8)	60.7 (42.4-76.9)
Zimbabwe	55.6 (40-70.8)	58.7 (50.4-67)	61.6 (53.9-69.6)	63.7 (50.7-76.4)	54.8 (40-68.4)	58.1 (50.2-65.4)	61.3 (53.5-68.5)	63.7 (51.5-74.5)

Table S23: Trends in the utilization (%, 95% credible intervals) of delivery in private facilities in low- and middle-income countries by age

	Adolescent, 15-19 years of age				Adult, 20-49 years of age			
**Country**	**1990**	**2000**	**2010**	**2018**	**1990**	**2000**	**2010**	**2018**
Afghanistan	11.4 (0.1-74.5)	8.3 (0.7-39)	6.7 (3.6-12.2)	8.1 (2.9-18.6)	13.1 (0.1-66.7)	8.2 (0.5-28.4)	5.5 (2.9-8.7)	5.6 (2.2-13.5)
Albania	N/A	N/A	N/A	N/A	0 (0-0)	0 (0-0)	0.3 (0.2-0.6)	4.6 (2.5-8)
Angola	0.2 (0-0.8)	0.2 (0.1-0.6)	0.3 (0.2-0.5)	0.6 (0.2-1.1)	2.3 (0.2-10.4)	1.9 (0.6-4.6)	1.8 (1.2-2.8)	2 (0.9-3.9)
Armenia	0.5 (0-2.6)	1.2 (0.3-3)	3.2 (2-4.7)	8.2 (3.3-16.4)	0 (0-0)	0.1 (0.1-0.3)	2 (1.1-3.2)	14.9 (6-30.5)
Bangladesh	0.3 (0.2-0.5)	1.8 (1.4-2.3)	10.1 (7.5-13.1)	32.6 (22-44.4)	0.8 (0.5-1.2)	3.3 (2.6-4)	12.8 (10.2-15.9)	32.3 (23.5-42.9)
Benin	7.8 (4.4-12.9)	8.7 (6.2-11.4)	9.8 (7.7-12.2)	10.9 (7.1-15.2)	10 (6-14.6)	11.5 (8.7-14.4)	13.4 (11-15.9)	15.2 (11.2-20.4)
Bolivia	11.9 (5.7-21.3)	8.5 (6.2-11.3)	6.5 (3.3-11.1)	5.5 (1.5-12.8)	14.8 (7-27.1)	11.5 (8.5-14.9)	9.5 (4.8-16.6)	8.5 (2.4-20.1)
Brazil	1.8 (0.8-3.5)	6 (1.4-15.5)	20.8 (0.6-75.1)	37.1 (0.4-96.6)	5.5 (2.4-9.9)	29.1 (10.9-53.9)	68.6 (15.7-97.6)	84.5 (19.4-99.9)
Burkina Faso	0.4 (0.2-0.7)	0.6 (0.4-0.9)	1.1 (0.6-2)	1.8 (0.6-4.6)	0.6 (0.4-1)	0.8 (0.6-1)	1 (0.6-1.4)	1.2 (0.5-2.2)
Burundi	12.5 (0.4-60.4)	8 (1.1-27.6)	5.5 (2.9-9.4)	4.8 (2.4-8.8)	22.2 (1.1-77.3)	11.1 (1.7-35.3)	5 (2.7-8.4)	3 (1.4-5.9)
Cambodia	1.4 (0.4-3.6)	3.8 (2.1-6.3)	10.7 (7.4-14.7)	23.1 (12-36.9)	0.4 (0.2-0.8)	2 (1.3-2.8)	9.6 (7.1-12.3)	29.1 (18.2-42.1)
Cameroon	11.1 (7.8-15)	15.1 (12.5-17.6)	20.3 (15.7-25.8)	25.4 (17-36.4)	12.3 (9.3-15.4)	16.7 (14.3-18.9)	22.3 (17.4-26.9)	27.8 (19.7-36)
Chad	1.5 (0.4-3.7)	1 (0.6-1.7)	0.8 (0.4-1.3)	0.7 (0.2-1.4)	1 (0.3-2.2)	0.9 (0.5-1.4)	0.9 (0.5-1.5)	1 (0.3-2.1)
Colombia	7.2 (3.5-13.2)	9.7 (6.3-14.1)	13.6 (7.3-22)	17.9 (6.9-33.6)	17.9 (6.8-36.7)	10.4 (5.9-16.6)	6.2 (3-12.1)	4.4 (1.3-11.8)
Comoros	N/A	N/A	N/A	N/A	0 (0-0.1)	0.2 (0.1-0.3)	1.7 (1-2.7)	8.7 (3.6-16.9)
Republic of the Congo	1.1 (0.1-4.7)	3.5 (1.1-8.4)	14.6 (9.7-20.9)	39.7 (16.3-66.7)	4.1 (0.3-18.9)	6.4 (2-15.1)	12.4 (8.1-17.9)	22.7 (7.5-46)
Democratic Republic of the Congo	63.1 (10.9-95.8)	42.1 (13.5-71.4)	20.6 (14.4-28.8)	11 (4.1-23.5)	38.6 (3.3-87.9)	28.3 (7.7-57.6)	19.4 (13.5-26.6)	15.5 (5.4-33.1)
Cote d'Ivoire	0.2 (0.1-0.4)	0.6 (0.3-0.9)	2 (1.1-3.6)	6 (1.9-14.7)	0.5 (0.2-1)	1.4 (0.9-2)	3.8 (2.2-6.2)	8.8 (3.6-18.9)
Dominican Republic	15 (9.4-21.6)	12.8 (10-16)	11.1 (7.8-15.2)	10.1 (5.5-16.3)	25.4 (17.3-34.2)	25.6 (21.2-31.1)	26.1 (19.3-33.9)	26.6 (16.3-39.8)
Egypt	5.8 (3.4-9.4)	23.1 (17.9-28.2)	59.7 (49.7-68.5)	83.8 (73.8-90.9)	10.1 (7.5-12.9)	26.4 (23.4-29.8)	53.7 (48.3-58.9)	74.6 (68.1-80.9)
Ethiopia	0 (0-0.1)	0 (0-0.1)	0.1 (0.1-0.3)	0.4 (0.1-0.9)	0.1 (0-0.2)	0.2 (0.1-0.3)	0.7 (0.5-1)	2 (1-3.7)
Gabon	17.5 (5-37.1)	15.3 (8.5-24)	14 (9.1-20.4)	13.7 (5.9-27.7)	11.2 (3.3-26)	16.4 (9.6-25.6)	24.9 (16.9-34.7)	34 (16.2-54.7)
Ghana	11.2 (5.7-19.3)	10 (7.1-13.7)	9.2 (5.7-13.9)	8.8 (3.9-17)	11.8 (8.3-15.9)	10.2 (8.6-12.3)	9 (7.1-11.2)	8.2 (5.7-11.8)
Guatemala	5.3 (2.7-9.4)	5.6 (3.8-7.9)	6.1 (3.7-9.2)	6.6 (3-12.5)	6.1 (3.6-9.7)	7 (5.2-9.5)	8.4 (5.5-11.8)	9.7 (5.2-16.4)
Guinea	0.5 (0.1-1.2)	1.1 (0.6-1.8)	3.1 (1.9-4.9)	7.1 (2.6-14.8)	0.4 (0.1-0.9)	1.1 (0.7-1.9)	3.8 (2.4-5.8)	10.2 (4.2-20.4)
Haiti	27.4 (13.5-44.8)	14.2 (9.2-20.6)	6.9 (4.5-10)	3.9 (1.7-7.2)	10.7 (5.1-19.4)	9.1 (6.3-13)	8.1 (5.7-11.1)	7.5 (4-12)
Honduras	1.6 (0-7.8)	1.9 (0.4-5.3)	3.6 (2.2-5.3)	7.9 (1.8-22.8)	12.7 (0.5-58.3)	7.7 (1.6-21.9)	5.3 (3.5-7.9)	5.3 (1.1-15.4)
India	8.5 (5.6-11.8)	13 (10.3-16)	19.5 (15.5-24.6)	26.4 (18.7-35.8)	11.6 (7.3-17.2)	16.7 (13.3-20.7)	23.7 (17.9-29.9)	30.7 (20.5-41.9)
Indonesia	4.5 (2.7-6.8)	12.3 (9.4-15.6)	30.1 (22.8-37.4)	51.3 (37.3-63.7)	9.8 (6.5-14.2)	21.3 (17.1-25.5)	40.5 (33.1-47.7)	58.6 (46.3-69.4)
Jordan	29.8 (22.8-37.2)	34.4 (29.7-39.1)	39.4 (33.8-44.4)	43.7 (34.9-51.4)	28.3 (21.5-36.2)	30.9 (26.6-35.7)	33.8 (28.6-39.3)	36.2 (28-45.1)
Kenya	9.7 (6.1-13.9)	9.9 (7.8-12.2)	10.2 (7.5-13.4)	10.6 (6.5-16.4)	9.7 (5.9-15)	11.8 (9.1-14.6)	14.4 (11-18.5)	17.1 (10.8-25.5)
Lesotho	4.7 (0.9-14.9)	3.5 (1.6-6.8)	2.9 (2-3.9)	2.7 (1.3-5.1)	0.8 (0.1-3.1)	1.4 (0.5-3.1)	3 (2-4.3)	6.4 (2.4-13.4)
Liberia	9.4 (1.6-29.7)	10.1 (4-20.7)	11.5 (8.5-15)	13.5 (8.5-20.4)	6.7 (0.7-24.9)	8.4 (2.6-18.8)	11.7 (8.2-15.9)	16.3 (9.4-26)
Madagascar	0.4 (0.2-0.7)	0.9 (0.6-1.2)	2.3 (1.2-4)	4.9 (1.7-11.3)	1.2 (0.7-2)	2 (1.5-2.6)	3.3 (2-5.2)	5.2 (2.1-10.1)
Malawi	18.7 (10.9-29.4)	17.3 (12.8-22.9)	16.4 (11.4-21.8)	15.8 (8.8-24.2)	17.1 (9.2-29.3)	16.6 (11.7-22.9)	16.6 (11.7-22.7)	16.8 (9.4-27.1)
Maldives	8.9 (0.5-40.7)	17.1 (4-42.6)	35.3 (23.8-48)	56.5 (39.7-71.8)	4.3 (0.2-23.4)	6.7 (1.6-19.8)	12.8 (7.9-19.2)	23.3 (13.7-37.4)
Mali	0.6 (0.2-1.2)	1 (0.6-1.4)	1.8 (1.1-2.8)	3 (1.2-6.1)	0.3 (0.1-0.7)	0.9 (0.6-1.4)	2.8 (1.6-4.4)	6.8 (2.8-13.5)
Morocco	1.9 (0.9-3.6)	1.1 (0.7-1.7)	0.8 (0.3-1.6)	0.6 (0.1-1.8)	3.8 (1.8-6.8)	6.3 (4-9.3)	11.1 (4.4-21.8)	17.2 (4.3-43.1)
Mozambique	0.1 (0-0.4)	0.2 (0.1-0.3)	0.2 (0.1-0.5)	0.4 (0.1-1.1)	0 (0-0.1)	0.1 (0-0.1)	0.2 (0.1-0.4)	0.5 (0.2-1)
Namibia	0.6 (0.2-1.4)	1.2 (0.7-1.9)	2.4 (1.2-4.8)	4.5 (1.5-10.7)	2.2 (1.1-3.8)	3.4 (2.4-4.7)	5.5 (3.6-8)	8 (4-14.7)
Nepal	0.4 (0.2-0.9)	1.5 (0.9-2.3)	5.3 (3.5-7.8)	14.5 (7.4-24.8)	0.5 (0.2-0.9)	1.8 (1.2-2.5)	6.3 (4.7-8.6)	16.3 (10.2-25.4)
Nicaragua	1.8 (0.1-9.4)	2.4 (1.5-3.6)	12.4 (0.5-56.3)	28 (0.2-96.8)	1.5 (0-7.7)	6.4 (4.1-9.5)	43 (3.6-95.3)	70.2 (2.7-99.9)

Niger	0.5 (0.2-0.8)	0.4 (0.3-0.6)	0.4 (0.2-0.6)	0.4 (0.2-0.8)	0.2 (0.1-0.5)	0.3 (0.2-0.5)	0.4 (0.3-0.7)	0.6 (0.2-1.3)
Nigeria	0.9 (0.4-1.8)	2.5 (1.5-3.8)	6.9 (3.9-11.2)	15.2 (6.6-29.5)	2.6 (1.1-4.9)	6.9 (4.3-10)	17.3 (11-24.9)	32.8 (17.6-50.8)
Pakistan	5 (2.5-8.9)	12.1 (8-16.8)	27.1 (20.2-35)	45.1 (32.9-57.9)	6.4 (4-9.5)	14.6 (11.6-18.5)	30.2 (24.9-35.8)	47.7 (38.3-56.5)
Peru	4.2 (2.4-6.4)	4.3 (3.3-5.4)	4.5 (3.4-5.7)	4.8 (2.9-7.2)	8.4 (5.7-11.9)	8.3 (6.8-10)	8.3 (6.8-9.9)	8.3 (5.8-11.3)
Philippines	6.2 (4.1-9)	9.6 (7.8-11.7)	14.7 (12-17.7)	20.3 (14.8-27)	10.6 (7.2-15.1)	14.5 (11.5-17.5)	19.7 (16.3-23.3)	24.8 (18.9-31.4)
Rwanda	0.6 (0-2.9)	0.9 (0.3-1.9)	2.5 (0.8-5.8)	8.1 (0.6-32.2)	2.3 (0.9-4.8)	1.5 (1-2.1)	1 (0.8-1.3)	0.8 (0.4-1.2)
Senegal	1.6 (0.9-2.7)	1.8 (1.3-2.5)	2.1 (1.7-2.6)	2.5 (1.7-3.4)	3.9 (3.1-5)	4.1 (3.5-4.6)	4.3 (3.9-4.6)	4.5 (3.9-5)
Sierra Leone	6.8 (0.1-42.9)	2.8 (0.3-10.8)	1.5 (1-2.4)	1.5 (0.3-4.7)	15.4 (0.2-64.8)	6.3 (0.8-19.4)	2.6 (1.7-3.8)	1.9 (0.5-5.3)
South Africa	5.3 (2-12.4)	2.3 (1.3-3.8)	1 (0.6-1.6)	0.5 (0.2-1)	9.2 (3.4-19.6)	8.8 (4.9-14.2)	8.6 (5.5-12.8)	8.8 (4.8-15.4)
Tanzania	4.9 (2.9-7.7)	6.5 (4.9-8.4)	8.6 (6.4-11.1)	10.9 (6.7-16.1)	4.8 (3-7.2)	6.7 (5.1-8.3)	9.3 (7.2-12.1)	12.1 (7.8-18.1)
Togo	2.1 (0.7-5)	3.3 (1.8-5.5)	5.4 (3.2-8.4)	8.3 (3.7-15.2)	2.7 (1-6.4)	5.1 (2.9-8)	9.9 (6-14.9)	16.6 (7.8-28.9)
Turkey	5.5 (3-9.3)	11.1 (8.5-14.4)	21.7 (15.6-29.5)	34.5 (21.4-50.4)	3.2 (2-5)	10.3 (8.1-12.7)	28.8 (23-35.9)	52.3 (40.1-65.9)
Uganda	18 (10.2-27.7)	15.2 (11.4-19.8)	12.9 (10-16.5)	11.5 (7.2-17.1)	12.8 (7.8-18.8)	13.6 (10.7-16.9)	14.7 (12-17.8)	15.7 (11.2-22.4)
Vietnam	N/A	N/A	N/A	N/A	0.7 (0.1-2.8)	2.8 (1.8-3.9)	15.5 (2.1-48.2)	39.6 (2-93.2)
Yemen	1.2 (0.6-2.1)	3.5 (2.2-5.2)	9.9 (6-14.9)	21.3 (10.7-34.5)	1 (0.5-1.7)	2.9 (1.9-4.3)	8.5 (5.2-12.8)	18.8 (9.7-30.8)
Zambia	10.2 (7-14.8)	7 (5.6-8.7)	4.8 (3.6-6.3)	3.5 (2.2-5.5)	12.9 (8.8-17.9)	8.6 (6.7-10.4)	5.7 (4.1-7.7)	4.1 (2.3-6.5)
Zimbabwe	15.7 (9.3-25)	13.3 (10.1-17.6)	11.5 (8.6-14.8)	10.3 (6.2-15.3)	14.3 (9-20.5)	12.8 (10.1-15.8)	11.5 (9.1-14.4)	10.7 (7.1-15.4)

Note: N/A denotes not application and refers that the indicator has no sufficient data to examine trends

Table S24: Change rates in the utilisation of delivery services in public facilities in low- and middle-income countries by wealth quintiles

	Poorest				Richest			
**Country**	**1990-1999**	**2000-2009**	**2010-2018**	**1990-2018**	**1990-1999**	**2000-2009**	**2010-2018**	**1990-2018**
Afghanistan	5.3	10.4	12.5	9.4	6.9	8.8	5.4	7.2
Albania	14.0	4.5	0.5	6.5	0.0	-0.1	-1.9	-0.6
Angola	-2.0	-1.6	-0.6	-1.4	-0.3	-0.7	-1.4	-0.8
Armenia	1.7	0.8	0.4	1.0	0.0	-0.2	-1.3	-0.5
Bangladesh	16.4	16.4	15.1	16.1	2.8	3.0	2.9	2.9
Benin	3.4	2.7	2.0	2.7	-0.1	-0.2	-0.2	-0.1
Bolivia	10.0	8.7	5.6	8.2	1.8	1.4	1.0	1.4
Burkina Faso	3.1	3.0	2.6	2.9	0.4	0.3	0.2	0.3
Burundi	13.9	13.6	6.3	11.6	10.2	8.1	3.0	7.3
Cambodia	36.0	33.2	12.3	27.9	4.7	3.8	2.6	3.8
Cameroon	-2.7	-2.8	-2.8	-2.8	-0.9	-1.1	-1.3	-1.1
Chad	13.2	14.1	13.7	13.7	3.9	3.3	2.5	3.3
Colombia	3.6	2.4	1.4	2.5	1.4	1.0	0.7	1.1
Comoros	8.5	6.6	3.9	6.4	1.3	0.8	0.5	0.9
Republic of the Congo	1.3	1.2	0.8	1.1	0.3	0.1	-0.2	0.0
Democratic Republic of the Congo	2.3	2.4	1.9	2.2	1.6	2.0	1.9	1.8
Cote d'Ivoire	4.6	4.4	3.6	4.2	0.0	0.0	-0.1	0.0
Dominican Republic	0.4	0.2	0.2	0.3	-0.9	-0.9	-0.9	-0.9
Egypt	5.7	5.4	4.7	5.3	-1.5	-1.5	-1.6	-1.5
Ethiopia	17.7	18.7	18.3	18.2	11.1	9.2	5.4	8.7
Gabon	2.4	1.8	1.2	1.8	-1.3	-1.9	-2.3	-1.8
Ghana	5.3	5.0	4.2	4.9	1.5	1.1	0.8	1.2
Guatemala	9.0	8.3	6.4	8.0	0.1	0.1	0.1	0.1
Guinea	4.3	4.6	4.5	4.5	-0.2	-0.3	-0.4	-0.3
Haiti	8.7	8.7	8.4	8.6	4.0	3.3	2.6	3.3
Honduras	9.6	9.9	5.1	8.4	2.2	1.6	0.7	1.6
India	11.9	10.9	8.0	10.4	0.8	0.9	0.9	0.9
Indonesia	13.1	12.8	10.9	12.3	0.4	0.6	0.8	0.6
Jordan	0.4	0.4	0.3	0.4	-0.9	-0.9	-0.9	-0.9
Kazakhstan	-0.3	-1.4	-2.6	-1.4	0.0	0.0	0.0	0.0
Kenya	2.7	2.9	2.9	2.8	0.3	0.3	0.3	0.3
Kyrgyz Republic	4.4	0.5	0.0	1.6	0.0	0.0	0.0	0.0
Lesotho	7.9	7.8	5.0	7.0	1.3	0.9	0.5	0.9
Liberia	19.2	18.9	11.4	16.8	0.0	0.1	0.1	0.1
Madagascar	-2.7	-2.6	-2.2	-2.5	3.0	2.6	2.0	2.6
Malawi	6.1	4.6	2.8	4.6	2.2	1.6	1.1	1.7
Maldives	0.5	0.3	0.1	0.3	-0.8	-2.5	-5.9	-3.0
Mali	8.7	8.4	6.4	7.9	2.7	1.7	0.9	1.8
Morocco	16.6	12.1	4.7	11.4	1.2	1.0	0.7	1.0
Mozambique	6.5	5.8	4.4	5.7	1.3	0.7	0.4	0.8
Namibia	2.3	1.8	1.3	1.8	-0.7	-1.0	-1.4	-1.0
Nepal	18.3	18.0	14.8	17.2	7.0	5.5	3.6	5.4
Nicaragua	-4.6	-3.2	-0.8	-3.0	-1.6	-5.3	-4.7	-3.8
Niger	8.3	9.1	8.7	8.7	0.8	0.7	0.5	0.7
Nigeria	-4.2	-3.5	-2.3	-3.4	1.9	2.0	1.9	1.9
Pakistan	8.8	8.8	8.4	8.6	-0.5	-0.5	-0.4	-0.5
Peru	11.8	9.1	5.0	8.8	0.9	0.8	0.6	0.8
Philippines	10.5	9.6	7.4	9.3	1.2	1.2	1.2	1.2
Rwanda	11.9	8.8	4.1	8.4	7.1	3.8	1.5	4.2
Senegal	5.0	4.3	3.5	4.3	1.2	0.9	0.6	0.9
Sierra Leone	20.5	25.9	13.2	20.4	18.0	22.8	9.3	17.2
South Africa	3.3	1.8	0.8	2.0	0.4	0.4	0.3	0.4
Tajikistan	8.2	10.2	5.9	8.3	4.2	2.8	0.9	2.7
Tanzania	2.5	2.4	2.2	2.3	0.0	0.0	0.0	0.0
Timor-Leste	13.7	19.2	20.1	17.7	12.8	12.8	5.3	10.6
Togo	4.4	4.0	3.3	3.9	-0.1	-0.2	-0.3	-0.2
Turkey	7.9	4.6	2.0	4.9	-2.2	-4.6	-7.0	-4.5
Uganda	9.9	8.7	6.0	8.3	2.8	2.3	1.8	2.3
Vietnam	5.0	4.2	2.0	3.8	1.8	0.4	0.1	0.8
Yemen	-0.3	-1.5	-40.0	-12.2	0.0	-0.2	17.3	4.8
Zambia	8.9	8.0	5.8	7.7	3.4	1.9	0.9	2.1
Zimbabwe	1.3	1.3	1.2	1.3	-0.6	-0.8	-1.0	-0.8

Supplement to: Hasan MM, Magalhaes RJS, Fatima Y, Ahmed S, Mamun AA. Levels, trends and inequalities in using institutional delivery services in low- and middle-income countries: a stratified analysis by facility type. *Glob Health Sci Pract*. 2021;9(1). https://doi.org/10.9745/GHSP-D-20-00533

Table S25: Change rates in the utilization of delivery services in private facilities in low- and middle-income countries by wealth quintiles

	Poorest				Richest			
**Country**	**1990-1999**	**2000-2009**	**2010-2018**	**1990-2018**	**1990-1999**	**2000-2009**	**2010-2018**	**1990-2018**
Afghanistan	-2.4	3.8	9.4	3.4	-2.8	-3.6	-1.8	-2.8
Albania	N/A	N/A	N/A	N/A	31.8	34.5	34.4	33.6
Angola	6.0	8.4	10.2	8.1	-0.7	0.8	2.4	0.8
Armenia	17.5	21.4	23.1	20.6	9.8	12.4	14.0	12.0
Bangladesh	23.1	23.9	23.1	23.4	11.1	9.5	6.5	9.1
Benin	0.2	0.6	1.0	0.6	0.7	0.7	0.7	0.7
Bolivia	2.5	4.0	5.3	3.9	-1.6	-1.6	-1.5	-1.6
Burkina Faso	N/A	N/A	N/A	N/A	2.7	3.0	3.3	3.0
Burundi	-10.4	-16.1	-15.3	-14.0	-4.0	-4.2	-2.5	-3.7
Cambodia	22.5	24.5	25.0	24.0	9.7	8.9	6.7	8.5
Cameroon	-0.6	0.0	0.6	0.0	3.0	2.7	2.4	2.7
Chad	-0.7	0.1	0.7	0.0	2.5	3.3	3.9	3.2
Colombia	-3.2	-2.4	-1.6	-2.4	-3.8	-4.2	-3.9	-4.0
Republic of the Congo	6.4	9.7	10.9	9.0	-1.5	0.1	2.1	0.1
Democratic Republic of the Congo	-4.1	-2.9	-0.6	-2.6	-0.7	-1.1	-1.4	-1.1
Cote d'Ivoire	8.4	10.7	12.7	10.5	8.5	8.8	8.2	8.5
Dominican Republic	0.9	1.6	2.3	1.6	1.2	1.1	0.9	1.1
Egypt	14.2	12.2	7.5	11.5	3.7	2.6	1.6	2.6
Ethiopia	14.2	15.9	17.4	15.8	13.4	13.9	13.8	13.7
Gabon	-0.6	0.3	1.1	0.2	3.0	2.9	2.3	2.7
Ghana	-6.7	-6.6	-6.3	-6.5	-0.8	-0.7	-0.6	-0.7
Guatemala	-1.1	-0.5	0.1	-0.5	0.5	0.6	0.6	0.6
Guinea	N/A	N/A	N/A	N/A	10.7	10.6	9.0	10.2
Haiti	0.9	1.8	2.5	1.7	-1.8	-1.9	-1.9	-1.9
Honduras	5.1	10.6	14.3	9.9	-3.6	-4.6	-3.1	-3.8
India	7.8	8.0	7.8	7.9	1.8	1.6	1.3	1.6
Indonesia	15.6	15.7	13.4	15.0	4.3	3.0	1.7	3.0
Jordan	5.1	4.9	4.5	4.8	1.0	0.8	0.7	0.8
Kenya	-2.6	-2.3	-1.9	-2.3	2.2	2.1	1.9	2.1
Lesotho	6.1	7.5	8.7	7.4	14.5	17.7	17.9	16.7
Liberia	-4.7	-3.8	-2.5	-3.7	1.7	2.1	2.3	2.0
Madagascar	-15.3	-14.1	-12.8	-14.1	6.6	7.0	6.6	6.8
Malawi	0.8	1.1	1.2	1.0	-0.9	-0.8	-0.6	-0.8
Maldives	5.8	9.0	10.8	8.5	7.5	8.3	6.6	7.5
Mali	11.6	13.1	14.1	12.9	8.2	8.2	7.8	8.1
Morocco	N/A	N/A	N/A	N/A	4.0	3.7	2.9	3.6
Mozambique	4.4	5.7	6.8	5.6	10.8	11.6	12.4	11.6
Namibia	-12.3	-10.5	-8.2	-10.4	7.0	6.5	5.5	6.4
Nepal	4.8	5.4	5.8	5.3	9.4	9.2	8.0	8.9
Nicaragua	-22.9	-4.9	12.6	-5.6	17.2	12.9	2.2	11.2
Niger	-3.3	1.1	4.8	0.7	7.0	7.9	8.5	7.8
Nigeria	-12.0	-13.6	-13.0	-12.9	-1.4	-1.8	-1.9	-1.7
Pakistan	15.7	15.2	12.5	14.6	5.4	4.0	2.7	4.1
Peru	0.0	0.3	0.6	0.3	-0.8	-0.7	-0.6	-0.7
Philippines	8.9	9.2	9.2	9.1	1.5	1.3	1.1	1.3
Rwanda	-2.3	0.4	2.8	0.2	-2.4	-2.0	-1.6	-2.0
Senegal	4.3	4.9	5.4	4.8	-0.5	-0.4	-0.3	-0.4
Sierra Leone	-16.8	-27.8	-23.5	-22.9	-4.7	-6.8	-4.3	-5.4
South Africa	-6.5	-6.1	-5.6	-6.1	-0.6	-0.6	-0.5	-0.6
Tanzania	3.5	3.7	3.9	3.7	6.7	6.5	6.0	6.4
Togo	-0.1	0.6	1.3	0.6	2.5	2.7	2.6	2.6
Turkey	9.0	9.2	8.8	9.0	9.8	7.1	3.8	7.0
Uganda	0.2	0.5	0.8	0.5	0.5	0.5	0.6	0.5
Vietnam	14.7	18.3	10.6	14.8	14.9	18.9	11.7	15.4
Zambia	-1.1	-0.9	-0.7	-0.9	-6.6	-8.1	-8.4	-7.7
Zimbabwe	-4.0	-4.2	-4.1	-4.1	4.1	4.0	3.8	4.0

Note: N/A denotes not application and refers that the indicator has no sufficient data to examine trends

Table S26: Change rates in the utilization of delivery services in public facilities in low- and middle-income countries by place of residence

Country	Rural				Urban			
	1990-1999	2000-2009	2010-2018	1990-2018	1990-1999	2000-2009	2010-2018	1990-2018
Afghanistan	4.9	8.3	8.6	7.3	3.0	3.9	3.0	3.3
Albania	0.2	0.1	0.0	0.1	0.0	-0.1	-0.7	-0.2
Angola	-2.4	-2.7	-2.2	-2.4	-0.6	-1.4	-2.6	-1.5
Armenia	0.9	0.5	0.2	0.5	-0.1	-0.1	-0.2	-0.1
Bangladesh	12.2	12.0	10.8	11.7	1.7	1.7	1.7	1.7
Benin	1.8	1.4	1.1	1.5	0.8	0.7	0.6	0.7
Bolivia	8.1	6.5	4.1	6.3	4.5	2.8	1.5	3.0
Brazil	2.9	0.9	0.3	1.4	-0.1	-0.5	-0.5	-0.3
Burkina Faso	4.4	3.7	2.6	3.6	0.1	0.1	0.0	0.1
Burundi	15.6	14.6	5.8	12.3	3.5	2.8	1.4	2.6
Cambodia	25.6	21.9	8.4	19.1	10.1	7.8	4.2	7.5
Cameroon	-0.8	-0.7	-0.7	-0.7	-1.1	-1.3	-1.4	-1.2
Chad	8.7	8.9	8.5	8.7	3.1	2.7	2.2	2.7
Colombia	2.4	1.6	1.0	1.7	1.4	0.9	0.5	1.0
Comoros	6.6	4.5	2.3	4.5	2.1	1.3	0.8	1.4
Republic of the Congo	1.7	1.5	0.8	1.4	-0.2	-0.5	-1.1	-0.6
Democratic Republic of the Congo	5.1	4.8	2.9	4.3	1.3	1.6	1.4	1.4
Cote d'Ivoire	2.6	2.3	2.0	2.3	0.1	0.1	0.0	0.1
Dominican Republic	0.1	0.1	0.1	0.1	0.6	0.5	0.4	0.5
Egypt	4.3	4.1	3.7	4.1	0.0	0.0	0.0	0.0
Ethiopia	15.9	16.7	15.5	16.1	8.8	6.9	3.8	6.6
Gabon	0.6	0.5	0.4	0.5	-0.4	-0.7	-0.9	-0.7
Ghana	5.8	4.9	3.6	4.8	1.8	1.4	1.0	1.4
Guatemala	6.8	5.7	4.1	5.6	1.8	1.5	1.1	1.5
Guinea	3.3	3.5	3.2	3.3	-0.3	-0.3	-0.4	-0.3
Haiti	8.1	7.9	7.1	7.7	4.1	3.6	3.0	3.6
Honduras	8.8	7.8	3.2	6.8	3.9	2.4	0.9	2.4
India	8.9	7.9	5.8	7.6	2.1	2.0	1.8	2.0
Indonesia	8.3	7.9	7.0	7.7	1.0	1.1	1.1	1.0
Jordan	0.7	0.6	0.4	0.6	0.6	0.6	0.5	0.6
Kazakhstan	0.0	-0.3	-0.7	-0.3	0.5	0.0	0.0	0.1
Kenya	1.8	1.8	1.7	1.8	0.0	0.0	0.0	0.0
Kyrgyz Republic	0.8	0.2	0.1	0.4	0.1	0.0	0.0	0.1
Lesotho	6.8	5.9	3.3	5.5	0.1	-0.1	-0.3	-0.1
Liberia	16.6	16.6	9.7	14.6	1.6	1.7	1.5	1.6
Madagascar	-2.1	-2.2	-2.2	-2.2	0.2	0.2	0.3	0.2
Malawi	5.4	4.0	2.5	4.0	1.4	1.0	0.7	1.1
Maldives	0.0	-0.2	-0.4	-0.2	-0.6	-1.4	-2.8	-1.6
Mali	8.8	7.7	5.2	7.3	2.5	1.6	1.0	1.7
Morocco	9.6	6.9	3.4	6.8	3.2	2.0	1.1	2.1
Mozambique	4.3	3.7	2.7	3.6	0.6	0.5	0.3	0.5
Namibia	2.1	1.5	1.0	1.6	0.2	0.1	0.1	0.1
Nepal	13.6	12.8	9.7	12.1	3.3	2.8	2.2	2.8
Nicaragua	1.2	1.5	0.7	1.2	-0.1	-1.7	-1.7	-1.2
Niger	7.3	7.2	6.3	7.0	0.7	0.6	0.4	0.6
Nigeria	-1.4	-1.4	-1.3	-1.4	-2.2	-2.7	-2.9	-2.6
Pakistan	6.8	6.7	6.2	6.6	1.4	1.5	1.5	1.4
Peru	12.0	8.4	3.9	8.3	2.6	1.6	0.9	1.7
Philippines	9.2	7.9	5.6	7.7	3.7	3.2	2.6	3.2
Rwanda	12.2	8.2	3.3	8.1	3.5	2.2	1.2	2.3
Senegal	5.1	3.8	2.6	3.9	1.2	0.9	0.6	0.9
Sierra Leone	17.6	24.4	12.3	18.6	16.2	20.0	8.4	15.3
South Africa	2.1	1.2	0.7	1.4	0.6	0.4	0.3	0.5
Tajikistan	7.5	7.5	3.4	6.3	6.1	4.4	1.3	4.0
Tanzania	1.1	1.1	1.0	1.1	-0.2	-0.2	-0.3	-0.2
Timor-Leste	14.1	18.2	16.4	16.4	13.7	15.5	6.8	12.3
Togo	3.4	2.9	2.2	2.8	-0.1	-0.1	-0.2	-0.1
Turkey	3.6	2.4	1.4	2.5	-0.6	-0.8	-0.9	-0.8
Uganda	7.9	6.7	4.7	6.5	1.9	1.6	1.3	1.6
Vietnam	6.5	2.9	0.8	3.5	2.1	0.5	0.1	0.9
Yemen	3.6	3.7	3.6	3.7	-0.3	-0.2	-0.1	-0.2
Zambia	6.2	5.3	4.0	5.2	2.4	1.5	0.9	1.6
Zimbabwe	1.1	1.0	0.9	1.0	-0.2	-0.3	-0.4	-0.3

Supplement to: Hasan MM, Magalhaes RJS, Fatima Y, Ahmed S, Mamun AA. Levels, trends and inequalities in using institutional delivery services in low- and middle-income countries: a stratified analysis by facility type. *Glob Health Sci Pract*. 2021;9(1). https://doi.org/10.9745/GHSP-D-20-00533

Table S27: Change rates in the utilization of delivery services in private facilities in low- and middle-income countries by place of residence

	Rural				Urban			
**Country**	**1990-1999**	**2000-2009**	**2010-2018**	**1990-2018**	**1990-1999**	**2000-2009**	**2010-2018**	**1990-2018**
Afghanistan	-6.4	-6.7	-2.2	-5.3	-3.0	-3.9	-1.3	-2.8
Albania	N/A	N/A	N/A	N/A	21.3	24.1	25.4	23.6
Angola	4.7	8.4	10.5	7.8	-2.5	-0.6	1.3	-0.7
Armenia	26.6	29.9	30.6	29.0	18.7	20.4	20.7	20.0
Bangladesh	18.5	18.1	15.4	17.4	6.7	6.4	5.5	6.2
Benin	1.7	1.9	2.1	1.9	0.3	0.4	0.4	0.4
Bolivia	-6.9	-6.2	-5.3	-6.2	-1.8	-1.3	-0.8	-1.3
Brazil	12.4	11.8	6.2	10.4	11.8	7.7	3.2	7.7
Burkina Faso	0.5	1.6	2.6	1.5	0.4	0.9	1.3	0.9
Burundi	-7.7	-9.7	-8.2	-8.6	-1.9	-1.2	0.7	-0.9
Cambodia	18.1	18.2	16.3	17.6	12.0	11.2	8.4	10.6
Cameroon	1.4	1.4	1.5	1.4	3.7	3.4	2.9	3.3
Chad	-4.8	-3.7	-2.7	-3.8	3.1	3.7	4.2	3.7
Colombia	-5.1	-4.3	-3.5	-4.3	-5.5	-5.5	-4.8	-5.3
Comoros	N/A	N/A	N/A	N/A	15.5	16.0	15.4	15.7
Republic of the Congo	4.9	8.2	9.8	7.6	2.9	4.8	5.6	4.4
Democratic Republic of the Congo	-5.9	-6.8	-5.0	-5.9	-0.6	-0.7	-0.4	-0.6
Cote d'Ivoire	7.4	8.3	9.0	8.2	9.8	9.9	9.4	9.7
Dominican Republic	2.1	2.2	2.2	2.2	-1.3	-1.2	-1.2	-1.2
Egypt	13.1	9.9	5.0	9.5	6.6	4.8	3.0	4.9
Ethiopia	8.6	9.3	9.8	9.2	12.0	12.5	12.3	12.3
Gabon	-1.0	-0.1	0.8	-0.1	2.8	3.2	3.0	3.0
Ghana	-2.8	-2.7	-2.5	-2.7	-1.9	-1.9	-1.8	-1.9
Guatemala	1.2	1.6	1.9	1.5	1.5	1.6	1.7	1.6
Guinea	6.9	8.5	9.8	8.3	12.2	12.3	10.8	11.8
Haiti	0.1	0.6	1.1	0.6	-3.0	-3.1	-3.1	-3.1
Honduras	0.2	4.0	7.2	3.7	-6.0	-6.7	-3.8	-5.6
India	5.0	4.9	4.5	4.8	1.7	1.6	1.5	1.6
Indonesia	10.3	9.3	7.2	9.0	4.9	3.7	2.5	3.8
Jordan	2.8	2.9	2.8	2.8	0.3	0.3	0.3	0.3
Kenya	0.0	0.2	0.3	0.2	1.9	1.9	1.8	1.9
Lesotho	4.2	5.6	6.6	5.4	5.7	9.3	11.7	8.8
Liberia	-1.4	-0.6	0.4	-0.6	-0.6	-0.1	0.7	0.0
Madagascar	6.2	6.9	7.3	6.8	4.8	5.4	5.5	5.2
Malawi	-0.1	0.1	0.3	0.1	-1.8	-1.7	-1.5	-1.7
Maldives	4.4	6.8	8.3	6.4	4.4	5.4	5.4	5.1
Mali	12.5	13.7	14.3	13.5	10.4	10.3	9.8	10.2
Morocco	4.7	5.7	6.6	5.6	1.6	2.2	2.4	2.1
Mozambique	3.1	3.9	4.7	3.9	9.3	10.1	10.8	10.0
Namibia	1.1	2.1	2.9	2.0	3.9	4.1	4.2	4.1
Nepal	13.1	13.2	12.5	12.9	6.4	6.7	6.6	6.6
Nicaragua	-3.1	13.1	11.2	7.2	16.6	18.6	4.2	13.7
Niger	-5.4	-4.4	-3.5	-4.5	11.0	11.4	11.4	11.3
Nigeria	8.0	8.3	8.0	8.1	9.2	8.4	6.2	8.0
Pakistan	12.6	11.7	8.9	11.2	5.4	4.5	3.4	4.5
Peru	-1.0	-0.9	-0.8	-0.9	-0.5	-0.5	-0.4	-0.5
Philippines	6.3	6.2	5.9	6.2	2.2	2.1	2.0	2.1
Rwanda	-7.4	-6.4	-5.3	-6.4	-4.2	-3.7	-3.1	-3.7
Senegal	5.3	5.4	5.4	5.4	-0.9	-0.8	-0.8	-0.8
Sierra Leone	-14.7	-15.6	-9.5	-13.6	-5.4	-6.2	-2.3	-4.8
South Africa	2.1	2.6	3.0	2.6	-2.2	-2.0	-1.7	-2.0
Tanzania	2.9	3.0	3.1	3.0	3.6	3.7	3.6	3.6
Togo	9.1	9.6	9.8	9.5	3.1	3.3	3.2	3.2
Turkey	11.6	11.5	10.2	11.2	10.4	9.0	6.3	8.6
Uganda	0.3	0.4	0.5	0.4	-0.9	-0.9	-0.8	-0.9
Vietnam	13.2	17.2	11.7	14.2	16.9	19.5	11.0	16.1
Yemen	10.2	10.3	9.8	10.1	10.6	10.3	8.8	10.0
Zambia	-2.3	-2.1	-2.0	-2.1	-6.0	-6.2	-6.2	-6.1
Zimbabwe	-2.1	-2.0	-2.0	-2.0	1.6	1.7	1.8	1.7

Note: N/A denotes not application and refers that the indicator has no sufficient data to examine trends

Supplement to: Hasan MM, Magalhaes RJS, Fatima Y, Ahmed S, Mamun AA. Levels, trends and inequalities in using institutional delivery services in low- and middle-income countries: a stratified analysis by facility type. *Glob Health Sci Pract*. 2021;9(1). https://doi.org/10.9745/GHSP-D-20-00533

Table S28: Change rates in the utilization of delivery services in public facilities in low- and middle-income countries by education

	Below secondary education				Secondary+ education			
**Country**	**1990-1999**	**2000-2009**	**2010-2018**	**1990-2018**	**1990-1999**	**2000-2009**	**2010-2018**	**1990-2018**
Afghanistan	-1.1	-2.2	-3.0	-2.1	2.8	3.2	2.2	2.8
Albania	0.6	0.3	0.1	0.3	0.0	-0.1	-0.8	-0.3
Angola	-1.9	-3.2	-4.2	-3.1	-0.2	-0.8	-3.3	-1.3
Armenia	14.0	0.4	0.0	4.7	0.2	0.1	0.1	0.1
Bangladesh	11.6	11.6	10.9	11.4	3.0	3.0	2.9	3.0
Benin	1.5	1.2	0.9	1.2	0.2	0.1	0.1	0.1
Bolivia	8.0	5.9	3.3	5.8	3.1	2.1	1.2	2.1
Brazil	2.5	0.6	0.2	1.1	0.8	0.1	-0.1	0.3
Burkina Faso	3.7	3.0	2.1	3.0	0.3	0.2	0.1	0.2
Burundi	15.0	14.3	5.8	12.0	4.1	3.0	1.3	2.9
Cambodia	26.9	23.1	8.6	20.1	10.7	8.2	4.4	7.9
Cameroon	-1.2	-1.2	-1.2	-1.2	-0.9	-1.0	-1.1	-1.0
Chad	4.6	4.7	4.6	4.6	0.4	0.4	0.4	0.4
Colombia	1.7	1.2	0.8	1.3	1.6	1.0	0.6	1.1
Comoros	5.6	3.9	2.2	3.9	1.4	0.9	0.6	1.0
Republic of the Congo	0.5	0.3	0.0	0.3	0.5	0.2	-0.1	0.2
Democratic Republic of the Congo	3.8	4.0	2.7	3.6	3.4	3.4	2.3	3.1
Cote d'Ivoire	1.7	1.5	1.3	1.5	-0.5	-0.6	-0.7	-0.6
Dominican Republic	0.9	0.6	0.4	0.6	1.0	0.9	0.7	0.9
Egypt	4.0	3.6	3.2	3.6	-0.6	-0.6	-0.6	-0.6
Ethiopia	15.0	15.5	13.7	14.8	6.1	4.7	2.8	4.6
Gabon	-0.3	-0.4	-0.5	-0.4	-0.3	-0.5	-0.7	-0.5
Ghana	4.1	3.6	2.8	3.5	1.9	1.5	1.1	1.5
Guatemala	4.7	4.0	3.0	3.9	1.2	1.0	0.8	1.0
Guinea	1.9	2.0	1.9	1.9	-1.0	-1.3	-1.7	-1.3
Haiti	4.7	4.6	4.4	4.6	2.7	2.5	2.2	2.5
Honduras	6.4	5.5	2.4	4.9	2.9	1.8	0.7	1.8
India	8.2	7.5	5.7	7.2	3.4	3.1	2.6	3.0
Indonesia	7.6	7.4	6.6	7.2	1.3	1.3	1.4	1.3
Jordan	0.3	0.3	0.2	0.3	0.6	0.5	0.5	0.5
Kazakhstan	0.0	0.0	0.0	0.0	0.0	-0.2	-0.5	-0.2
Kenya	2.0	2.0	1.8	1.9	0.7	0.7	0.6	0.7
Kyrgyz Republic	0.0	0.0	0.0	0.0	0.6	0.2	0.1	0.3
Lesotho	5.4	5.1	3.4	4.7	3.8	2.6	1.4	2.6
Liberia	12.7	12.3	7.8	11.1	2.4	2.4	2.0	2.3
Madagascar	-2.4	-2.5	-2.5	-2.5	0.2	0.2	0.2	0.2
Malawi	4.9	3.7	2.3	3.7	1.2	1.0	0.8	1.0
Maldives	0.5	0.3	0.1	0.3	-0.3	-0.6	-1.2	-0.7
Mali	5.6	4.8	3.4	4.7	0.7	0.5	0.4	0.5
Morocco	7.9	5.0	2.4	5.2	1.6	1.2	0.8	1.2
Mozambique	2.6	2.2	1.7	2.2	0.0	-0.1	-0.1	-0.1
Namibia	1.3	1.1	0.9	1.1	0.2	0.2	0.1	0.2
Nepal	13.1	12.4	9.9	11.9	5.5	4.7	3.5	4.6
Nicaragua	0.1	-0.3	-0.3	-0.2	-0.4	-2.3	-2.2	-1.6
Niger	3.4	3.4	3.1	3.3	0.0	-0.1	-0.1	-0.1
Nigeria	-2.4	-2.4	-2.3	-2.3	-2.5	-3.8	-4.8	-3.7
Pakistan	5.4	5.4	5.1	5.3	-0.5	-0.4	-0.3	-0.4
Peru	9.0	6.3	3.4	6.3	2.3	1.5	0.9	1.6
Philippines	7.3	6.8	5.7	6.6	4.9	4.2	3.2	4.1
Rwanda	12.1	8.0	3.3	8.0	3.4	2.0	1.0	2.2
Senegal	3.4	2.5	1.8	2.6	1.6	1.2	0.8	1.2
Sierra Leone	16.3	21.8	11.4	16.9	18.8	22.3	8.2	17.0
South Africa	3.0	1.5	0.7	1.8	0.8	0.6	0.4	0.6
Tajikistan	7.0	8.0	4.2	6.5	6.8	6.3	2.7	5.4
Tanzania	0.6	0.6	0.6	0.6	0.0	0.0	0.0	0.0
Timor-Leste	10.3	15.9	15.7	14.0	9.2	10.5	7.2	9.1
Togo	2.4	2.1	1.6	2.0	0.2	0.1	0.0	0.1
Turkey	1.3	1.1	0.8	1.1	-1.2	-1.7	-2.3	-1.7
Uganda	7.3	6.2	4.5	6.1	3.2	2.5	1.9	2.6
Vietnam	8.9	4.7	1.4	5.1	5.2	1.6	0.4	2.5
Yemen	2.6	2.7	2.7	2.7	-2.9	-4.7	-6.1	-4.5
Zambia	3.4	3.0	2.4	3.0	1.4	1.1	0.8	1.1
Zimbabwe	0.5	0.5	0.5	0.5	-0.2	-0.3	-0.3	-0.3

Supplement to: Hasan MM, Magalhaes RJS, Fatima Y, Ahmed S, Mamun AA. Levels, trends and inequalities in using institutional delivery services in low- and middle-income countries: a stratified analysis by facility type. *Glob Health Sci Pract*. 2021;9(1). https://doi.org/10.9745/GHSP-D-20-00533

Table S29: Change rates in the utilization of delivery services in private facilities in low- and middle-income countries by education

	Below secondary education				Secondary+ education			
**Country**	**1990-1999**	**2000-2009**	**2010-2018**	**1990-2018**	**1990-1999**	**2000-2009**	**2010-2018**	**1990-2018**
Afghanistan	-6.0	-11.8	-11.3	-9.7	-4.2	-8.2	-8.4	-6.9
Albania	N/A	N/A	N/A	N/A	22.4	24.7	25.8	24.3
Angola	-7.6	-6.3	-4.7	-6.3	1.1	2.8	4.3	2.7
Armenia	N/A	N/A	N/A	N/A	25.4	26.8	26.0	26.1
Bangladesh	18.0	18.1	16.8	17.7	7.6	7.2	6.2	7.0
Benin	0.9	1.0	1.1	1.0	-0.1	-0.1	0.0	-0.1
Bolivia	-4.5	-3.8	-3.1	-3.8	-1.9	-1.7	-1.3	-1.6
Brazil	1.4	5.7	5.0	4.1	-1.1	0.6	0.8	0.1
Burkina Faso	-0.3	0.6	1.4	0.6	2.7	3.3	3.7	3.2
Burundi	-7.3	-9.0	-7.5	-8.0	-4.6	-5.8	-4.7	-5.1
Cambodia	16.4	16.5	15.1	16.1	9.8	9.7	8.1	9.2
Cameroon	2.0	2.0	2.0	2.0	3.0	2.7	2.4	2.7
Chad	-3.9	-3.1	-2.2	-3.1	0.5	1.1	1.6	1.0
Colombia	-11.9	-11.3	-10.2	-11.2	-6.6	-7.1	-6.7	-6.8
Comoros	14.0	14.6	14.9	14.5	N/A	N/A	N/A	N/A
Republic of the Congo	4.9	7.5	8.4	6.9	4.7	7.3	8.0	6.7
Democratic Republic of the Congo	-4.4	-5.5	-4.4	-4.8	-2.4	-3.5	-3.4	-3.1
Cote d'Ivoire	12.2	12.9	13.0	12.7	5.1	5.4	5.4	5.3
Dominican Republic	-3.2	-3.2	-3.0	-3.2	-1.3	-1.4	-1.4	-1.4
Egypt	11.4	9.5	6.3	9.2	5.7	4.1	2.6	4.2
Ethiopia	13.3	14.1	14.8	14.0	8.9	9.7	9.8	9.5
Gabon	4.0	4.5	4.5	4.3	2.5	2.9	2.9	2.8
Ghana	-3.8	-3.8	-3.6	-3.7	-2.6	-2.7	-2.7	-2.6
Guatemala	0.0	0.4	0.8	0.4	-1.6	-1.6	-1.5	-1.5
Guinea	9.2	10.1	10.5	9.9	13.0	12.9	10.8	12.3
Haiti	-5.0	-4.7	-4.2	-4.6	-4.0	-4.8	-5.1	-4.6
Honduras	-7.3	-5.7	-2.1	-5.2	-5.6	-7.1	-5.0	-6.0
India	4.1	4.2	4.1	4.1	0.1	0.1	0.2	0.1
Indonesia	9.0	8.2	6.6	8.0	4.2	3.4	2.6	3.4
Jordan	2.8	2.8	2.6	2.7	0.2	0.2	0.2	0.2
Kenya	1.7	1.9	2.1	1.9	0.9	1.0	1.0	1.0
Lesotho	-2.7	-0.6	1.5	-0.7	6.5	8.8	10.1	8.4
Liberia	-1.0	-0.2	0.7	-0.2	0.8	1.4	1.9	1.4
Madagascar	6.4	7.0	7.4	6.9	5.4	5.4	5.2	5.3
Malawi	-0.4	-0.2	0.0	-0.2	-2.0	-2.1	-2.0	-2.0
Maldives	1.7	4.1	6.1	3.9	2.1	3.3	4.3	3.2
Mali	10.4	11.2	11.6	11.1	4.8	5.1	5.1	5.0
Morocco	5.1	6.0	6.6	5.9	-1.0	-0.7	-0.4	-0.7
Mozambique	3.5	4.1	4.8	4.1	-8.8	-7.4	-5.6	-7.4
Namibia	-10.4	-9.4	-8.4	-9.5	2.3	2.5	2.7	2.5
Nepal	13.8	14.0	13.6	13.8	4.6	4.7	4.6	4.7
Nicaragua	8.5	24.9	12.6	15.8	11.9	14.5	4.0	10.5
Niger	0.7	1.2	1.7	1.1	3.2	3.7	4.1	3.7
Nigeria	6.2	6.5	6.5	6.4	9.3	8.2	5.7	7.8
Pakistan	9.6	8.9	7.2	8.6	3.7	3.1	2.3	3.1
Peru	-1.1	-1.0	-0.9	-1.0	-1.4	-1.4	-1.3	-1.4
Philippines	5.7	5.7	5.7	5.7	1.8	1.8	1.7	1.8
Rwanda	-12.0	-11.1	-10.0	-11.1	-3.8	-3.6	-3.2	-3.6
Senegal	1.1	1.1	1.2	1.1	-3.6	-4.0	-4.1	-3.9
Sierra Leone	-7.6	-3.9	2.4	-3.3	-4.9	-15.9	-18.5	-13.0
South Africa	-8.1	-7.7	-7.3	-7.7	-2.2	-2.0	-1.7	-2.0
Tanzania	2.8	2.9	3.0	2.9	2.0	2.1	2.1	2.1
Togo	6.3	6.7	6.7	6.6	2.3	2.7	2.8	2.6
Turkey	11.2	10.4	8.3	10.1	8.1	6.8	4.8	6.7
Uganda	-0.3	-0.2	-0.1	-0.2	-1.1	-1.1	-1.1	-1.1
Vietnam	10.6	15.5	10.7	12.4	15.7	19.7	11.9	16.1
Yemen	10.1	10.1	9.6	10.0	8.5	8.2	7.0	7.9
Zambia	-4.1	-4.1	-4.0	-4.1	-4.9	-5.1	-5.1	-5.1
Zimbabwe	-2.8	-2.7	-2.6	-2.7	-0.9	-0.8	-0.7	-0.8

Note: N/A denotes not application and refers that the indicator has no sufficient data to examine trends

Supplement to: Hasan MM, Magalhaes RJS, Fatima Y, Ahmed S, Mamun AA. Levels, trends and inequalities in using institutional delivery services in low- and middle-income countries: a stratified analysis by facility type. *Glob Health Sci Pract*. 2021;9(1). https://doi.org/10.9745/GHSP-D-20-00533

Table S30: Change rates in the utilization of delivery services in public facilities in low- and middle-income countries by age

	Adolescents, 15-19 years of age				Adults, 20-49 years of age			
**Country**	**1990-1999**	**2000-2009**	**2010-2018**	**1990-2018**	**1990-1999**	**2000-2009**	**2010-2018**	**1990-2018**
Afghanistan	1.8	2.9	3.4	2.7	5.3	8.2	7.8	7.2
Albania	67.7	39.0	0.4	36.9	0.0	-0.1	-0.4	-0.2
Angola	-1.0	-1.6	-2.1	-1.6	-0.6	-0.7	-0.8	-0.7
Armenia	0.5	0.2	0.1	0.3	0.2	0.1	0.1	0.1
Bangladesh	9.8	9.6	8.7	9.4	9.3	9.1	8.4	8.9
Benin	1.5	1.2	0.9	1.2	1.4	1.2	0.9	1.2
Bolivia	4.3	2.9	1.7	3.0	5.5	4.0	2.4	4.0
Brazil	1.1	0.3	0.1	0.5	1.6	0.5	0.2	0.8
Burkina Faso	4.1	3.0	1.9	3.0	3.6	3.0	2.1	2.9
Burundi	7.3	5.8	2.4	5.3	14.3	13.7	5.5	11.5
Cambodia	18.2	15.2	6.7	13.7	22.8	19.0	7.6	16.9
Cameroon	-0.7	-0.7	-0.7	-0.7	-0.8	-0.8	-0.8	-0.8
Chad	4.3	4.4	4.1	4.3	4.9	4.9	4.7	4.9
Colombia	1.1	0.7	0.5	0.8	1.4	1.0	0.7	1.1
Comoros	5.3	3.4	1.8	3.5	5.4	3.5	1.9	3.6
Republic of the Congo	-0.4	-0.9	-1.8	-1.0	1.1	0.8	0.4	0.8
Democratic Republic of the Congo	7.0	6.7	3.8	6.0	3.7	3.8	2.6	3.4
Cote d'Ivoire	0.7	0.6	0.6	0.6	1.6	1.4	1.2	1.4
Dominican Republic	0.5	0.4	0.3	0.4	0.3	0.2	0.2	0.2
Egypt	0.4	0.5	0.6	0.5	1.8	1.8	1.7	1.8
Ethiopia	10.8	11.3	9.9	10.7	12.7	12.8	11.2	12.3
Gabon	0.7	0.5	0.3	0.5	-0.3	-0.5	-0.6	-0.5
Ghana	3.8	3.1	2.3	3.1	4.9	3.8	2.6	3.8
Guatemala	4.6	3.6	2.5	3.6	4.4	3.6	2.7	3.6
Guinea	0.0	0.1	0.2	0.1	1.8	2.0	1.9	1.9
Haiti	5.5	5.2	4.5	5.1	5.8	5.5	4.8	5.4
Honduras	5.0	3.6	1.4	3.4	7.3	5.8	2.2	5.2
India	7.9	6.8	4.9	6.6	6.8	6.1	4.6	5.9
Indonesia	6.2	6.0	5.4	5.9	5.0	4.8	4.4	4.8
Jordan	-0.3	-0.4	-0.4	-0.4	0.5	0.5	0.4	0.5
Kazakhstan	0.0	0.0	0.0	0.0	0.0	-0.2	-0.5	-0.2
Kenya	2.5	2.3	1.9	2.3	1.7	1.7	1.6	1.6
Kyrgyz Republic	0.0	0.0	0.0	0.0	0.7	0.2	0.1	0.3
Lesotho	7.7	5.7	2.5	5.4	6.2	5.1	2.8	4.8
Liberia	14.1	12.7	7.2	11.5	10.1	9.6	6.5	8.9
Madagascar	-1.7	-1.7	-1.7	-1.7	-1.7	-1.8	-1.8	-1.8
Malawi	5.3	3.6	2.0	3.7	4.7	3.5	2.2	3.5
Maldives	-0.9	-2.4	-4.9	-2.6	-0.2	-0.6	-1.3	-0.7
Mali	5.9	4.6	2.9	4.5	5.4	4.7	3.3	4.5
Morocco	6.0	3.3	1.5	3.7	7.1	4.5	2.2	4.7
Mozambique	3.3	2.5	1.7	2.5	3.1	2.6	1.9	2.5
Namibia	0.5	0.4	0.3	0.4	1.6	1.1	0.8	1.2
Nepal	15.1	13.1	7.8	12.2	12.9	11.8	8.5	11.2
Nicaragua	0.4	-0.8	-0.7	-0.4	0.1	-0.8	-0.6	-0.5
Niger	3.4	3.3	2.9	3.2	3.4	3.3	3.1	3.3
Nigeria	-1.4	-1.4	-1.3	-1.4	-1.4	-1.4	-1.3	-1.4
Pakistan	3.9	3.9	3.7	3.8	4.1	4.1	3.9	4.0
Peru	6.4	3.7	1.7	4.0	5.2	3.2	1.7	3.5
Philippines	7.7	6.3	4.3	6.2	6.0	5.2	4.0	5.1
Rwanda	12.7	6.0	1.5	6.9	11.3	7.4	3.0	7.4
Senegal	3.5	2.5	1.6	2.6	3.3	2.5	1.7	2.5
Sierra Leone	16.9	22.2	10.1	16.9	17.4	22.7	11.1	17.5
South Africa	1.7	0.7	0.2	0.9	1.2	0.8	0.6	0.9
Tajikistan	7.9	6.2	1.8	5.5	7.1	6.7	2.9	5.7
Tanzania	0.8	0.8	0.7	0.8	0.8	0.8	0.7	0.8
Timor-Leste	10.6	15.5	12.8	13.1	11.0	15.2	12.1	12.9
Togo	2.5	2.0	1.4	2.0	2.4	2.1	1.6	2.1
Turkey	0.6	0.5	0.4	0.5	0.3	0.3	0.2	0.3
Uganda	6.3	4.9	3.2	4.9	7.1	6.0	4.3	5.8
Vietnam	10.9	4.1	0.9	5.4	5.9	2.4	0.7	3.1
Yemen	2.6	2.7	2.6	2.6	2.4	2.6	2.6	2.5
Zambia	4.1	3.0	1.9	3.0	3.0	2.6	2.0	2.6
Zimbabwe	0.5	0.5	0.4	0.5	0.6	0.5	0.5	0.5

Supplement to: Hasan MM, Magalhaes RJS, Fatima Y, Ahmed S, Mamun AA. Levels, trends and inequalities in using institutional delivery services in low- and middle-income countries: a stratified analysis by facility type. *Glob Health Sci Pract*. 2021;9(1). https://doi.org/10.9745/GHSP-D-20-00533

Table S31: Change rates in the utilization of delivery services in private facilities in low- and middle-income countries by age

Country	Adolescents, 15-19 years of age				Adults, 20-49 years of age			
	1990-1999	2000-2009	2010-2018	1990-2018	1990-1999	2000-2009	2010-2018	1990-2018
Afghanistan	-3.2	-2.2	2.3	-1.2	-4.7	-4.2	0.2	-3.1
Albania	N/A	N/A	N/A	N/A	28.0	30.8	32.2	30.3
Angola	2.1	4.3	6.2	4.1	-2.2	-0.4	1.3	-0.5
Armenia	7.6	10.0	11.6	9.7	24.4	25.9	25.4	25.3
Bangladesh	17.9	17.5	14.7	16.8	14.3	13.7	11.6	13.3
Benin	1.0	1.2	1.4	1.2	1.4	1.5	1.6	1.5
Bolivia	-3.4	-2.8	-2.1	-2.8	-2.6	-2.0	-1.3	-2.0
Brazil	11.7	12.7	7.2	10.8	16.8	9.0	2.6	9.7
Burkina Faso	4.9	5.7	6.4	5.6	1.8	2.2	2.5	2.1
Burundi	-4.4	-4.0	-1.5	-3.4	-6.8	-8.1	-6.2	-7.1
Cambodia	9.9	10.3	9.6	10.0	15.8	15.9	13.9	15.3
Cameroon	3.0	3.0	2.8	3.0	3.1	2.9	2.7	2.9
Chad	-3.8	-3.0	-2.1	-3.0	-0.8	0.1	1.0	0.1
Colombia	2.9	3.3	3.4	3.2	-5.4	-5.1	-4.3	-5.0
Comoros	N/A	N/A	N/A	N/A	20.1	20.6	20.2	20.3
Republic of the Congo	11.6	14.4	12.5	12.9	4.2	6.6	7.5	6.1
Democratic Republic of the Congo	-3.9	-7.1	-7.8	-6.2	-3.1	-3.8	-2.8	-3.3
Cote d'Ivoire	11.9	12.9	13.4	12.7	9.7	10.3	10.4	10.1
Dominican Republic	-1.6	-1.4	-1.2	-1.4	0.1	0.2	0.2	0.2
Egypt	13.9	9.8	4.2	9.5	9.8	7.3	4.1	7.2
Ethiopia	9.1	11.1	12.8	10.9	11.8	12.6	13.3	12.5
Gabon	-1.4	-0.9	-0.3	-0.9	3.8	4.2	3.9	4.0
Ghana	-1.2	-0.8	-0.5	-0.9	-1.4	-1.3	-1.2	-1.3
Guatemala	0.5	0.8	1.1	0.8	1.5	1.7	1.9	1.7
Guinea	8.9	10.0	10.6	9.8	11.2	12.1	12.3	11.8
Haiti	-6.6	-7.2	-7.2	-7.0	-1.6	-1.3	-0.9	-1.3
Honduras	1.5	6.4	9.9	5.8	-5.1	-3.8	-0.1	-3.1
India	4.2	4.1	3.8	4.1	3.6	3.5	3.2	3.5
Indonesia	10.2	9.1	6.6	8.7	7.8	6.5	4.6	6.4
Jordan	1.4	1.4	1.3	1.4	0.9	0.9	0.9	0.9
Kenya	0.2	0.3	0.5	0.3	1.9	2.0	2.1	2.0
Lesotho	-3.1	-2.0	-0.9	-2.1	5.3	7.8	9.4	7.4
Liberia	0.6	1.3	2.0	1.3	2.1	3.3	4.2	3.2
Madagascar	8.5	9.1	9.5	9.0	4.8	5.2	5.5	5.2
Malawi	-0.7	-0.6	-0.4	-0.6	-0.3	-0.1	0.2	-0.1
Maldives	6.5	7.3	5.9	6.6	4.4	6.4	7.5	6.0
Mali	5.3	6.0	6.6	6.0	10.5	11.1	11.3	10.9
Morocco	-5.0	-3.9	-2.8	-4.0	5.0	5.6	5.5	5.4
Mozambique	2.1	3.8	5.5	3.7	9.3	10.1	10.8	10.1
Namibia	6.5	7.2	7.6	7.1	4.3	4.6	4.8	4.6
Nepal	12.7	13.0	12.5	12.8	12.7	12.7	12.0	12.5
Nicaragua	2.2	16.4	10.2	9.8	13.8	19.9	6.1	13.8
Niger	-1.2	-0.8	-0.3	-0.8	2.7	3.2	3.8	3.2
Nigeria	10.0	10.3	9.9	10.1	9.5	9.3	8.0	9.0
Pakistan	8.9	8.1	6.4	7.9	8.3	7.4	5.7	7.2
Peru	0.3	0.5	0.7	0.5	-0.1	0.0	0.1	0.0
Philippines	4.3	4.3	4.1	4.2	3.1	3.1	2.9	3.0
Rwanda	3.8	10.5	14.6	9.5	-4.3	-3.9	-3.4	-3.9
Senegal	1.4	1.5	1.7	1.5	0.5	0.5	0.5	0.5
Sierra Leone	-8.7	-6.4	-0.7	-5.5	-8.9	-8.9	-4.3	-7.6
South Africa	-8.6	-8.3	-7.9	-8.3	-0.5	-0.2	0.2	-0.2
Tanzania	2.7	2.9	3.0	2.8	3.2	3.3	3.3	3.3
Togo	4.5	5.1	5.4	5.0	6.2	6.6	6.5	6.4
Turkey	7.0	6.7	5.8	6.6	11.6	10.4	7.5	9.9
Uganda	-1.7	-1.6	-1.5	-1.6	0.6	0.7	0.8	0.7
Vietnam	N/A	N/A	N/A	N/A	13.2	17.4	11.7	14.3
Yemen	10.6	10.5	9.6	10.3	10.8	10.7	9.9	10.5
Zambia	-3.8	-3.8	-3.7	-3.8	-4.1	-4.1	-4.0	-4.1
Zimbabwe	-1.7	-1.5	-1.4	-1.5	-1.2	-1.1	-0.9	-1.1

Note: N/A denotes not application and refers that the indicator has no sufficient data to examine trends

Reference

1. Rahman MS, Rahman MM, Gilmour S, Swe KT, Krull Abe S, Shibuya K. Trends in, and projections of, indicators of universal health coverage in Bangladesh, 1995–2030: a Bayesian analysis of population-based household data. *Lancet Glob Heal*. 2018;6(1):e84-e94. doi:10.1016/S2214-109X(17)30413-8

2. Gelman A, Carlin J, Stern H, Dunson D, Vehtari A, Rubin D. *Bayesian Data Analysis*. London; 2013.

3. Plummer M. *Package 'Rjags'. The Comprehensive R Archive Network*.; 2013. http://cran.r-project.org/.
